# Computational identification of developmental enhancers: conservation and function of transcription factor binding-site clusters in *Drosophila melanogaster *and *Drosophila pseudoobscura*

**DOI:** 10.1186/gb-2004-5-9-r61

**Published:** 2004-08-20

**Authors:** Benjamin P Berman, Barret D Pfeiffer, Todd R Laverty, Steven L Salzberg, Gerald M Rubin, Michael B Eisen, Susan E Celniker

**Affiliations:** 1 Department of Molecular and Cell Biology, University of California, Berkeley, CA 94720, USA; 2 Berkeley *Drosophila *Genome Project, Genome Sciences Department, Life Sciences Division, Lawrence Orlando Berkeley National Laboratory, Berkeley, CA 94720, USA; 3 Howard Hughes Medical Institute, Department of Molecular and Cell Biology, University of California, Berkeley, CA 94720, USA; 4 The Institute for Genomic Research, 9712 Medical Center Drive, Rockville, MD 20878, USA; 5 Genome Sciences Department, Genomics Division, Lawrence Orlando Berkeley National Laboratory, Berkeley, CA 94720, USA; 6 Center for Integrative Genomics, University of California, Berkeley, CA 94720, USA

## Abstract

**Background:**

The identification of sequences that control transcription in metazoans is a major goal of genome analysis. In a previous study, we demonstrated that searching for clusters of predicted transcription factor binding sites could discover active regulatory sequences, and identified 37 regions of the *Drosophila melanogaster *genome with high densities of predicted binding sites for five transcription factors involved in anterior-posterior embryonic patterning. Nine of these clusters overlapped known enhancers. Here, we report the results of *in vivo *functional analysis of 27 remaining clusters.

**Results:**

We generated transgenic flies carrying each cluster attached to a basal promoter and reporter gene, and assayed embryos for reporter gene expression. Six clusters are enhancers of adjacent genes: *giant*, *fushi tarazu*, *odd*-*skipped*, *nubbin*, *squeeze *and *pdm2*; three drive expression in patterns unrelated to those of neighboring genes; the remaining 18 do not appear to have enhancer activity. We used the *Drosophila pseudoobscura *genome to compare patterns of evolution in and around the 15 positive and 18 false-positive predictions. Although conservation of primary sequence cannot distinguish true from false positives, conservation of binding-site clustering accurately discriminates functional binding-site clusters from those with no function. We incorporated conservation of binding-site clustering into a new genome-wide enhancer screen, and predict several hundred new regulatory sequences, including 85 adjacent to genes with embryonic patterns.

**Conclusions:**

Measuring conservation of sequence features closely linked to function - such as binding-site clustering - makes better use of comparative sequence data than commonly used methods that examine only sequence identity.

## Abstract 2

27 predicted gene-regulatory regions in the *Drosophila melanogaster* genome were analyzed *in vivo*, confirming 15 active enhancer regions. A comparison with *Drosophila pseudoobscura* sequences revealed that conservation of binding-site clusters accurately discriminates functional regions from non-functional ones.

## Background

The transcription of protein-coding genes in distinct temporal and spatial patterns plays a central role in the differentiation and development of animal embryos. Decoding how the unique expression pattern of every transcript is encoded in DNA is essential to understanding how genome sequences specify organismal form and function.

Understanding gene regulation requires discovering the *cis*-acting sequences that control transcription, identifying which *trans*-acting factors act on each regulatory sequence, and determining how these interactions affect the timing and organization of transcription. The first step in this process is by no means straightforward. Regulatory regions are often large and complex. Functional *cis*-acting sequences are found 5' and 3' of transcripts and in introns, and can act over short or long distances. Most of the described animal regulatory sequences were identified by experimental dissection of a locus, and astonishingly few of these are well characterized.

Despite the paucity of good examples, as multiple regulatory sequences from different organisms were identified and characterized, some common features became apparent [1,2]. Most animal regulatory sequences act as compact modular units, with regions of roughly a kilobase (kb) in size controlling specific aspects of a gene's transcription. These regulatory units - referred to here as *cis*-regulatory modules (CRMs) - tend to contain functional binding sites for several different transcription factors, often with multiple sites for each factor.

As the first animal genome sequences were completed [3-6], researchers began to tackle the challenge of identifying regulatory sequences on a genomic scale. We and several other groups began to ask whether common characteristics of regulatory sequences - modularity and high binding-site density - might be distinguishing characteristics that would permit the computational identification of new regulatory sequences. A number of *in silico *methods to identify regulatory sequences on the basis of binding-site clustering have been developed and applied to animal genomes [7-10]. Some of the predictions have the expected *in vivo *regulatory activity [11-17], yet few of these predictions have been systematically evaluated.

The transcriptional regulatory network governing early *Drosophila *development is perhaps the best system in which to apply and evaluate these methods. Development of the *Drosophila *embryo is arguably better understood than that of any other animal. Sophisticated genetic screens [18,19] have identified most of the key regulators of early development, and the molecular biology and biochemistry of these factors and their target sequences have received a great deal of attention. The spatial and temporal embryonic expression patterns of a large number of genes are known from microarray [20] and *in situ *expression studies [21]. Transcriptional regulation plays a uniquely important role in pre-gastrula patterning, as most of the key events occur in the absence of cell membranes and the cell-cell signaling systems that play a crucial role later in fly development and throughout the development of most other animals.

In a previous study [11], we identified 37 regions of the *Drosophila melanogaster *genome with unusually high densities of predicted binding sites for the early-acting transcription factors Bicoid (BCD), Hunchback (HB), Krüppel (KR), Knirps (KNI) and Caudal (CAD). As nine of these regions overlapped previously known CRMs, we proposed the remaining 28 as predicted CRMs (pCRMs). We tested one of the previously untested pCRMs for enhancer activity in a standard reporter gene assay [22,23] and showed that it is responsible for directing a portion of the embryonic expression pattern of the gap transcription factor gene *giant *(*gt*) in a posterior stripe. Here, we report the systematic testing of the remaining 27 untested pCRMs for enhancer activity, resulting in collections of both *bona fide *positive and false-positive predictions, allowing us to develop and evaluate methods to improve the accuracy of methods for identifying functional *cis*-regulatory sequences.

We were particularly interested in methods based on the comparison of genome sequences of related species. The genome sequence of *D. pseudoobscura *(which diverged from *D. melanogaster *approximately 46 million years ago [24]) was recently completed by the Baylor Human Genome Sequencing Center, and several other *Drosophila *species are currently being sequenced. The morphological and molecular events in early embryonic development are highly conserved among drosophilids, and we expect the activity of the transcriptional regulators and the architecture of regulatory networks to be highly conserved as well. Most *D. melanogaster *regulatory sequences should have functional orthologs in other *Drosophila *species [25,26], and a major rationale for sequencing other *Drosophila *species is the expectation that regulatory sequences have characteristic patterns of evolution that can be used to identify them and to better understand their function.

Most methods used to identify regulatory sequences from interspecies sequence comparison are fairly simple. They identify 'conserved' non-coding sequences (CNSs), operationally defined as islands of non-coding sequence with relatively high conservation flanked by regions of low conservation, and assume that this conservation reflects regulatory function. Although crude, these methods have been remarkably effective in identifying mammalian regulatory sequences [27,28], and preliminary studies in *Drosophila *suggest that similar methods will be valuable in insects as well [29]. However, despite such successes, the extent of the efficacy of comparative sequence analysis in regulatory sequence discovery remains unclear. A systematic comparison of human-mouse sequence conservation in known regulatory regions and ancestral repeats (which provide a model for neutral evolution) suggests that regulatory regions cannot generally be distinguished on the basis of simple sequence conservation measures alone [30,31]. Similarly, a recent analysis of *D. melanogaster *and *D. pseudoobscura *showed that known regulatory regions are only slightly more conserved than the rest of the non-coding genome [32], highlighting the need for further study and the development of comparative methods that go beyond measures of sequence identity.

## Results

### Expression patterns of pCRM containing transgenes

The 37 pCRMs are shown in Table 1. Each has been assigned an identifier (of the form PCEXXXX). The first nine overlap previously known enhancers of *runt *(*run*), *even-skipped *(*eve*), *hairy *(*h*), *knirps *(*kni*) and *hunchback *(*hb*). To determine whether any of the remaining 28 pCRMs also function as enhancers, we generated P-element constructs containing the pCRM sequence with minimal flanking sequence on both sides fused to the *eve *basal promoter and a *lacZ *reporter gene (see Materials and methods). As the margins of the tested sequences do not precisely correspond to the margins of the clusters, we assigned a unique identifier (of the form CEXXXX) to each tested fragment (identical CE and PCE numbers correspond to the same pCRM).

We successfully generated multiple independent transgenic fly lines for 27 of the 28 pCRMs. We repeatedly failed to generate transgenes containing CE8007. This sequence contains five copies of an approximately 358 base-pair (bp) degenerate repeat. One additional pCRM (CE8002) also contains tandem repeats. While we were able to generate transgenes for CE8002 and assay its expression, these two tandem repeat-containing pCRMs (CE8007 and CE8002) were excluded from subsequent analyses.

**Figure 1:**
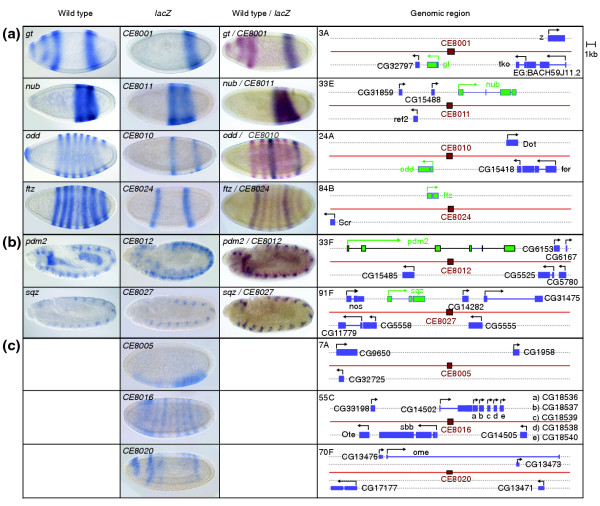
Expression patterns of active pCRMs. Embryonic whole-mount *in situ *RNA hybridizations using *lacZ *probe of transgenes with positive expression in independent lines (see Materials and methods). The first column (wild type) shows the endogenous gene expression; the second column (*lacZ*) shows transgene expression patterns; the third column shows double-labeled embryos with the endogenous (red) and transgene (blue) expression patterns. To the right of the images are maps of the gene regions centered on each pCRM.

We examined the expression of these constructs by *in situ *RNA hybridization to the *lacZ *transcript in embryos at different stages in at least three independent transformant lines. Nine of the 27 transgenes showed mRNA expression during embryogenesis (Figure 1), while the remaining 18 assayed transgenes showed no detectable expression at any stage during embryogenesis.

To identify the genes regulated by the nine pCRMs with embryonic expression, we examined the expression patterns of genes containing the pCRM in an intron and genes with promoters within 20 kb of the CRM (see Figure 1). We used the embryonic microrarray and whole-mount *in situ *expression data available in the Berkeley Gene Expression Database [21], supplemented with additional whole-mount *in situ *experiments where necessary (data not shown; these new *in situ*'s will be included in the public expression database [33] at its next release).

Six of the active pCRMs drive *lacZ *expression in patterns that recapitulate portions of the expression of a gene adjacent to or containing the pCRM. Four of these new enhancers act in the blastoderm and two during germ-band elongation.

CE8001 is 5' of the gene for the gap transcription factor *giant *and recapitulates the posterior domain (65-85% egg length measuring from the anterior end of the embryo) of *gt *expression in the blastoderm as previously described [11].

CE8011 is 5' of the gene for the POU-homeobox transcription factor nubbin (*nub*). The CRM recapitulates the endogenous blastoderm expression pattern of *nub*, first detected as a broad band extending from 50 to 75% egg length. Although *nub *expression continues in later embryonic stages, CE8011 expression is limited to the blastoderm stage.

CE8010 is 5' of the pair-rule gene *odd-skipped *(*odd*) and drives expression of two of its seven stripes: stripe 3 at 55% and stripe 6 at 75% egg length. This CRM also has the ability to drive later, more complex, patterns of expression. During stages 6 and 7, expression is detected in the procephalic ectoderm anlage and in the primordium of the posterior midgut. By stage 13, expression is also detected in the anterior cells of the midgut which will give rise to the proventriulus, the first midgut constriction, the posterior midgut and microtubule primordial as well as cells in the hindgut, all similar to portions of the pattern of wildtype *odd *protein expression previously described [34].

CE8024 is 3' of the pair-rule gene *fushi-tarazu *(*ftz*) and drives expression of two of its stripes: stripe 1 at 35% and stripe 5 at 65% egg length. Using a similar CRM reporter assay, this pattern of expression was also detected by [35].

CE8012 is in the third intron of *POU domain protein 2 *(*pdm2*) and appears to completely recapitulate its stage-12 expression pattern, which is limited to a subset of the developing neuroblasts and ganglion mother cells of the developing central nervous system. A similar pattern of expression was previously described for the protein product of *pdm2 *[36]. It is worth noting that we do not detect expression of CE8012 in the blastoderm stage, whereas the endogenous gene exhibits a blastoderm expression pattern similar to *nub*.

CE8027 is 3' of the gene for the Zn-finger transcription factor squeeze (*sqz*) and recapitulates the wild-type expression pattern of *sqz *RNA in a subset of cells in the neuroectoderm at stage 12. The wild-type *sqz *expression pattern was previously described [37].

The remaining three active pCRMs cannot be easily associated with a specific gene. CE8005 drives expression in the ventral region of the embryo. It is 3' of a gene encoding a ubiquitously expressed Zn-finger containing protein (*CG9650*) that is maternally expressed and deposited in the embryo. This strong maternal expression potentially obscures a zygotic expression pattern. Two additional adjacent genes, *CG32725 *and *CG1958*, showed no expression in whole-mount *in situ *hybridization of embryos.

CE8016 drives a seven-stripe expression pattern in the blastoderm. It is in the first intron of *CG14502 *which shows very low level expression by microarrays in the blastoderm, and has no obvious detectable pattern of expression in whole-mount *in situ *hybridization of embryos. This pCRM is approximately 2 kb 5' of *scribbler (sbb)*, which is expressed maternally, possibly obscuring an early zygotic expression pattern (a few *in situ *images show a hint of striping). *sbb *is also expressed later in development in the ventral nervous system. An additional potential target, *Otefin *(*Ote*), is also expressed maternally and relatively ubiquitously through germ-band extension. All other nearby genes displayed in Figure 1 showed no embryonic expression in whole-mount *in situ *hybridization or by microarray.

CE8020 drives an atypical four-stripe pattern in the blastoderm - two stripes at 7% and 26% that are anterior to the first *ftz *stripe and two stripes at 39% and 87%. It is in the first intron of *ome *(*CG32145*), which is not expressed maternally and has no blastoderm expression, but is expressed late in salivary gland, trachea, hindgut and a subset of the epidermis. All other nearby genes displayed in Figure 1 showed no embryonic expression in whole-mount *in situ *hybridization or by microarray.

With these results, and the nine previously known enhancers, at least 15 of the 37 highest density clusters of the five transcription factors used in our initial screen have early-embryonic enhancer activity. The remainder of this paper examines 35 of the original 37 clusters, with the two tandem repeat-containing clusters excluded. We divide these 35 into three categories - 15 positives (the nine overlapping previously known enhancers plus the six new enhancers identified here), three ambiguous (the three positives without a clear regulated gene), and 17 negatives (see Table 2). We largely focus on differences between the positives and negatives.

### Distinguishing active and inactive clusters

All 15 positives are within 20 kb of the transcription start site (or, where the transcription start site is unknown, the start of the gene annotation) of transcripts expressed in spatiotemporal patterns consistent with regulation by the maternal and gap transcription factors used in our screen (that is, in anterior-posterior patterns in the blastoderm or in the developing neuroblasts of the central nervous system). Only one of the 17 negatives was located within 20 kb of a plausible target (PCE8021 is 7 kb upstream of *reaper*), so out of 16 pCRMs located within 20 kb of a gene with appropriate expression, 15 (94%) are active enhancers.

**Figure 2:**
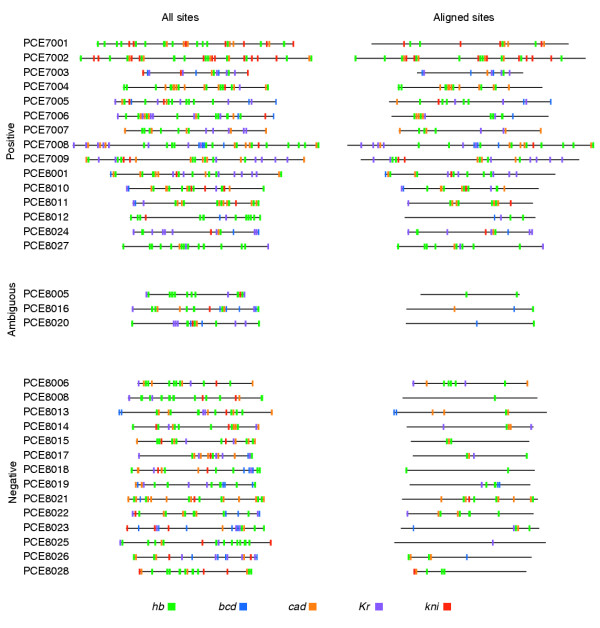
Predicted and aligned binding sites in pCRMs. Predicted binding sites and aligned binding sites (see Materials and methods) in positive, ambiguous and negative pCRMs (the positions of overlapping sites were adjusted slightly so that all sites could be seen).

The positives are, on average, larger than the negatives (average cluster size of positive = 900 bp, while average cluster size of negatives was 711 bp), a difference that is significant by the Komogorov-Smirnov (KS) test (*p *= 0.017). The positives have a slightly higher density of binding sites, but this difference was not significant. The binding site composition of the positives and negatives are similar (the positives contain more KR, and fewer BCD binding sites, but again these differences are not highly significant). Although others have reported that some factors have characteristic spacings with respect to themselves and other factors [38], we could not find evidence for such spacing or identify other differences that could distinguish positive pCRMs from negative (Figure 2).

### Use of *D. pseudoobscura*

We assembled the *D. pseudoobscura *genome from traces deposited in the NCBI's TraceDB using the Celera assembler [39,40]. These assemblies were used to examine the conservation of our pCRMs and to assess whether conservation could be used instead of or in addition to binding site clustering as a way to identify CRMs.

**Figure 3:**
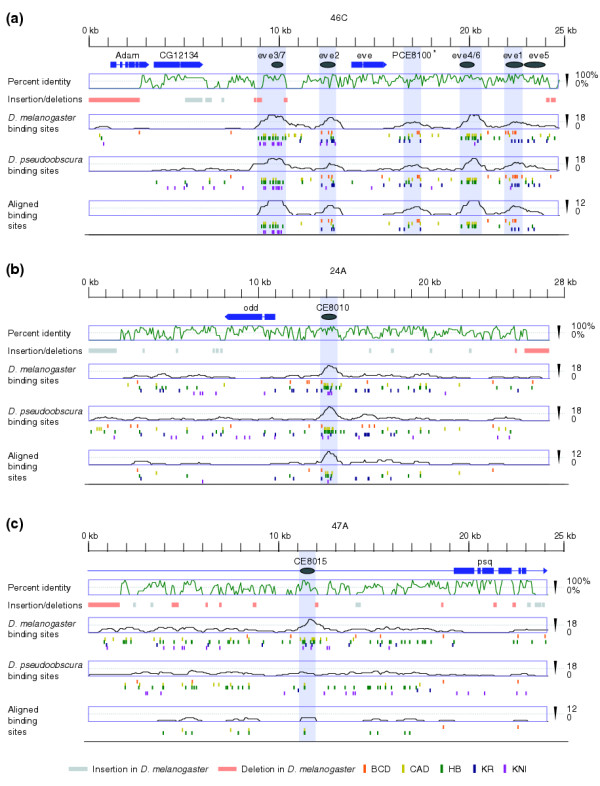
Binding-site conservation, but not sequence conservation, correlates with pCRM activity. Three 25-kb regions were chosen to illustrate patterns of sequence conservation and binding-site conservation. **(a)***even-skipped *(*eve*) contains five previously known segmentation enhancers (labeled eve3/7, eve2, eve4/6, eve1, and eve5); **(b)***odd-skipped *(*odd*) contains a single functional (positive) pCRM (CE8010); and **(c)***pipsqueak *(*psq*) contains a non-functional (negative) pCRM (CE8015). Annotated genes are shown in blue, and the direction of transcription is indicated by the arrow. Gray ovals indicate experimentally tested fragments, and shaded gray boxes show the extent of pCRMs as defined by CIS-ANALYST (minimum of 13 sites within a 700 bp window). The green graphs show average percent identity (in 100-bp windows). Below the percent identity plots are shown insertions (gray boxes) and deletions (orange boxes) of 80 or more bp in the *D. melanogaster *sequence relative to their *D. pseudoobscura *ortholog. The location of binding sites in *D. melanogaster*, binding sites in *D. pseudoobscura *and aligned binding sites along with the average density of sites (700-bp windows) are shown in the bottom three panels for each region. * in (a) indicates a new prediction (PCE8100).

We first assessed whether positive pCRMs could be distinguished from their flanking sequences based on degree of conservation. In vertebrate comparative genomics, relatively simple methods (such as VISTA [41]) are commonly used to identify CNSs that are a surprisingly rich source of new *cis*-regulatory sequences. We evaluated the potential of using such methods with *D. melanogaster *and *D. pseudoobscura *in two ways. First, we constructed percent-identity plots for the regions containing all of the 37 pCRMs (Figure 3; similar plots for all pCRMs are available in the online supplement at [42]) with the location of pCRMs and other known regulatory sequences clearly indicated. Although it appears that some CRMs (that is, *eve *stripe 3/7) would have been successfully identified by such simple comparative methods, positive pCRMs do not collectively appear distinguishable from flanking sequence on the basis of conservation alone. Although positive pCRMs are almost all in highly conserved blocks, there is a surprisingly high amount of non-coding sequence conservation throughout these regions, and most negative pCRMs are also contained in highly conserved blocks. It remains to be seen whether this difference in the conservation landscape of *Drosophila *non-coding sequences compared to vertebrates reflects a significant difference in the functional organization of non-coding sequences, or simply indicates that there is too little divergence between *D. melanogaster *and *D. pseudoobscura *to detect useful differences in the rates of evolution (see Discussion).

**Figure 4:**
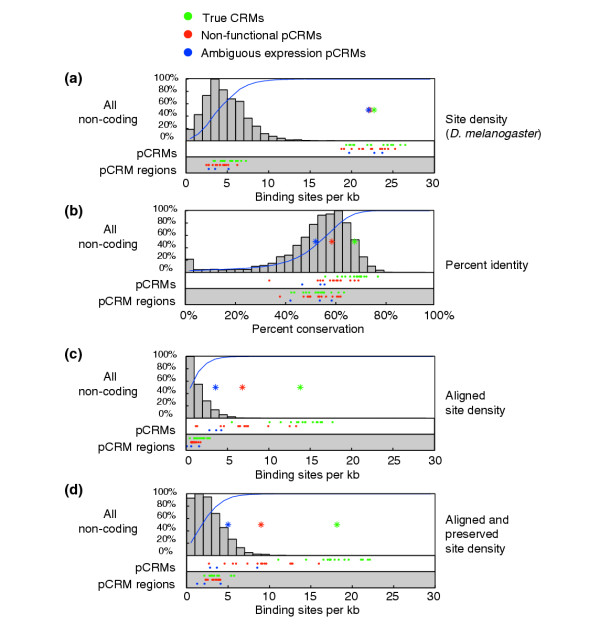
Conservation of clustering distinguishes positive and negative pCRMs. Each panel compares positive, negative and ambiguous pCRMs and random 1,000-bp non-coding regions based on **(a) **binding site density in *D. melanogaster*, **(b) **percent identity, **(c) **density of aligned sites, and **(d) **density of aligned plus preserved sites. The top portion of each panel contains a histogram of the values for randomly chosen 1,000-bp regions of the *D. melanogaster *genome. The blue line plots the cumulative distribution. The colored asterisks show the average values for each class of pCRM. The unshaded panel below the histogram shows the values for each pCRM (each dot represents one pCRM, with positives in blue, negatives in red, ambiguous in green). The shaded panel at the bottom shows the average value for 1,000-bp non-coding sequences within 20 kb of each pCRM.

We next assessed whether positive pCRMs can be distinguished from negative pCRMs on the basis of their degree of similarity between *D. melanogaster *and *D. pseudoobscura*. For each pCRM-containing region, we identified orthologous contigs from the *D. pseudoobscura *assembly and aligned them using the alignment program LAGAN [43]. We were able to find orthologous regions for 32 pCRMs (see Table 2). Using the simple measure of percent identity, we find that positive pCRMs are, on average, more highly conserved than negative pCRMs (see Table 2). Although this difference is significant (*p *= 0.002 by KS test), the distribution of conservation scores for positive and negative pCRMs overlap considerably, and thus conservation alone is not a useful way of distinguishing positive and negative pCRMs (see Figure 4b).

To get a genome-wide perspective on the degree of conservation in positive pCRMs, we analyzed the conservation of CRM-sized (1 kb) regions in randomly chosen sections of the genome (Figure 4b). Positive pCRMs are, generally, more conserved than average CRM-sized sequences, and some positive pCRMs are among the most highly conserved non-coding sequences in the genome. However, a conservation cut-off necessary to select the majority of positive pCRMs would select roughly one third of the non-coding regions of the genome, and thus is not a practical method for prioritizing sequences for functional analysis.

### Conservation of binding sites and conservation of clustering

We expect that most genes will have similar expression patterns in *D. melanogaster *and *D. pseudoobscura*, and that most *D. melanogaster *enhancers should have functional orthologs in *D. pseudoobscura*. For those enhancers we seek to identify here - namely those where binding site clustering reflects their function - we expect clustering to be found in both *D. melanogaster *and *D. pseudoobscura*. Conversely, clusters that simply occur by chance in either genome but do not reflect the function of the sequence (as, we believe, is the case for many of our false-positive predictions) should not be conserved. Thus, looking for conservation of binding-site clustering should provide a valuable way of distinguishing functional and non-functional binding-site clusters in the *D. melanogaster *genome.

We used the alignments described above to examine the conservation of individual predicted binding sites in all of the pCRMs (Table 2). We refer to a predicted *D. melanogaster *binding site that overlaps a predicted *D. pseudoobscura *binding site for the same factor in an alignment as an 'aligned' site. We require overlap and not perfect alignment to compensate for alignment ambiguity; the overwhelming majority (85%) of aligned sites are perfectly aligned. Although there is only a subtle difference in the binding-site density in the positive and negative pCRMs in *D. melanogaster *(22.7 sites/kb compared to 22.2), the density of aligned binding sites in positive pCRMs (13.8 sites/kb) is nearly twice that in negative pCRMs (6.8 sites/kb). This is a highly significant difference (*p *< 0.001 by KS test) and aligned site density better discriminates positive and negative pCRMs than sequence conservation (compare Figure 4c and 4b).

Sixty-one percent of the predicted binding sites in positive pCRMs are aligned, while only 30% of the sites in negative pCRMs are aligned. Across the genome, 22.3% of predicted binding sites are aligned meaning that there is a roughly fourfold increase over background in the probability that a binding site in a positive pCRM is conserved in place compared to a binding site in a negative pCRM. Sixty-one percent is almost certainly an underestimate of the fraction of pCRM sites that are functionally conserved. The *D. melanogaster*-*D. pseudoobscura *alignments were not always unambiguous (using simulations we have assessed the role of alignment algorithms in identifying conserved transcription factor binding sites, see [44]), and some orthologous binding sites may not have been properly aligned. More important, studies of the evolution of various *Drosophila *enhancers suggest that the positions of binding sites within an enhancer are somewhat plastic, and the functional conservation of a binding site does not necessarily require positional conservation [25,26].

To characterize the extent of binding site conservation independent of positional conservation, we computed a second measure of binding-site conservation. We consider an unaligned binding site in *D. melanogaster *to be 'preserved' if it can be matched to a corresponding site in the *D. pseudoobscura *pCRM (allowing each *D. pseudoobscura *site to match only one *D. melanogaste*r site). If we consider both aligned and preserved sites to be conserved, then roughly 80% of the sites in positive pCRMs are conserved compared with 40% in negative pCRMs.

The density of preserved but not aligned sites in positive pCRMs (4.3/kb) is considerably higher than in negative pCRMs (2.2/kb) or random sequences (1.8/kb). Thus, in the *D. pseudoobscura *orthologs of active *D. melanogaster *CRMs we observe an increase in binding-site density that cannot be explained by the positional conservation of sites found in *D. melanogaster *or the random occurrence of sites in the genome. Several of the 15 positive CRMs have high densities of these preserved but unaligned sites, but two in particular, *runt *stripe 3 and *hairy *stripe 6, stand out from the rest. These two have almost as many preserved sites as strictly aligned sites.

Aligned plus preserved (conserved) site density (Figure 4d) almost perfectly separates positive from negative pCRMs. Only one of the positive pCRMs (PCE8012) has a conserved site density below 14 sites/kb, while only one of the negative pCRMs (PCE8021) has a conserved site density above 14 sites/kb.

### eCIS-ANALYST: a comparative enhancer finder

As the conservation of binding sites and binding-site clusters between *D. melanogaster *and *D. pseudoobscura *successfully distinguishes positive and negative predictions made using the *D. melanogaster *sequence alone, we incorporated comparative sequence data into our enhancer-prediction algorithm CIS-ANALYST [11]. Instead of searching for clusters of predicted binding sites in a single genome, eCIS-ANALYST (the 'e' is for evolutionary) searches for conserved clusters of sites between the two genomes (see Materials and methods). eCIS-ANALYST is available at [45].

**Figure 5:**
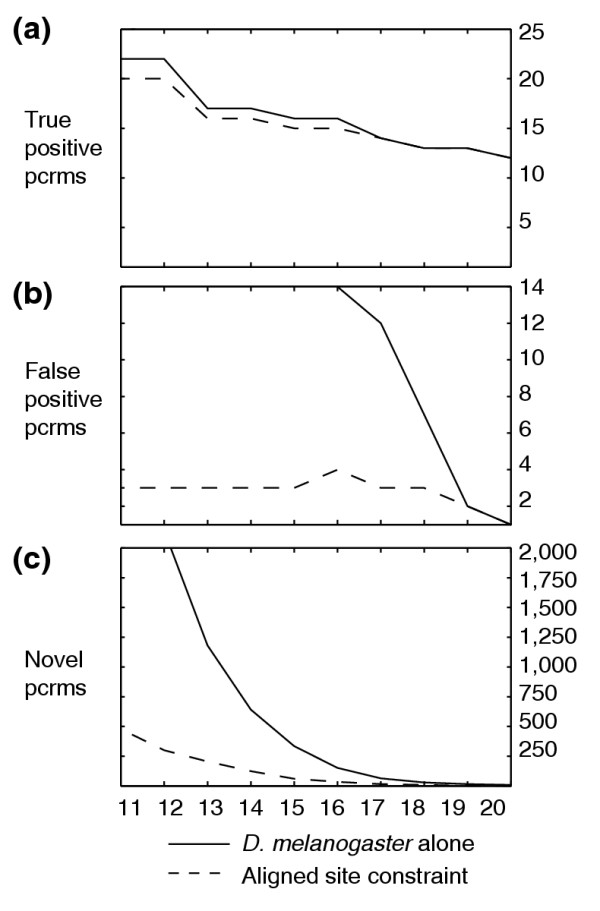
Inclusion of evolutionary information greatly increases the specificity and selectivity of CRM searches based on binding-site clustering. The effects of integrating comparative data into searches for binding site clusters were assessed by counting the number of **(a) **true positive, **(b) **negative and **(c) **novel CRMs recovered at the different site density cutoffs plotted on the *x*-axis. The positives used here include the 15 positive pCRMs from Table 2 and 10 additional positive CRMs from the literature (see text), all of which have identifiably orthologous sequence in *D. pseudoobscura*, while the negatives included only the 14 non-functional pCRMs for which orthologous sequence in *D. pseudoobscura *could be found. The solid line in each panel shows the results without the use of *D. pseudoobscura*; the dashed line shows the results with *D. pseudoobscura*. Searches displayed were performed using the aligned sites constraint (see Materials and methods). Comparable results were obtained for the aligned + preserved sites constraint. The number of false positives is not strictly monotonically decreasing with an increasing binding site cutoff. This stems from the cluster merging behavior of CIS-ANALYST - sometimes a decrease in the minimum number of sites leads CIS-ANALYST to tack on a lower-density cluster that is adjacent to a higher-density one, resulting in a single cluster with more sites but lower site density. This can actually increase the number of conserved sites necessary to reach the conservation threshold (see Materials and methods).

Using 17 negative pCRMs and an expanded set of 25 positive pCRMs (which included the 15 positive predictions discussed above and 10 functional enhancers known to respond to the five factors; these 10 additional enhancers were discussed and analyzed in [11] but had binding-site densities below the threshold used there), we compared the ability of CIS-ANALYST and eCIS-ANALYST to identify positive pCRMs and to distinguish positive and negative pCRMs at different binding-site density cutoffs (Figure 5). The incorporation of the conservation criteria greatly improves the algorithm's apparent performance. The expected fraction of false positives is markedly reduced, and it is possible to lower the binding site threshold to recover six of the ten previously missed positive enhancers without increasing the number of expected false-positive predictions.

### New predictions

As eCIS-ANALYST has markedly better specificity than CIS-ANALYST, we sought to identify BCD, HB, KR, KNI and CAD targets that were missed with the relatively stringent criteria used in our previous analysis. Rather than use a stringent cutoff (15 binding sites per 700 bp) as we did in [11], we performed three separate runs with lower cutoffs (for example, 10 sites per 700 bp in one run) and applied a conservation threshold (see Materials and methods and Additional data file 3) to select 929 conserved binding-site clusters. There were 842 new pCRMs within 20 kb or in an intron of an annotated transcript (Additional data file 7) and 87 more than 20 kb (Additional data file 8). We ranked these new pCRMs by a simple scoring scheme that measures both the density and the total number of sites conserved (we evaluated several different scoring schemes, and selected one that optimally identified regions near genes with blastoderm expression patterns; see Materials and methods). The 75 highest-scoring pCRMs within 20 kb of an annotated transcript are shown in Table 3. Thirteen of the 15 positive pCRMs described above are in the top 75 (*ftz *stripe 1/5 is number 107 and the *pdm2 *neurogenic enhancer is number 418) as are five other known enhancers. One of our negative pCRMs, CE8021, is ranked number 12.

**Figure 6:**
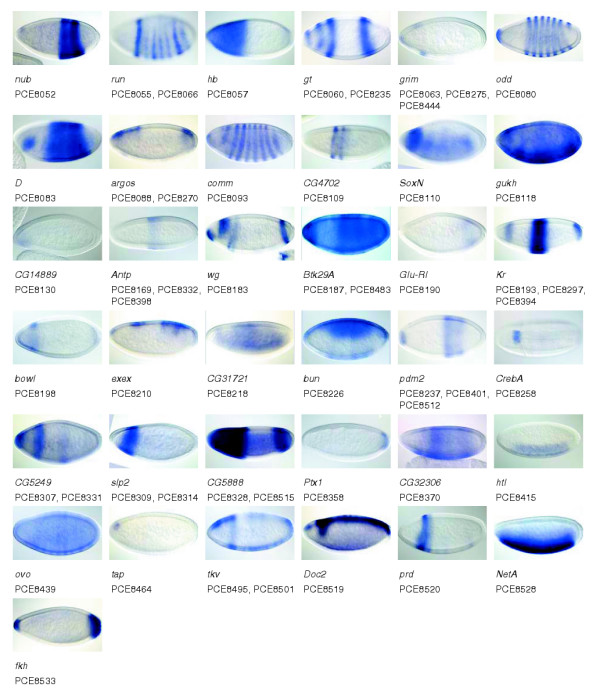
Expression patterns of genes adjacent to high-scoring pCRMs. Wild-type embryonic expression patterns of 36 genes adjacent to 53 pCRMs identified by eCIS-ANALYST (see Tables 3 and 4). The images were obtained from the BDGP Embryonic Expression Pattern Database [33], and include all pCRMs from Tables 3 and 4 for which an adjacent gene had an early segmentation pattern.

To focus our search for new enhancers on genes likely to be regulated by BCD, HB, KR, KNI and/or CAD, we searched FlyBase [46] and a database of *Drosophila *embryonic expression patterns [21] and identified 278 genes with anterior-posterior patterns in the blastoderm (AP genes; Figure 6 and see also Additional data files 2 and 9). Thirty-one of the 75 highest-scoring new predictions are adjacent to or within 20 kb of one or more of these genes, including 11 pCRMs that do not overlap previously described enhancers. The 75 highest-scoring predictions within 20 kb of an AP gene but not in Table 3, are shown in Table 4. In Tables 3 and 4 together, there are 106 high-scoring conserved binding-site clusters near AP genes, 90 of which do not overlap known enhancers.

## Discussion

We performed a large and comprehensive evaluation of the efficacy of computational methods for the identification of functional *cis*-regulatory modules in *Drosophila*. Analysis of the *in vivo *activity of 36 high-density clusters of predicted BCD, HB, KR, KNI and CAD binding sites identified in our previous study [11] offers compelling support for the use of transcription factor binding-site clustering as a method to identify regulatory sequences, as at least 15 of these sequences function as early developmental enhancers *in vivo*. An evolutionary analysis of these sequences - based on comparisons of the *D. melanogaster *and *D. pseudoobscura *genomes - shows that sequence conservation alone can not reliably discriminate cluster-containing regions that function *in vivo *from those that do not. However, a new method that combines binding-site clustering and comparative sequence analysis to search for binding-site clusters that are present in multiple species does reliably discriminate active and inactive clusters. Using this method, we make several hundred predictions of new CRMs, a large number of which are located near likely target genes.

### Binding-site clustering

The success of relatively simple binding-site clustering methods here and in other work is remarkable given the crudeness of these methods. As our negative predictions demonstrate, the mere presence of a cluster of binding sites is not sufficient to make an active embryonically expressed CRM. Although these 17 sequences have binding-site densities and compositions indistinguishable from their functional cousins, they do not function as enhancers in a simple transgene assay.

It is possible that some of these negative pCRMs may be functional enhancers that respond to the factors used in our screen, perhaps requiring a different promoter or other flanking sequences not used in the transgene. While further experiments could address this possibility, we felt these were a low priority, as few of the *D. pseudoobscura *orthologs of these negative pCRMs have binding-site clusters, and few are near genes with appropriate expression patterns. Thus it is unlikely that many function in their endogenous locations *in vivo*.

Both the general activity and, more important, the specific regulatory output of a CRM are a complex, and still poorly understood, function of the specific architecture of its sites. The emerging picture of the ordered multiprotein complexes that mediate enhancer activity suggests constraints on enhancer composition and architecture [1,2,47] whose elucidation will form a critical part of the future dissection of the function of *cis*-regulatory sequences.

It is intriguing that three of the clusters we tested direct expression patterns that bear no obvious relationship to the expression of a neighboring gene despite our extensive efforts to identify such genes. We cannot yet exclude the possibility that these pCRMs have an *in vivo *function related to their observed expression patterns. However, the poor conservation of these elements in *D. pseudoobscura *suggest that they do not have a regulatory function, and raises the possibility that some 'random' clusters of binding sites (that occur by chance or perhaps through selection on some functionally unrelated sequence feature) have the necessary characteristics to be active enhancers in the proper genomic environment (that is, near a promoter and not silenced by *trans*-acting chromatin mechanisms). That any such sequences exist suggests that the compositional and architectural constraints on binding sites in enhancers may be fairly weak.

Whatever the nature of these constraints, it is clear that binding-site density is not the sole defining characteristic of functional enhancers. However, it is a surprisingly effective distinguishing one, and the usefulness of this and related methods [48] suggests that the broader application of such methods to different collections of transcription factors will be extremely valuable in annotating the regulatory content of animal genomes.

### New enhancers

We identified double-stripe enhancers for *ftz *and *odd*. *ftz *and *odd *are generally classified as 'secondary' pair-rule genes whose expression is governed by other pair-rule genes rather than by the maternal and gap transcription factors that govern the so-called 'primary' pair-rule genes (*eve*, *h *and *runt*) ([49]; also reviewed in [50]). However, the *ftz *and *odd *enhancers described here were identified on the basis of binding sites for maternal and gap transcription factors, and function like the enhancers of primary pair-rule genes in directing expression in specific stripes.

It has been suggested that the *ftz *enhancer is an evolutionary relic of the homeotic role played by *ftz *in primitive insects [51], a view supported by the apparently normal expression and activity of ftz when this element is missing. However, given our observation that non-functional binding sites clusters are not conserved, even over the relatively short evolutionary distance separating *D. melanogaster *and *D. pseudoobscura*, it seems unlikely that this element is purely vestigial. In fact, Yu and Pick [52] examined the expression pattern of the endogenous *ftz *gene and show that stripes 1 and 5 appear before other *ftz *stripes and they postulate the existence of stripe-specific regulatory elements that may exist outside of the characterized *zebra *and upstream elements such as the one identified and characterized in this study. The conservation of binding sites in both the *ftz *and *odd *enhancers suggest that they play an important role in development, and further call into question the distinction between primary and secondary pair-rule genes.

Two of the new enhancers (CE8011 and CE8012) are adjacent to and apparently regulate two linked genes with very similar patterns of embryonic expression. Both *nub *(also known as *pdm1*) and *pdm2 *are expressed in the anterior and posterior midgut primordium and in neuroblasts. CE8011, found immediately upstream of *nub*, regulates its early expression, and not its later neuroblast expression. In contrast, CE8012, found in an intron of *pdm2 *regulates its expression only in neuroblasts and not earlier. While we did not detect a neuroblast enhancer for *nub *or a blastoderm enhancer for *pdm2 *in our single-species binding-site cluster search, a number of interesting *pdm2 *regions were discovered in our eCIS-ANALYST search (two are listed in Table 4).

### Regulatory models and improving the accuracy of CRM prediction

The accuracy of our enhancer predictions would almost certainly be improved if we restricted our search space to genomic regions adjacent to genes known to be regulated by particular transcription factors. *Drosophila *enhancers have been known to work at distances of up to 100 kb, but most are within 10 kb of their target gene. All of our true-positive predictions were within 10 kb of the known or predicted transcription start site of a gene with a pattern that was known, or plausibly could have been, regulated by the five regulators used in our screen (anterior-posterior patterns in the blastoderm; expression in neuroblasts). In contrast, only one of the negative predictions was this close to such a gene - an additional four were within 50 kb. As the comprehensive atlas of embryonic expression patterns is completed [21,53] it will be possible to restrict searches for CRMs to regions of the genome near genes with expression patterns that could arise from the regulators being considered, or to prioritize the results of whole-genome screens on the basis of whether they are near plausible targets.

Comprehensive methods for inferring regulatory interactions where they are not already known will be critical for the widespread application of binding-site clustering methods. In addition to allowing less stringent focused screens, they will also help overcome the combinatorial challenge raised by the existence of up to 700 sequence-specific transcription factors in *Drosophila*. Even assuming the availability of binding data for all of these factors, it will not be possible to search for targets of all combinations of these factors - there are too many possibilities. This is not just a practical problem - it is a fundamental statistical problem. While the false-positive rate for a single combination of factors is low, if we tried even all pairs of factors, it is likely that every region of the genome would have a high binding-site density for some collection of factors. Sequence data from other *Drosophila *species may allow us to determine which of these collections are conserved and therefore likely to be functional, but it is unlikely that all aspects of regulation can be inferred from comparative analyses and therefore it is essential that we continue to dissect the regulatory network by traditional means.

A greater current limitation in the widespread application of binding-site clustering methods is the absence of high-quality binding data for most *Drosophila *transcription factors. The initial success of methods that use *in vitro *binding data to predict regulatory targets has prompted the characterization of binding specificities for many additional factors. However, the heterogeneity of approaches used makes it difficult to combine these data in an optimal manner. In addition, most of the available transcription factor binding data consists of a few to several dozen high-affinity sites. While these data are very useful, they do not fully represent the binding capacity of a factor and thus do not permit the identification of intermediate or low-affinity sites which are known to be important in some regulatory systems [54]. We have begun to apply high-throughput methods [55] to characterize a broad spectrum of target sites for all of the transcription factors involved in early embryogenesis. The results will ultimately allow us to estimate the binding affinity of each factor for any target sequence.

### Comparative genomics in CRM predictions

The extent of non-coding sequence conservation between *D. melanogaster *and *D. pseudoobscura *was surprising. A major motivation for the National Human Genome Research Institute (NHGRI) support of the *D. pseudoobscura *genome sequencing was the identification of conserved regions that would guide the annotation of functional sequences in *D. melanogaster*. *D. pseudoobscura *was chosen as the second member of this genus to be sequenced in part because it was felt that it had separated from *D. melanogaster *sufficiently long ago that non-functional sequences would exhibit substantial divergence. However, despite an evolutionary separation that is greater than human and mouse (an average synonymous substitution rate of 1.8-2.6 substitutions/site [29] compared to 0.6 substitutions/site [30]), and despite some variation in conservation in non-coding sequences, we were not able to use standard measures of sequence conservation to differentiate active pCRMs from their flanking sequence or from inactive pCRMs, reinforcing other recent observations [32].

One reason for the limited efficacy of these methods is that they do not recognize the specific patterns of conservation characteristic of different classes of functional sequences. For example, coding sequences can be easily recognized from the characteristic triplet pattern in evolutionary rates where the third (and often synonymous) position of codons tends to evolve at a greater rate than the first two positions [56,57]. Similarly, RNAs that form conserved secondary structures can be recognized by patterns of co-substitution ([58] and references cited within). The early developmental enhancers we are studying here are made up of large collections of transcription factor-binding sites, and it is expected that both individual functional binding sites and the overall composition of functional CRMs will be conserved [25,26]. Conservation of binding-site clustering is a specific evolutionary signature of this class of functional regulatory sequences, and, like the evolutionary signatures of protein-coding and RNA genes, can be used to specifically identify these sequences from comparative sequence data.

Contrast PCE8010 (the *odd *stripe enhancer) and PCE8015 (Figure 3). Both have the same overall amount of sequence conservation, indicating that they are under some functional constraint. However, 80% of the predicted binding sites in PCE8001 are conserved, compared to 20% for PCE8015. The conservation of binding sites (both number and location) in PCE8001 makes it highly unlikely that the cluster was found by chance in *D. melanogaster*, and suggests (correctly) that this sequence is actively responding to the presence of these binding sites. The poor conservation of binding sites in PCE8015 (no greater than is found in random regions of genome) suggests either that the BCD, HB, KR, KNI and CAD sites in this region are not functional or that the region is undergoing rapid functional diversification. Of course the absence of binding site conservation does not suggest that the sequence is non-functional, merely that these sequences are unlikely to have the particular function we are studying here.

From the data shown in Figure 4, we expect the incorporation of binding-site conservation into the CRM search process to greatly reduce the number of false-positive predictions. We anticipate that a significant number of the new predictions from our genome-wide screen and screen targeted at genes with early anterior-posterior patterns to be active CRMs, and we have begun testing these predictions.

The pattern of binding-site conservation in positive pCRMs sheds additional light on the processes that govern CRM evolution. We find that predicted binding sites in positive *D. melanogaster *pCRMs are roughly three times more likely to be aligned to predicted sites in the *D. pseudoobscura *compared to predicted binding sites in negative pCRMs, in the sequences flanking pCRMs, or in random regions of the genome. The demonstration that this strictest form of binding-site conservation is strengthened in functional CRMs contrasts with an earlier study that concluded that binding sites in functional CRMs had only a slightly elevated probability of falling in conserved sequence [32]. Their methodology differed from ours in that they used randomly shuffled binding-site positions within functional CRMs as the background, while we used actual predicted binding-site positions in randomly picked regions of the genome.

In addition to this colinear conservation, we also observe that there is an overall enrichment for binding sites in positive pCRMs independent of the conservation of individual sites. Specifically, the presence of a binding site for a factor in a positive *D. melanogaster *pCRM increases (relative to negative pCRMs and random genomic fragments) the probability of finding a site for the same factor in the orthologous region of *D. pseudoobscura*, even if the site is not in the same (aligned) position. Thus, in this set of positive pCRMs, there appears to be selection to maintain binding site composition, but not always the specific order and orientation of sites. This is consistent with models of enhancer plasticity that have been proposed and discussed elsewhere [25,59-61].

The relative importance of binding-site architecture and binding-site composition to maintaining the function of an enhancer over evolutionary time remains unclear. Over relatively short evolutionary distances (as between *D. melanogaster *and *D. pseudoobscura*) most binding sites are conserved and found in the same place. Over longer evolutionary distances, individual binding sites are often poorly conserved even as the overall composition and function of a CRM is conserved.

From a practical perspective, this requires adjusting how conservation is incorporated into searches for clusters of binding sites that are likely to be CRMs. For relatively short evolutionary distances, searches for clusters of aligned sites will be less sensitive to noise and will focus on functional binding sites. For longer distances, where binding site turnover will likely preclude searching for clusters of conserved sites, searches for conserved binding site clusters should still work well. In fact, this latter method can work - with some modification - among species whose sequences can no longer be aligned. *Anopheles gambiae *diverged from its common ancestor with *D. melanogaster *roughly 220 million years ago, and there is little or no detectable non-coding sequence similarity between these two species. Nonetheless, we find clusters of HB, KR and KNI binding sites in the vicinity of gap and pair-rule genes and suggest that many of these are functional orthologs of *D. melanogaster *CRMs. Despite strong selection to maintain function, enough binding-site turnover has occurred in these CRM during their 220 million years of independent evolution to eliminate detectable sequence similarity. But they remain functionally similar and we can detect this functional similarity through its evolutionary signature.

With methods like the one we have presented here, aided by new and better binding data on *Drosophila *transcription factors and an impending wealth of comparative sequence data, we anticipate rapid progress on the identification and functional characterization of regulatory sequences. We will then be able to turn our attention to the next great challenge - understanding the precise relationship between the binding-site composition and architecture of regulatory sequences and the expression patterns they specify.

## Materials and methods

### Collection of CRMs

The collection of CRM sequences was previously described [11]

### Transgenics

DNA fragments identified as candidate CRMs were amplified from either bacterial artifical chromosome (BAC) or *y; cn bw sp *fly genomic DNA by PCR using two primers containing unique sequence and synthetic *Asc*I and *Not*I restriction sites (Additional data file 5). The PCR product was digested with *Asc*I and *Not*I, and inserted in its native orientation into the *Asc*I-*Not*I site of a modified CaSpeR-AUG-bgal transformation vector [62] containing the *eve *basal promoter, starting at -42 bp and continuing through codon 22 fused in-frame with *lacZ *[63]. The P-element transformation vectors were injected into *w*^1118 ^embryos, as described previously [63,64]. Transgenic fly lines containing CRMs CE8005 (7A), CE8016 (55C) and CE8020 (70EF) were verified by generating genomic DNA [65] from each line for PCR. PCR products were amplified using primers designed from the CaSpeR-AUG-bgal vector - forward primer 5' CGCTTGGAGCTTCGTCAC and reverse primer 5' GAGTAACAACCCGTCGGATTC and 35 cycles (Gene Amp 9700, Perkin-Elmer). The resulting PCR products were sequenced using standard conditions with BigDye version 3.0 and electrophoresed on a 3730 capillary sequencer (ABI).

### Whole-mount *in situ *hybridizations

Embryonic whole-mount *in situ *RNA hybridizations were performed as previously described [21]. RNA probes were generated using cDNA clones RE29225 (*gt*), RE14252 (*odd*), RE34782 (*nub*), RE49429 (*pdm2*), and RE47384 (*sqz*). Exon 1 of the *ftz *gene was amplified from genomic DNA using forward primer 5' GCGTTGCGTGCACATC and reverse primer 5' ATTCTTCAGCTTCTGCGTCTG. The PCR product was cloned into the TA vector (Invitrogen) and used to generate *ftz *RNA probe.

### Double-labeling

RNA probes, using cDNAs or genomic DNA as templates, were labeled with fluorescein-12-UTP while *lacZ *RNA probes were labeled with digoxigenin-11-UTP (Roche). Hybridizations were performed as described above with the following modifications: (1) 2 μl of each probe were added to give a final concentration of 1:50; (2) sequential alkaline phosphatase staining was performed first with Sigma Fast red to detect endogenous transcripts, stopped by washing for 30 min in 0.1 M glycine-HCl pH 2.2, 0.1% Tween-20 at room temperature, and then continued as described to detect *lacZ *expression.

### Assembly

The input to the genome assembly was the set of whole-genome shotgun reads from the Baylor Genome Sequencing Center retrieved from the National Center for Biotechnology Information (NCBI) Trace Archive, consisting of 2,607,525 total sequences. After trimming the sequences to remove vector and low-quality regions, the average read length was 607 bp. Approximately 75% of the reads were from short insert (approximately 2.5-3.0 kb) libraries, with another 25% from longer (6-7 kb) libraries. Another 46,040 reads came from the ends of 40-kb fosmids.

We ran the Celera Assembler several times, and found that by adjusting one parameter in particular we could produce considerably better assemblies. In particular, the assembler has an arrival rate statistic *j*, which measures the probability that a contig is repetitive on the basis of its depth of coverage. The default setting is very conservative: if a contig has more than 50% likelihood of being repetitive, it is marked as such and is set aside during most of the assembly process. For large highly repetitive mammalian genomes this setting may be appropriate, but for *D. pseudoobscura *we found that setting it to 90% or higher produced considerably better contigs, while apparently causing few if any misassemblies.

The overall assembly contained 10,089 scaffolds and 10,329 contigs, containing 165,864,212 bp. The estimated span of the scaffolds, using the gap sizes estimated from clone insert sizes, is 172,362,884. The largest scaffold was 3.05 million base-pairs (Mbp) and the scaffold N50 size was 418,046. (The N50 size is the size of the smallest scaffold such that the total length of all scaffolds greater than this size is at least one half the total genome size, where genome size here is 172 Mbp.) There are 308 scaffolds larger than 100,000 bp, whose total span is 129.5 Mbp. The N50 contig size, using 166 Mbp as the genome size (not counting gaps), was 43,555. Another measure of assembly quality is the number of large contigs: if we define 'large' as 10 kbp, then the assembly contains 3177 large contigs whose total length is 131,067,828 bp. (For reference, the assembly produced by the Baylor Human Genome Sequencing Center contains 129.4 Mbp in all contigs, including small ones, and the span of all scaffolds is 139.3 Mbp.) All of our contigs and scaffolds are freely available by anonymous ftp at [66].

### Alignment and conservation of pCRMs

The extent and pattern of conservation between *D. melanogaster *and *D. pseudoobscura *in regions containing pCRMs were determined as follows. The *D. melanogaster *genomic sequence of the region of interest (with known repetitive elements masked) was extracted from a BioPerl genome database [67] containing Release 3.1 sequence and annotations from the Berkeley Drosophila Genome Project [68]. Potentially orthologous *D. pseudoobscura *contigs/scaffolds were identified using WU-BLAST 2.0 [69] using default parameters except for (-span1 -spsepqmax = 5000 -hspsepsmax = 5000 -gapsepmax = 5000 -gapsepsmax = 5000). High-scoring pairs (HSPs) with E-values less than 1e-20 were flagged as potential homologous regions. HSPs located more than 5,000 bp from each other in the *D. melanogaster *sequence were treated as separate hits. After examining dot-plots of the hits, we noticed a large number of small, local inversions that were found in both our assembly and the assemblies released by the Baylor Human Genome Sequencing Center. We used BLASTZ [70]) to automatically identify inversions, and when necessary inverted the corresponding *D. pseudoobscura *sequence. Each *D. pseudoobscura *sequence was aligned to the *D. melanogaster *corresponding sequence using LAGAN 1.2 [43] with default settings. A total of 31 genomic loci of approximately 50 kb were examined; these regions contain 36 pCRMs (the *eve *and *h *loci contain three pCRMs each, and PCE8003 and PCE8004 are within 20 kb of each other). Twenty-eight regions had aligned *D. pseudoobscura *sequence that spanned all or most of the region. For three regions (PCE8002, PCE8003/8004 and PCE8009) we were not able to identify large regions of orthologous sequence; these were excluded from subsequent comparative analyses. Dot-plots of the alignments from all 30 regions are available at [42].

### Scoring gross conservation of pCRMs

The conservation of a specific genomic segment was scored as the fraction of *D. melanogaster *bases aligned to the identical base in aligned regions (percent identity).

### Scoring binding-site conservation of pCRMs

We used two definitions of binding-site conservation. A binding site was considered 'aligned' if it overlaps a predicted *D. pseudoobscura *binding site for the same factor in the LAGAN alignment. Only overlap, and not strict alignment, was required to compensate for small errors in the alignment. A non-aligned binding site was considered 'preserved' if it could be matched to a *D. pseudoobscura *site for the same factor within the bounds of the pCRM, allowing each *D. pseudoobscura *site to be the match for only a single *D. melanogaster *site. The number of aligned plus preserved sites for each factor in a region is thus equal to the minimum number of sites for that factor in the two species.

### Generating an orthology map for genome searches

To develop an orthology map for genome-wide searches, we used NUCmer [71] to align the Release 3 *D. melanogaster *genome (with annotated repetitive elements and transposable elements masked) and the *D. pseudoobscura *scaffolds described above. NUCmer was run with the command line parameters (-c 36 -g 10 --mum -d 0.3 -l 9). NUCmer generated a collection of short, highly conserved regions of homology ('anchors') spaced on average every 1 kb throughout the *D. melanogaster *genome. Anchors flanking either side of a *D. melanogaster *region of interest were used to pull out the corresponding *D. pseudoobscura *region, and additional flanking anchors were examined to ensure that the region was unambiguously orthologous. The region identified was re-aligned to the melanogaster region with LAGAN 1.2 using default settings.

### Random sampling of non-coding genome

To characterize properties of non-coding sequences across the genome, we picked 4,000 1-kb segments of the *D. melanogaster *genome, sampled uniformly from all non-coding sequence. For 3,300 of these, we could find orthologous regions in *D. pseudoobscura*, and these were used to calculate the properties of random non-coding sequence shown in Figure 4 and discussed in the text. Properties determined using this data are considered properties of only the portion of the genome that is detectably orthologous under our conditions. The regions themselves are available as supplemental material at [42].

### eCIS-ANALYST genome searches

Binding-site clusters in the *D. melanogaster *genome were determined as described in [11], where the minimum number of sites (min_sites) and the window size (wind_size) are variable. Release 3 genomic sequence with exons masked was searched with PATSER [72] using the following command line options: -c -d2 -l4. An 'alphabet' file (specified with the command line parameter '-a') was used to provide the following background frequencies: A/T = 0.297, G/C = 0.203. Position weight matrix (PWM) models were identical to those used in [11]. In the online version of eCIS-ANALYST, the minimum PWM match threshold site_p is also variable, but in the current study it was held constant at 0.0003 for all factors. Tests using alternate values for this variable did not lead to significant improvement in prediction efficacy.

For each potential *D. melanogaster *cluster, we identified the corresponding *D. pseudoobscura *region using the homology anchors described above. A pairwise alignment was made using LAGAN 1.2 (default parameters), and the number of aligned and preserved binding sites were determined as described above. The 2-kb flanking either side of the pCRM was included in the alignment to avoid edge effects, and was subsequently removed when calculating pCRM properties.

We examined our functional (positive) and non-functional (negative) pCRMs and noticed that in the positives, the lower bound for the number of conserved sites as a function of *D. melanogaster *sites followed an approximately logarithmic curve (Additional data file 3). From this observation, we classified a *D. melanogaster *binding site cluster as conserved if:



where *NS*_*m *_is the number of binding sites in the *D. melanogaster *pCRM and *NS*_*c *_is the number of conserved binding sites. Different values of the logarithmic base *b *give different behavior. The data shown in Additional data file 3 support values of *b *between 1.15 and 1.4. We defined a more intuitive parameter, *CF *(conservation factor), which can range from 0 to 1 where 0 is the least stringent threshold (*b *= 1.4) and 1 is the most stringent (*b *= 1.15)

*b *= 1.4 - (CF * (1.4 - 1.15))     (2)

We performed genome searches with *CF *values of 0.25, 0.5, 0.55 and 0.75 and manually inspected the results with respect to false-negative and false-positive rates based on our 15 positive and 17 negative pCRMs (Additional data file 3). While we did not strictly optimize a single metric, we picked the values that gave a reasonable balance between false positives and false negatives, *b *= 0.25 for aligned sites alone, and *b *= 0.55 for aligned plus preserved sits.

### Genome-wide predictions

eCIS-ANALYST genome searches were run with the following parameters: min_sites = 10, wind_size = 700 (run #1), and min_sites = 13, wind_size = 1,100 (run #2). All conserved clusters (with conservation defined as described in Equations 1 and 2 above) were combined. In order to capture weaker clusters, we performed an additional run (run number 3) using min_sites = 9, wind_size = 700. For this low stringency run, we used a non-standard conservation threshold different from the one described above, accepting all clusters with at least four aligned plus preserved sites, independent of the number of sites in *D. melanogaster*. We merged overlapping clusters from runs 1-3, yielding 929 non-overlapping clusters as described in Results.

Four metrics were then used to rank these 929 pCRMs: the number of aligned binding sites; the density of aligned binding sites; the number of aligned plus preserved binding sites; and the density of aligned plus preserved binding sites. All values were normalized according to background distribution of random non-coding sequences. The four normalized values were then summed to compute an overall score, which was then renormalized to arrive at a final z-score used to rank pCRMs in Tables 3 and 4 and Additional data files 7, 8, 10, and 11.

## Additional data files

The following additional data files are available with the online version of this article.

Additional data file 1 shows the binding site densities (column 1), aligned site densities (column 2), and aligned plus preserved site densities (column 3) for individual transcription factors. The top portion of each panel contains a histogram of the values for randomly chosen 1,000 bp regions of the *D. melanogaster *genome. The blue line plots the cumulative distribution. The colored asterisks show the average values for each class of pCRM. The panel below the histogram shows the values for each pCRM (each dot represents one pCRM, with positives in blue, negatives in red, ambiguous in green).

Additional data file 2 shows expression patterns of 65 genes adjacent to 122 pCRMs identified by eCIS-ANALYST. The images were obtained from the BDGP Embryonic Expression Pattern Database [33], and include all pCRMs from Additional data files 7,8,10,11 for which an adjacent gene had an early segmentation pattern.

Additional data file 3 shows discrimination of positive and negative pCRMs. Comparisons of the number of predicted binding sites in *D. melanogaster *pCRMs to the number of aligned sites (top panel) and aligned plus preserved sites (bottom panel). Blue dots represent the 15 positive pCRMs from the text; green dots the ten known CRMs that were below the threshold used in [11]; red dots negative pCRMs; pink dots ambiguous pCRMs. Gray boxes represent the distribution of values for random 1,000 bp non-coding regions. The blue line shows the discrimination function (see Materials and methods).

Additional data file 4 shows new pCRMs. Three 30 kb regions were chosen to illustrate new predictions: (A) the argos locus, (B) the CG4702 locus (note that CG31361 is not expressed in blastoderm embryos and PCE8494 is a low-scoring pCRM), and (C) the SoxN locus. Exons are shows as blue boxes, introns are represented with horizontal lines, and the direction of transcription is indicated by the arrow. New pCRMs are shown as gray ovals. The green graphs show average (in 300 bp windows) percent identity and fraction of bases in conserved blocks. Below the percent identity plots are shown insertions (gray boxes) and deletions (orange boxes) in the *D. melanogaster *sequence relative to their *D. pseudoobscura *ortholog. The location of binding sites in *D. melanogaster*, binding sites in *D. pseudoobscura *and aligned binding sites along with the density of sites averaged over 700 bp are shown in the bottom three panels for each region.

Additional data file 5 gives the primers used to amplify pCRMs for transgenics. Additional data file 6 gives additional information from Table 2. Additional data file 7 gives all new pCRMs from genome-wide eCIS-ANALYST located within 20 kb of annotated transcript. Additional data file 8 gives all new pCRMs from genome-wide eCIS-ANALYST located more than 20 kb from annotated transcript. Additional data file 9 lists genes with anterior-posterior patterns and the source of the information. Additional data file 10 gives all new pCRMs from genome-wide eCIS-ANALYST located within 20 kb of gene with anterior-posterior pattern. And, finally, Additional data file 11 gives all new pCRMs from genome-wide eCIS-ANALYST located between 20 kb and 50 kb from gene with anterior-posterior pattern.

## Supplementary Material

Additional data file 1 The binding site densities (column 1), aligned site densities (column 2), and aligned plus preserved site densities (column 3) for individual transcription factors Click here for additional data file

Additional data file 2 Expression patterns of 65 genes adjacent to 122 pCRMs identified by eCIS-ANALYST Click here for additional data file

Additional data file 3 Discrimination of positive and negative pCRMs. Comparisons of the number of predicted binding sites in *D. melanogaster *pCRMs to the number of aligned sites (top panel) and aligned plus preserved sites (bottom panel) Click here for additional data file

Additional data file 4 New pCRMs Click here for additional data file

Additional data file 5 The primers used to amplify pCRMs for transgenics Click here for additional data file

Additional data file 6 Additional information from Table 2 Click here for additional data file

Additional data file 7 All new pCRMs from genome-wide eCIS-ANALYST located within 20 kb of annotated transcript Click here for additional data file

Additional data file 8 All new pCRMs from genome-wide eCIS-ANALYST located more than 20 kb from annotated transcript Click here for additional data file

Additional data file 9 Genes with anterior-posterior patterns and the source of the information Click here for additional data file

Additional data file 10 All new pCRMs from genome-wide eCIS-ANALYST located within 20 kb of gene with anterior-posterior pattern Click here for additional data file

Additional data file 11 All new pCRMs from genome-wide eCIS-ANALYST located between 20 kb and 50 kb from gene with anterior-posterior pattern Click here for additional data file

## Figures and Tables

**Table 1:** Genomic location of pCRMs and neighboring genes

	pCRM	ID*	Name	CRM activity	Arm	pCRM start	pCRM end	pCRM length	5' gene	pCRM relative position	3' gene	pCRM relative position
1	PCE7001		runt stripe 3	+	X	20,357,206	20,358,294	1,089	CG1338	-9,550	**run**	-8,561
2	PCE7002		eve stripes 3/7	+	2R	5,035,494	5,036,771	1,278	CG12134	3,713	**eve**	-2,952
3	PCE7003		eve stripe 2	+	2R	5,038,454	5,039,040	587	CG12134	6,673	**eve**	-683
4	PCE7004		eve stripes 4/6	+	2R	5,044,597	5,045,395	799	**eve**	4,874	TER94	-4,398
5	PCE7005		hairy stripe 7	+	3L	8,624,351	8,625,245	895	CG6486	16,118	**h**	-9,423
6	PCE7006		hairy stripe 6	+	3L	8,625,452	8,626,319	868	CG6486	17,219	**h**	-8,349
7	PCE7007		hairy stripes 1,5	+	3L	8,629,180	8,629,966	787	CG6486	20,947	**h**	-4,702
8	PCE7008		kni upstream	+	3L	20,615,070	20,616,425	1,356	**kni**	-1,169	CG13253	21,311
9	PCE7009		hb HZ1.4	+	3R	4,526,315	4,527,521	1,207	**hb**	-2,760	CG8112	403

												
10	PCE8001	1	gt posterior domain	+	X	2,187,439	2,188,382	944	**gt**	-1,704	tko	12,366
11	PCE8010	2	odd stripes 3/6	+	2L	3,601,750	3,602,509	760	**odd**	-2,433	Dot	-9,351
12	PCE8011	3	nub blastoderm	+	2L	12,605,345	12,606,039	695	CG15488	2,687	**nub**	-1,178
13	PCE8024	4	ftz stripes 1/5	+	3R	2,693,713	2,694,405	693	**ftz**	3,667	**Antp**	131,873
14	PCE8012	5	pdm2 neurogenic	+	2L	12,663,878	12,664,600	723	***pdm2***	2,875	***pdm2***	2,875
15	PCE8027	6	sqz neurogenic	+	3R	15,000,096	15,000,905	810	sqz	10,137	CG14282	-1,833

												
16	PCE8005	7	cluster_at_7A	amb.	X	6,996,209	6,996,756	548	CG32725	-17,671	CG1958	-10,524
17	PCE8016	8	cluster_at_55C	amb.	2R	13,354,407	13,355,109	703	*CG14502*	957	*CG14502*	957
18	PCE8020	9	cluster_at_70F	amb.	3L	14,665,967	14,666,676	710	*ome*	10,334	*ome*	10,334

												
19	PCE8006	13	cluster_at_7B	-	X	7,239,486	7,240,124	639	CG11368	46,902	CG32719	13,096
20	PCE8008	15	cluster_at_8F	-	X	9,457,631	9,458,375	745	**btd**	24,460	**Sp1**	-33,567
21	PCE8013	17	cluster_at_34E	-	2L	13,989,283	13,990,132	850	rk	-5,879	bgm	-5,767
22	PCE8014	18	cluster_at_36F	-	2L	18,400,758	18,401,458	701	CG31749	36,362	RpS26	19,862
23	PCE8015	19	cluster_at_47A	-	2R	5,664,440	5,665,094	655	***psq***	45,904	***psq***	45,904
24	PCE8017	20	cluster_at_56B	-	2R	14,266,629	14,267,261	633	*CG7097*	24,156	*CG7097*	24,156
25	PCE8018	21	cluster_at_59B	-	2R	17,995,894	17,996,609	716	*CG32835*	759	*CG32835*	759
26	PCE8019	22	cluster_at_67B	-	3L	9,529,913	9,530,579	667	*CG32048*	10,499	*CG32048*	10,499
27	PCE8021	23	cluster_at_75C	-	3L	18,339,914	18,340,665	752	**grim**	-86,621	**rpr**	6,617
28	PCE8022	24	cluster_at_76C	-	3L	19,594,180	19,594,883	704	CG8786	-1,409	CG8782	4,923
29	PCE8023	25	cluster_at_84A	-	3R	2,595,162	2,595,926	765	**Ama**	6,847	**Dfd**	-21,632
30	PCE8025	26	cluster_at_85C	-	3R	4,944,607	4,945,444	838	*pum*	117,315	*pum*	117,315
31	PCE8026	27	cluster_at_88F	-	3R	11,424,315	11,424,996	682	CG18516	-45,803	CG5302	-33,626
32	PCE8028	28	cluster_at_95C	-	3R	19,757,908	19,758,531	624	*Gdh*	950	*Gdh*	950

												
33	PCE8003	11	cluster_at_5C.1	-	X	5,658,504	5,659,131	628	*CG3726*	952	*CG3726*	952
34	PCE8004	12	cluster_at_5C.2	-	X	5,674,913	5,675,606	694	*CG3726*	17,361	*CG3726*	17,361
35	PCE8009	16	cluster_at_12E	-	X	14,146,556	14,147,218	663	*CG32600*	93,317	*CG32600*	93,317
36	PCE8002	10	cluster_at_4B	-	X	4,124,119	4,125,459	1,341	CG12688	2,032	CG32773	3,408
37	PCE8007	14	cluster_at_7F	Unknown	X	8,350,658	8,351,315	658	Caf1-180	-5,486	**oc**	38,281

*IDs in this column are taken from [11]. Genomic locations of the 37 pCRMs identified in our previous genome search. All coordinates are from *D. melanogaster *Release 3 [68]. pCRMs 1-9 were reported prior to our original search, and we attempted to characterize 10-37 in the current study (we reported PCE8001 in our previous publication). pCRMs10-15 recapitulate endogenous expression patterns of embryonic genes, and 16-18 drive ambiguous (amb.) expression patterns, as described in the text. pCRMs 19-36 drove no detectable expression in the embryo, and pCRM 37 was not tested. Orthologous regions were identified in *D. pseudoobscura *for all but pCRMs 33-37. The 5' and 3' gene columns correspond to the closest transcription (or annotation) start 5' and 3' of the pCRM. If a pCRM is within an intron, only the intron-containing gene is reported and its name is given in italics. The names of genes with early anterior-posterior patterns are in bold.

**Table 2:** Sequence and binding-site conservation in pCRMs between *D. melanogaster *and *D. pseudoobscura*

	pCRM	Name	CRM activity	pCRM length (*D. melanogaster*)	pCRM length (*D. pseudoobscura*)	Percent identity	*D. melanogaster *sites	*D. pseudoobscura *sites	Conserved sites		Fraction conserved	
									
									A	A+P	A	A+P
1	PCE7001	runt stripe 3	+	1,089	1,504	71%	27	20	11	20	41%	74%
2	PCE7002	eve stripes 3/7	+	1,278	1,114	61%	28	25	21	25	75%	89%
3	PCE7003	eve stripe 2	+	587	771	67%	14	10	9	10	64%	71%
4	PCE7004	eve stripes 4/6	+	799	1,003	70%	20	18	13	17	65%	85%
5	PCE7005	hairy stripe 7	+	895	869	66%	20	16	12	16	60%	80%
6	PCE7006	hairy stripe 6	+	868	952	62%	23	19	11	19	48%	83%
7	PCE7007	hairy stripes 1,5	+	787	723	56%	16	15	9	13	56%	81%
8	PCE7008	kni upstream	+	1,356	1,654	68%	33	31	24	30	73%	91%
9	PCE7009	hb HZ1.4	+	1,207	1,383	69%	24	23	17	21	71%	88%

												
10	PCE8001	gt posterior domain	+	944	1,092	64%	23	19	15	18	65%	78%
11	PCE8010	odd stripes 3/6	+	760	825	70%	17	19	12	16	71%	94%
12	PCE8011	nub blastoderm	+	695	894	70%	18	13	10	12	56%	67%
13	PCE8024	ftz stripes 1/5	+	693	744	77%	14	10	10	10	71%	71%
14	PCE8012	pdm2 neurogenic	+	723	723	72%	14	8	4	8	29%	57%
15	PCE8027	sqz neurogenic	+	810	818	69%	16	17	11	14	69%	88%

												
16	PCE8005	cluster_at_7A	amb.	548	819	54%	13	4	2	2	15%	15%
17	PCE8016	cluster_at_55C	amb.	703	1,617	55%	16	6	3	6	19%	38%
18	PCE8020	cluster_at_70F	amb.	710	538	47%	14	2	2	2	14%	14%

												
19	PCE8006	cluster_at_7B	-	639	663	69%	15	9	8	8	53%	53%
20	PCE8008	cluster_at_8F	-	745	716	58%	14	2	1	2	7%	14%
21	PCE8013	cluster_at_34E	-	850	919	61%	17	8	6	8	35%	47%
22	PCE8014	cluster_at_36F	-	701	596	53%	15	6	5	6	33%	40%
23	PCE8015	cluster_at_47A	-	655	652	66%	16	3	3	3	19%	19%
24	PCE8017	cluster_at_56B	-	633	331	33%	15	9	4	8	27%	53%
25	PCE8018	cluster_at_59B	-	716	960	59%	16	4	3	4	19%	25%
26	PCE8019	cluster_at_67B	-	667	675	62%	15	7	5	6	33%	40%
27	PCE8021	cluster_at_75C	-	752	640	59%	19	13	10	12	53%	63%
28	PCE8022	cluster_at_76C	-	704	725	67%	15	9	7	9	47%	60%
29	PCE8023	cluster_at_84A	-	765	1,001	55%	16	7	5	7	31%	44%
30	PCE8025	cluster_at_85C	-	838	827	54%	16	6	1	5	6%	31%
31	PCE8026	cluster_at_88F	-	682	1,096	62%	16	6	5	5	31%	31%
32	PCE8028	cluster_at_95C	-	624	723	60%	15	6	4	6	27%	40%

												
33	PCE8003	cluster_at_5C.1	-	628	None		15					
34	PCE8004	cluster_at_5C.2	-	694	None		15					
35	PCE8009	cluster_at_12E	-	663	None		15					
36	PCE8002	cluster_at_4B	-	1,341	None		28					
37	PCE8007	cluster_at_7F	Unknown	658	None		15					

												
	Mean (pCRMs 1-15)			899	1,005	67%	20	18	13	17	61%	80%
	Mean (pCRMs 19-32)			712	752	58%	16	7	5	6	30%	40%

Conservation properties are listed for the pCRMs described in Table 1. The number and fraction of conserved sites are shown under two conditions - aligned sites only (A), or aligned + preserved sites (A+P) (see Materials and methods). *D. pseudoobscura *sequences used to determine these properties are available as supplemental material at [42].

**Table 3:** New pCRMs from genome-wide eCIS-ANALYST (75 highest scoring predictions)

	CRM	Known element overlap	Arm	pCRM start	pCRM end	pCRM length	5' gene	pCRM relative position	3' gene	pCRM relative position	Conserved sites		Conserved site density		z score	Additional gap/pair-rule gene within 20 kb	pCRM relative position
											A	A+P	A	A+P			
1	PCE8050	h stripes 3/4,6,7 [73]	3L	8,622,879	8,626,839	3,961	CG6486	+14646	**h**	-7829	36	62	9	16	20.1		
2	PCE8051	kni upstream [74]	3L	20,614,714	20,617,020	2,307	**kni**	-813	CG13253	+20716	25	31	11	13	13.2		
3	PCE8052	nub blastoderm	2L	12,604,311	12,606,913	2,603	CG15488	+1653	**nub**	-304	20	33	8	13	11.6		
4	PCE8053	eve stripes 3/7 [75]	2R	5,035,493	5,037,290	1,798	CG12134	+3712	**eve**	-2433	21	24	12	13	11.5	**Adam**	+5901
5	PCE8054	hairy stripes 1,5 [73]	3L	8,628,846	8,631,011	2,166	CG6486	+20613	**h**	-3657	17	29	8	13	10.5		
6	PCE8055	runt stripe 3 [76]	X	20,356,848	20,360,054	3,207	CG1338	-9192	**run**	-6801	17	34	5	11	10.3		
7	PCE8056		X	20,323,964	20,326,397	2,434	CG11692	-12536	Cyp6v1	-4186	16	28	7	12	9.6		
8	PCE8057	hb HZ1.4 [77]	3R	4,526,225	4,527,991	1,767	**hb**	-2670	*CG8112*	+1273	17	21	10	12	9.5		
9	PCE8059	eve stripes 4/6 [78]	2R	5,044,597	5,046,030	1,434	**eve**	+4874	TER94	-3763	15	18	10	13	9.0	**Adam**	+15005
10	PCE8060	gt posterior [11]	X	2,186,709	2,189,069	2,361	**gt**	-974	tko	+11679	18	21	8	9	8.9		
11	PCE8061		X	3,169,806	3,172,348	2,543	CG12535	-17954	CG14269	+21857	13	29	5	11	8.8		
12	PCE8063	CE8021	3L	18,339,914	18,341,941	2,028	**grim**	-86621	rpr	+5341	16	20	8	10	8.5		
13	PCE8064		3R	6,255,663	6,256,945	1,283	CG6345	-13879	Cyp12e1	-3594	13	17	10	13	8.4		
14	PCE8065		3R	4,026,032	4,027,816	1,785	**grn**	-18853	CG7800	-15898	15	19	8	11	8.4		
15	PCE8066		X	20,348,460	20,352,624	4,165	CG1338	-804	**run**	-14231	16	28	4	7	8.3		
16	PCE8067	ftz upstream [23]	3R	2,682,314	2,684,591	2,278	**Scr**	-7972	**ftz**	-5455	15	22	7	10	8.3		
17	PCE8068		X	18,701,007	18,702,700	1,694	*CG32541*	+39691	*CG32541*	+39691	12	22	7	13	8.2		
18	PCE8069		2R	17,274,311	17,276,017	1,707	CG3380	-2521	dve	-11496	14	19	8	11	8.2		
19	PCE8070		2L	7,616,050	7,618,366	2,317	CG6739	+15430	CG13792	+19862	14	23	6	10	8.1		
20	PCE8071	sqz neurogenic	3R	14,999,463	15,001,552	2,090	sqz	+9504	CG14282	-1186	12	24	6	11	8.0	**nos**	+16485
21	PCE8072		X	5,674,422	5,676,386	1,965	*CG3726*	+16870	CG12728	-6597	11	24	6	12	7.8		
22	PCE8073		2R	14,903,099	14,903,925	827	Toll-7	+12482	Obp56i	-27903	11	11	13	13	7.8		
23	PCE8074		3R	23,192,304	23,192,750	447	CG13980	+8073	*side*	+40862	7	8	16	18	7.7		
24	PCE8075		3R	10,762,920	10,764,750	1,831	CG3837	+18501	CG14861	-75759	13	19	7	10	7.6		
25	PCE8076	eve stripe 2 [75]	2R	5,038,454	5,039,041	588	CG12134	+6673	**eve**	-682	8	10	14	17	7.6	**Adam**	+8862
26	PCE8077		2L	13,541,662	13,542,651	990	*kuz*	+9371	*kuz*	+9371	11	13	11	13	7.6		
27	PCE8078		2L	14,424,056	14,425,158	1,103	BG:DS06238.4	-16773	BG:DS08340.1	+7810	12	13	11	12	7.6		
28	PCE8080	odd stripes 3/6	2L	3,601,045	3,602,748	1,704	**odd**	-1728	Dot	-9112	12	19	7	11	7.5		
29	PCE8081		3L	17,412,324	17,413,414	1,091	CG18265	+24035	CG7603	-1413	11	14	10	13	7.5		
30	PCE8083		3L	14,121,556	14,123,127	1,572	Sox21b	-41352	**D**	+4373	12	17	8	11	7.3		
31	PCE8084		2L	4,098,489	4,099,006	518	*ed*	+74542	*ed*	+74542	7	9	14	17	7.3		
32	PCE8085		2R	12,253,766	12,255,302	1,537	CG10953	-23540	CG10950	-3625	13	15	8	10	7.2		
33	PCE8086		3L	20,612,647	20,614,073	1,427	***kni***	+1254	CG13253	+23663	11	17	8	12	7.2		
34	PCE8087		2R	3,391,037	3,391,561	525	CG30358	+10444	CG14755	-16724	7	9	13	17	7.2		
35	PCE8088		3L	16,418,107	16,418,469	363	*CG33158*	+49435	**argos**	+14111	6	6	17	17	7.2		
36	PCE8089		3R	12,368,159	12,368,687	529	CG11769	+28970	CG31448	-670	7	9	13	17	7.2	**CG14889**	-13735
37	PCE8091		3L	11,213,064	11,213,664	601	scylla	+3224	CG32083	+24695	8	9	13	15	7.1		
38	PCE8092		2L	1,233,357	1,235,228	1,872	CG5156	+3715	CG5397	-6475	9	23	5	12	7.1		
39	PCE8093		3L	15,688,222	15,691,204	2,983	**comm**	-10920	CG13445	-67172	13	22	4	7	7.0		
40	PCE8094		2R	10,492,861	10,493,546	686	CG30472	-5321	CG12959	-26488	9	9	13	13	7.0		
41	PCE8095		3R	23,894,562	23,895,459	898	*CG12870*	+31901	*CG12870*	+31901	10	11	11	12	7.0		
42	PCE8096		3L	6,762,543	6,765,157	2,615	vvl	+12855	Prat2	+108336	13	20	5	8	6.9		
43	PCE8097		3R	10,238,130	10,238,652	523	CG14846	-1983	CG14847	+4557	7	8	13	15	6.8		
44	PCE8099		2L	18,305,051	18,306,251	1,201	*Fas3*	+6868	*Fas3*	+6868	10	14	8	12	6.7		
45	PCE8100	eve early APR [79]	2R	5,042,174	5,042,884	711	**eve**	+2451	TER94	-6909	8	10	11	14	6.7	**Adam**	+12582
46	PCE8102	tll posterior [80]	3R	26,663,942	26,665,204	1,263	CG15544	+21005	**tll**	-2251	11	13	9	10	6.6		
47	PCE8104	ems neurogenic [81]	3R	9,723,602	9,724,936	1,335	E5	-23682	**ems**	-2663	12	12	9	9	6.6		
48	PCE8105		3R	17,817,909	17,818,791	883	*Eip93F*	+25598	*Eip93F*	+25598	9	11	10	12	6.6		
49	PCE8106		3L	10,499,018	10,501,551	2,534	*CG32062*	+25485	*CG32062*	+25485	11	21	4	8	6.6		
50	PCE8107		3L	4,612,891	4,614,005	1,115	CG13716	-161	CG13715	+1681	11	11	10	10	6.6		
51	PCE8108		2L	14,403,771	14,404,937	1,167	CG15284	-4301	BG:DS06238.4	+2346	10	13	9	11	6.5		
52	PCE8109		3R	7,941,601	7,942,426	826	CG31361	+17775	**CG4702**	+11512	9	10	11	12	6.5		
53	PCE8110		2L	8,804,166	8,805,336	1,171	CG9468	-30684	**SoxN**	-12519	10	13	9	11	6.5		
54	PCE8111		3L	8,612,337	8,613,016	680	CG6486	+4104	**h**	-21652	8	9	12	13	6.5		
55	PCE8112		3L	4,377,989	4,379,208	1,220	*CG7447*	+13842	Syx17	-3984	11	12	9	10	6.5		
56	PCE8113		2L	14,113,291	14,113,893	603	CG15292	-3974	CG31768	-6693	7	9	12	15	6.5		
57	PCE8114		3L	3,997,600	3,998,923	1,324	CG14985	+13500	fd64A	-799	11	13	8	10	6.5		
58	PCE8115	eve stripe 1 [79]	2R	5,046,559	5,047,297	739	**eve**	+6836	TER94	-2496	8	10	11	14	6.5	**Adam**	+16967
59	PCE8116		2R	16,921,501	16,922,240	740	CG13493	-11091	PpN58A	+4194	8	10	11	14	6.5		
60	PCE8118		3R	14,822,848	14,823,484	637	***gukh***	+13085	***gukh***	+13085	8	8	13	13	6.4		
61	PCE8119		3R	12,671,525	12,672,987	1,463	**abd-A**	-15737	CG10349	-32477	11	14	8	10	6.4		
62	PCE8120		3L	10,492,688	10,495,539	2,852	*CG32062*	+19155	*CG32062*	+19155	10	23	4	8	6.4		
63	PCE8121		2L	16,841,696	16,842,392	697	CG6012	-2193	CG31781	-5178	8	9	11	13	6.4		
64	PCE8122		3L	6,885,832	6,887,436	1,605	Prat2	-11445	CG14820	-5022	11	15	7	9	6.4		
65	PCE8123		2L	15,162,778	15,164,524	1,747	BG:DS03192.2	-6373	BG:DS07295.1	+59479	11	16	6	9	6.4		
66	PCE8124		2R	6,888,483	6,889,700	1,218	CG12443	+13963	CG13192	-428	10	13	8	11	6.4		
67	PCE8125		2L	20,466,022	20,467,708	1,687	CG2493	-32831	CG15476	+4184	10	17	6	10	6.4		
68	PCE8126		3L	2,779,198	2,779,658	461	*CG2083*	+1101	*CG2083*	+1101	6	7	13	15	6.3		
69	PCE8127		X	4,630,473	4,632,106	1,634	CG12681	+14179	CG15470	-3196	9	18	6	11	6.3		
70	PCE8128		3R	27,713,381	27,715,087	1,707	*heph*	+35171	*heph*	+35171	10	17	6	10	6.3		
71	PCE8130		3R	12,383,752	12,385,269	1,518	***CG14889***	+1858	***CG14889***	+1858	11	14	7	9	6.3		
72	PCE8131		3R	21,329,716	21,331,058	1,343	CG5111	+8355	msi	-2351	8	17	6	13	6.3		
73	PCE8132		3R	16,242,660	16,243,128	469	CG10881	+8657	CG17208	+20535	6	7	13	15	6.3		
74	PCE8133		3R	24,120,296	24,122,240	1,945	CG12516	-668	larp	+19112	12	15	6	8	6.2		
75	PCE8134		3L	8,733,754	8,734,394	641	*CG32030*	+8601	*CG32030*	+8601	7	9	11	14	6.2		

Seventy-five top pCRMs, ranked by a z-score based on the number and density of conserved binding sites (see text for details). Site density columns list the number of conserved sites per kilobase (relative to the *D. melanogaster *sequence). The number and density of conserved sites are shown under two conditions - aligned sites only (A), or aligned + preserved sites (A+P) (see Materials and methods). The 5' and 3' gene columns correspond to the closest transcription (or annotation) start 5' and 3' of the pCRM. If a pCRM is within an intron, only the intron-containing gene is reported and its name is italicized. The names of genes with early anterior-posterior patterns are in bold. Early anterior-posterior genes that start within 20 kb of the pCRM (but are not the immediate annotation in the 5' or 3' direction) are also listed. Named enhancers without a reference are from this study.

**Table 4:** Additional new pCRMs within 20 kb of genes with anterior-posterior patterns

	CRM	Known element overlap	Arm	pCRM start	pCRM end	pCRM length	5' gene	pCRM relative position	3' gene	pCRM relative position	Conserved sites		Conserved site density		z score	Additional Gap/pair-rule gene within 20 kb	pCRM relative position
														
											A	A+P	A	A+P			
1	PCE8137		3R	12,053,627	12,055,472	1,846	***tara***	+2239	***tara***	+2239	10	17	5	9	6.1		
2	PCE8139		2R	6,573,169	6,574,383	1,215	inv	+32752	CG30034	+12378	10	12	8	10	6.1	**en**	+19407
3	PCE8140		2R	15,167,055	15,168,270	1,216	CG16898	-98356	18w	-6952	10	12	8	10	6.1		
4	PCE8144		3L	3,503,831	3,504,156	326	*Eip63E*	+7518	*Eip63E*	+7518	4	6	12	18	6.1	**ImpE2**	-10525
5	PCE8145		3R	4,536,237	4,536,936	700	*CG8112*	+1795	*CG8112*	+1795	8	8	11	11	6.0	**hb**	-12682
6	PCE8150		3R	6,379,567	6,380,474	908	***hth***	+50936	***hth***	+50936	8	11	9	12	6.0		
7	PCE8165		X	8,390,109	8,392,075	1,967	**oc**	-513	CG12772	-23984	10	16	5	8	5.8		
8	PCE8166		3R	12,570,467	12,571,123	657	**Ubx**	-10101	CG31275	+5951	7	8	11	12	5.7		
9	PCE8167	Ubx S1 [82]	3R	12,589,099	12,589,755	657	**CG31275 (Ubx adjacent)**	-11970	Glut3	-24295	7	8	11	12	5.7		
10	PCE8169	ftz stripes 1/5 [51]	3R	2,693,336	2,694,915	1,580	**ftz**	+3290	**Antp**	+63624	11	12	7	8	5.7		
11	PCE8170		3R	2,670,658	2,672,242	1,585	***Scr***	+2100	***Scr***	+2100	9	15	6	9	5.7	**ftz**	-19388
12	PCE8177		2R	5,634,520	5,635,604	1,085	***psq***	+4661	***psq***	+4661	8	12	7	11	5.7		
13	PCE8183		2L	7,305,525	7,305,940	416	***wg***	+4205	***wg***	+4205	5	6	12	14	5.6		
14	PCE8187		2L	8,286,022	8,287,399	1,378	***Btk29A***	+5904	***Btk29A***	+5904	9	13	7	9	5.6		
15	PCE8190		3L	6,589,453	6,590,721	1,269	***Glu-RI***	+5891	***Glu-RI***	+5891	9	12	7	9	5.6		
16	PCE8193	Kr CD2 [83]	2R	20,268,656	20,269,940	1,285	CG9380	-36249	**Kr**	-244	7	15	5	12	5.5		
17	PCE8195		3L	5,126,445	5,126,805	361	***CG32423***	+17297	***CG32423***	+17297	4	6	11	17	5.5		
18	PCE8198		2L	3,767,311	3,769,396	2,086	***bowl***	+2110	***bowl***	+2110	9	17	4	8	5.5		
19	PCE8210		3L	7,925,371	7,926,049	679	**exex**	+17651	RNaseX25	-4074	6	9	9	13	5.4		
20	PCE8214		2L	12,601,146	12,602,225	1,080	ref2	-895	CG15488	-433	8	11	7	10	5.4	**nub**	-6071
21	PCE8218		2L	10,545,226	10,547,197	1,972	***CG31721***	+7937	***CG31721***	+7937	10	14	5	7	5.3		
22	PCE8226		2L	12,541,433	12,542,145	713	**bun**	-11992	CG15489	-40512	6	9	8	13	5.2		
23	PCE8235		X	2,190,216	2,191,697	1,482	**gt**	-4481	tko	+9051	9	12	6	8	5.2		
24	PCE8237		2L	12,670,755	12,671,417	663	***pdm2***	+3280	***pdm2***	+3280	6	8	9	12	5.2		
25	PCE8258		3L	15,491,385	15,492,925	1,541	***CrebA***	+7093	***CrebA***	+7093	7	15	5	10	5.1		
26	PCE8270		3L	16,421,730	16,422,846	1,117	***argos***	+9734	***argos***	+9734	8	10	7	9	5.0		
27	PCE8275		3L	18,329,419	18,330,261	843	**grim**	-76126	**rpr**	+17021	6	10	7	12	5.0		
28	PCE8277		3R	6,448,750	6,449,993	1,244	***hth***	+8759	***hth***	+8759	6	14	5	11	5.0		
29	PCE8297		2R	20,280,374	20,281,018	645	**Kr**	+10190	CG30429	-9080	6	7	9	11	4.9		
30	PCE8306		3L	12,278,550	12,279,346	797	CG4328	-28041	**CG32105**	-7436	6	9	8	11	4.9		
31	PCE8307		3L	5,580,997	5,581,649	653	CG12756	-13449	**CG5249**	-8641	6	7	9	11	4.9		
32	PCE8309		2L	3,825,809	3,827,419	1,611	**slp1**	+7561	**slp2**	-1991	8	13	5	8	4.9		
33	PCE8314		2L	3,842,537	3,843,621	1,085	**slp2**	+13127	CG3964	-11628	6	12	6	11	4.8		
34	PCE8328		2L	16,418,533	16,419,580	1,048	***BG:DS02780.1***	+8016	Idgf1	-3783	7	10	7	10	4.8		
35	PCE8331		3L	5,582,709	5,583,340	632	CG12756	-15161	**CG5249**	-6950	5	8	8	13	4.8		
36	PCE8332		3R	2,725,376	2,726,195	820	***Antp***	+32344	***Antp***	+32344	6	9	7	11	4.8		
37	PCE8338		3R	3,987,824	3,989,532	1,709	***grn***	+17647	***grn***	+17647	8	13	5	8	4.7		
38	PCE8348		3L	18,966,181	18,967,380	1,200	***nkd***	+26830	***nkd***	+26830	7	11	6	9	4.7		
39	PCE8355		3R	6,421,647	6,422,583	937	***hth***	+8827	***hth***	+8827	6	10	6	11	4.7		
40	PCE8356		3L	22,244,275	22,244,894	620	***Ten-m***	+80890	CG32450	-2161	6	6	10	10	4.7		
41	PCE8358		3R	26,740,914	26,742,495	1,582	***Ptx1***	+2496	***Ptx1***	+2496	8	12	5	8	4.7		
42	PCE8361	Ubx BRE [84]	3R	12,526,665	12,527,949	1,285	***Ubx***	+32417	***Ubx***	+32417	6	13	5	10	4.6		
43	PCE8367		2R	4,771,288	4,771,881	594	CG10459	+3018	**dap**	-1074	5	7	8	12	4.6		
44	PCE8369		3L	14,540,753	14,541,382	630	***HGTX***	+7066	***HGTX***	+7066	6	6	10	10	4.6		
45	PCE8370		3L	2,395,158	2,396,393	1,236	CG13800	+12412	**CG32306**	-13538	5	14	4	11	4.6		
46	PCE8391		3L	5,254,002	5,254,895	894	**CG32423**	-16750	lama	+55892	6	9	7	10	4.5		
47	PCE8394	Kr 730 [83]	2R	20,266,323	20,267,047	725	CG9380	-33916	**Kr**	-3137	6	7	8	10	4.5		
48	PCE8398		3R	2,770,846	2,771,901	1,056	***Antp***	+12307	***Antp***	+12307	7	9	7	9	4.5		
49	PCE8401		2L	12,660,502	12,661,614	1,113	CG15485	-2463	***pdm2***	+5861	6	11	5	10	4.5		
50	PCE8408		X	8,379,690	8,381,014	1,325	***oc***	+8582	***oc***	+8582	5	14	4	11	4.4		
51	PCE8415		3R	13,867,601	13,868,164	564	CG7794	+18158	**htl**	+6934	5	6	9	11	4.4		
52	PCE8417		2L	587,804	588,638	835	***Gsc***	+7714	***Gsc***	+7714	6	8	7	10	4.4		
53	PCE8418		3R	18,950,000	18,950,634	635	CG31457	-5638	***hh***	+7739	5	7	8	11	4.4	**cenB1A**	12397
54	PCE8425		2R	18,693,096	18,694,318	1,223	***retn***	+16917	CG5411	-6825	7	10	6	8	4.4		
55	PCE8439		X	4,770,587	4,771,859	1,273	CG12680	+32240	ovo	-17051	7	10	5	8	4.3		
56	PCE8444		3L	18,330,763	18,332,045	1,283	**grim**	-77470	rpr	+15237	7	10	5	8	4.3		
57	PCE8450		3L	5,141,131	5,141,793	663	***CG32423***	+2971	CG10677	-438	5	7	8	11	4.3		
58	PCE8458		3L	19,101,833	19,102,666	834	***fz2***	+6194	***fz2***	+6194	5	9	6	11	4.2		
59	PCE8464		3L	17,314,105	17,314,815	711	**tap**	+5577	**Cad74A**	+13577	6	6	8	8	4.2		
60	PCE8483		2L	8,265,854	8,267,283	1,430	***Btk29A***	+2646	***Btk29A***	+2646	4	15	3	10	4.1		
61	PCE8493		3R	6,403,852	6,405,604	1,753	***hth***	+25806	***hth***	+25806	7	12	4	7	4.1		
62	PCE8494		3R	7,931,641	7,932,680	1,040	*CG31361*	+7815	***CG31361***	+7815	6	9	6	9	4.1		
63	PCE8495		2L	5,214,677	5,215,845	1,169	CG6514	+3847	***tkv***	+14084	6	10	5	9	4.1		
64	PCE8501		2L	5,247,719	5,248,767	1,049	***tkv***	+10898	Cyp4ac1	-7804	6	9	6	9	4.1		
65	PCE8511		3R	6,469,170	6,470,599	1,430	**hth**	-4766	CG6465	+32311	7	10	5	7	4.0		
66	PCE8512	pdm2 neurogenic	2L	12,663,453	12,664,721	1,269	***pdm2***	+2754	*pdm2*	+2754	5	12	4	9	4.0		
67	PCE8513		3L	14,550,945	14,551,746	802	**HGTX**	-2497	Cyp314a1	-16963	5	8	6	10	4.0		
68	PCE8515		2L	16,390,610	16,392,235	1,626	***BG:DS02780.1***	+34314	***BG:DS02780.1***	+34314	7	11	4	7	4.0		
69	PCE8519		3L	8,975,309	8,975,873	565	***Doc2***	+2077	***Doc2***	+2077	5	5	9	9	4.0	**Doc3**	11402
70	PCE8520		2L	12,080,772	12,081,448	677	prd	-5445	CG5325	-1193	4	8	6	12	4.0		
71	PCE8521		2L	7,252,370	7,253,008	639	CG31909	+2569	*Wnt4*	+16391	5	6	8	9	4.0	**Ndae1**	-19639
72	PCE8528		X	14,366,706	14,367,311	606	***NetA***	+17535	***NetA***	+17535	4	7	7	12	4.0		
73	PCE8531		3R	6,363,866	6,364,968	1,103	CG31394	-8970	***hth***	+66442	6	9	5	8	4.0		
74	PCE8533		3R	24,402,963	24,403,946	984	**fkh**	-2792	Noa36	+10421	6	8	6	8	3.9		
75	PCE8536		3R	12,764,472	12,765,970	1,499	***Abd-B***	+4036	***Abd-B***	+4036	7	10	5	7	3.9		

Seventy-five top pCRMs within 20 kb of a gene with early anterior-posterior expression, excluding those already listed in Table 3, are ranked by a z-score based on the number and density of conserved binding sites (see text for details). Site density columns list the number of conserved sites per kilobase (relative to the *D. melanogaster *sequence). The number and density of conserved sites are shown under two conditions - aligned sites only (A), or aligned + preserved sites (A+P) (see Materials and methods). The 5' and 3' gene columns correspond to the closest transcription (or annotation) start 5' and 3' of the pCRM. If a pCRM is within an intron, only the intron-containing gene is reported and its name is italicized. The names of genes with early anterior-posterior patterns are in bold. Early anterior-posterior genes that start within 20 kb of the pCRM (but are not the immediate annotation in the 5' or 3' direction) are also listed. Named enhancers without a reference are from this study.
