# Supplementary figures and images for: Computational identification of developmental enhancers: conservation and function of transcription factor binding-site clusters in Drosophila melanogaster and Drosophila pseudoobscura
Source: Genome Biol. 2004 Aug 20;5(9):R61. doi: 10.1186/gb-2004-5-9-r61 (PMC522868; doi:10.1186/gb-2004-5-9-r61)

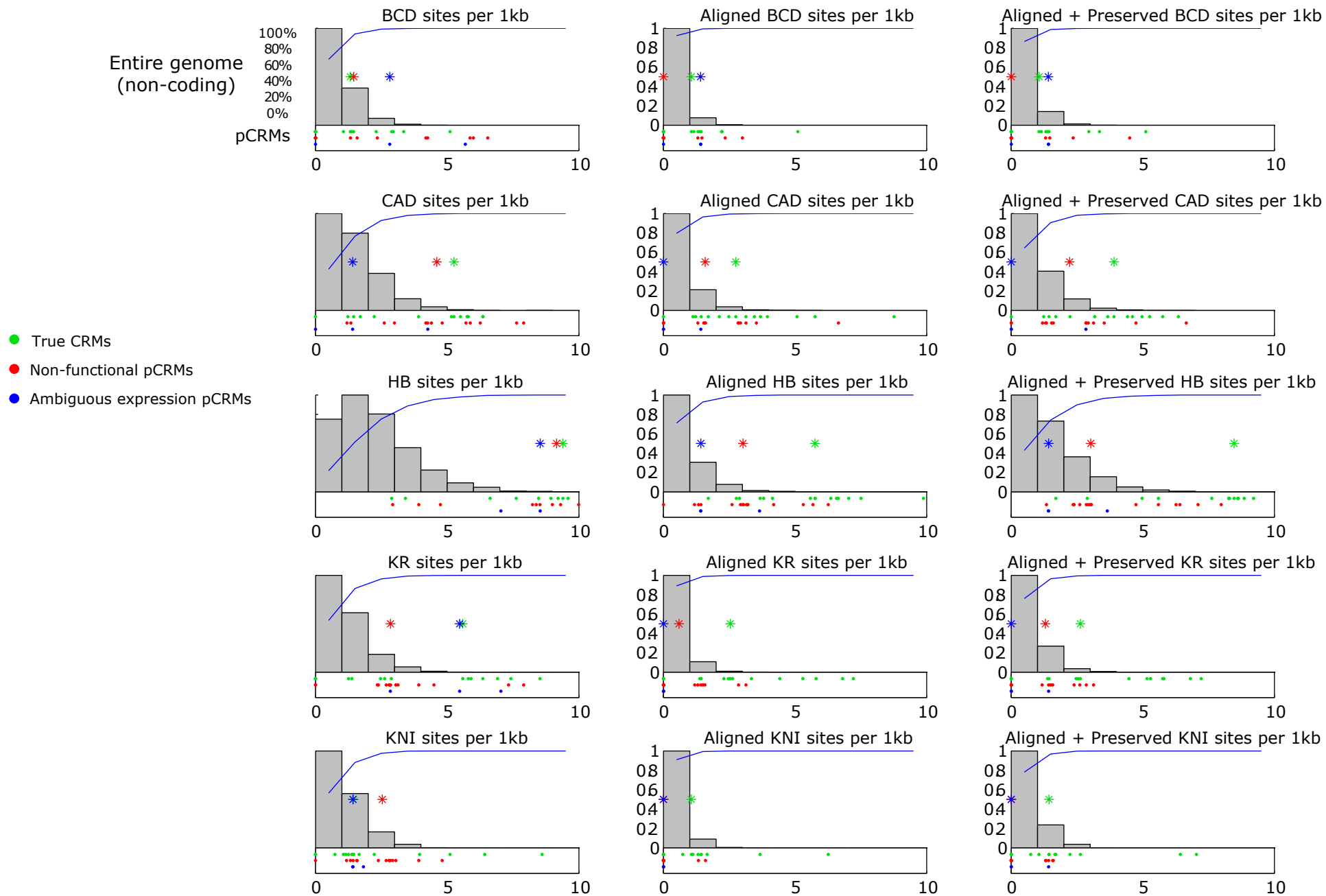

Supplement: Additional data file 1 — The binding site densities (column 1), aligned site densities (column 2), and aligned plus preserved site densities (column 3) for individual transcription factors [file gb-2004-5-9-r61-s1.pdf]

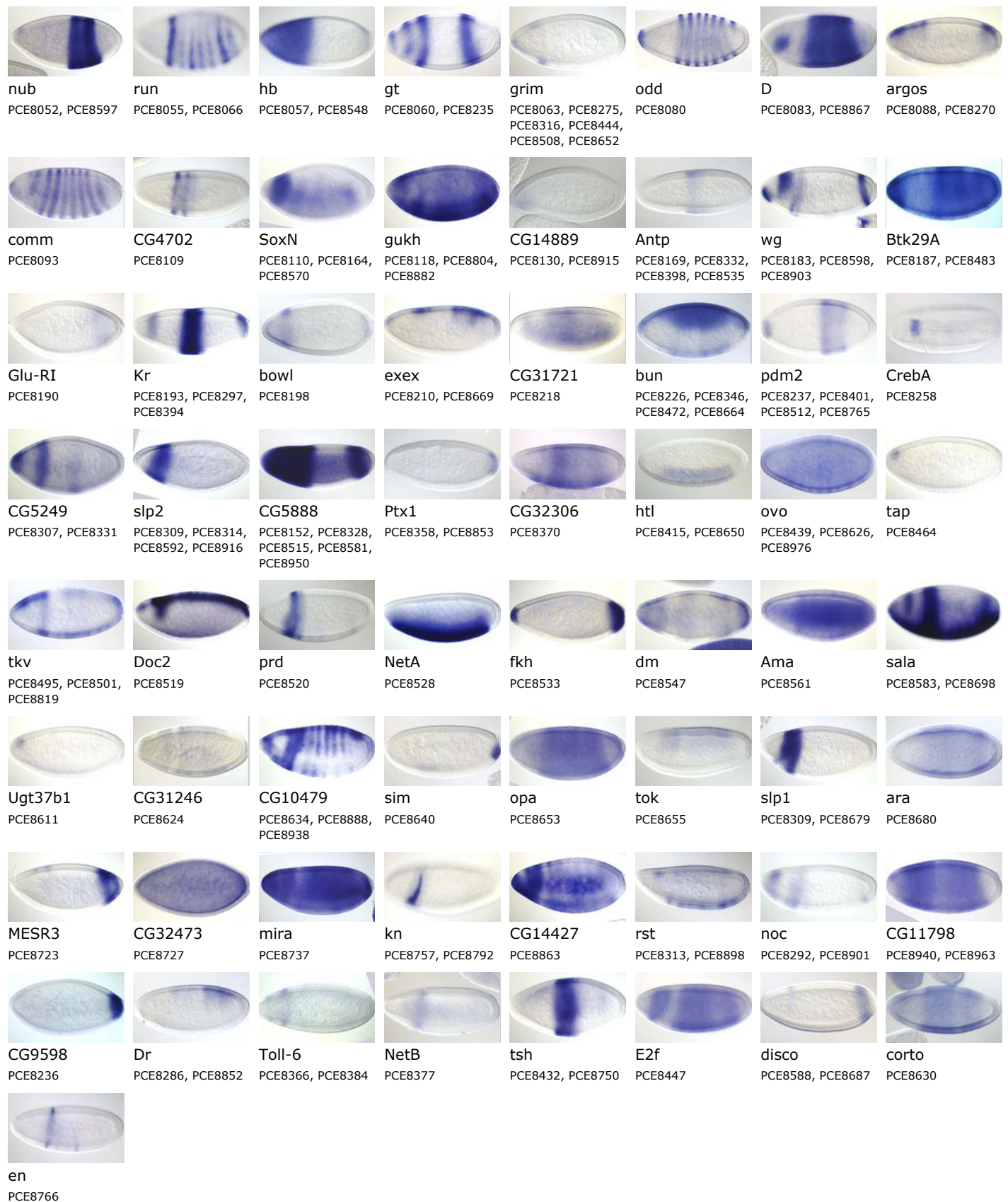

Supplement: Additional data file 2 — Expression patterns of 65 genes adjacent to 122 pCRMs identified by eCIS-ANALYST [file gb-2004-5-9-r61-s2.pdf]

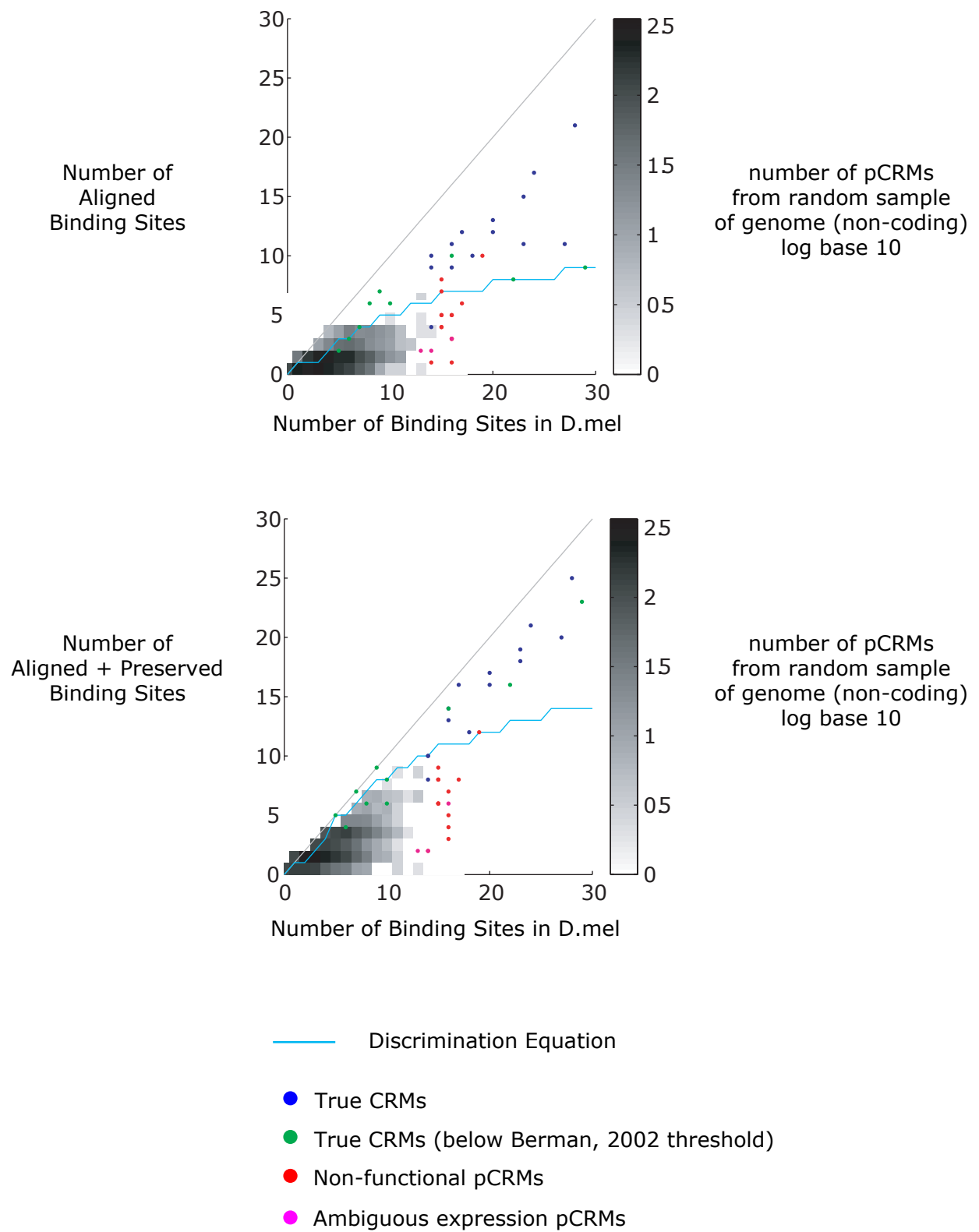

Supplement: Additional data file 3 — Discrimination of positive and negative pCRMs. Comparisons of the number of predicted binding sites in D. melanogaster pCRMs to the number of aligned sites (top panel) and aligned plus preserved sites (bottom panel) [file gb-2004-5-9-r61-s3.pdf]

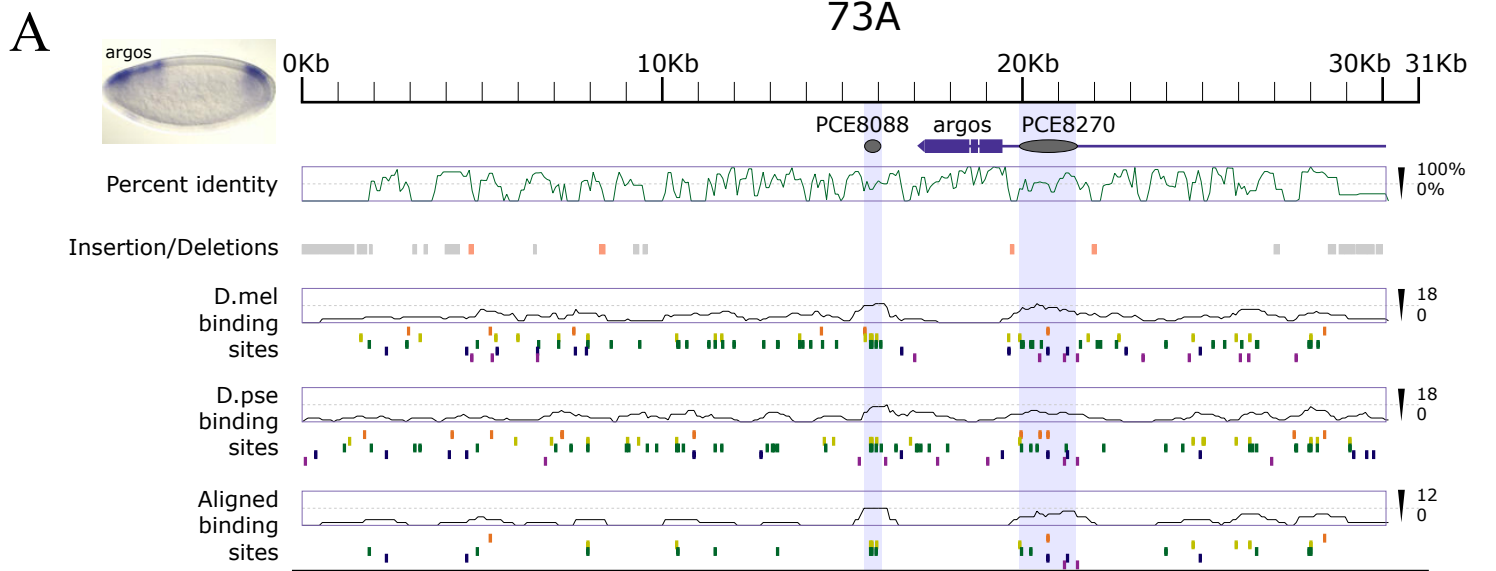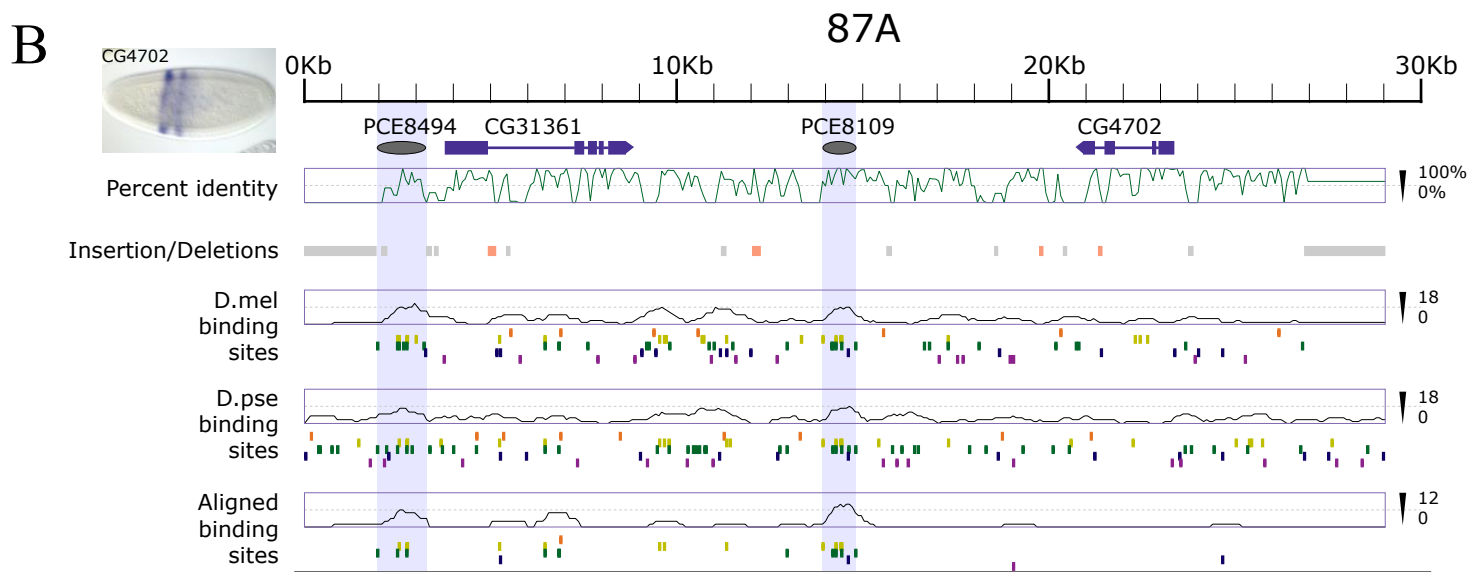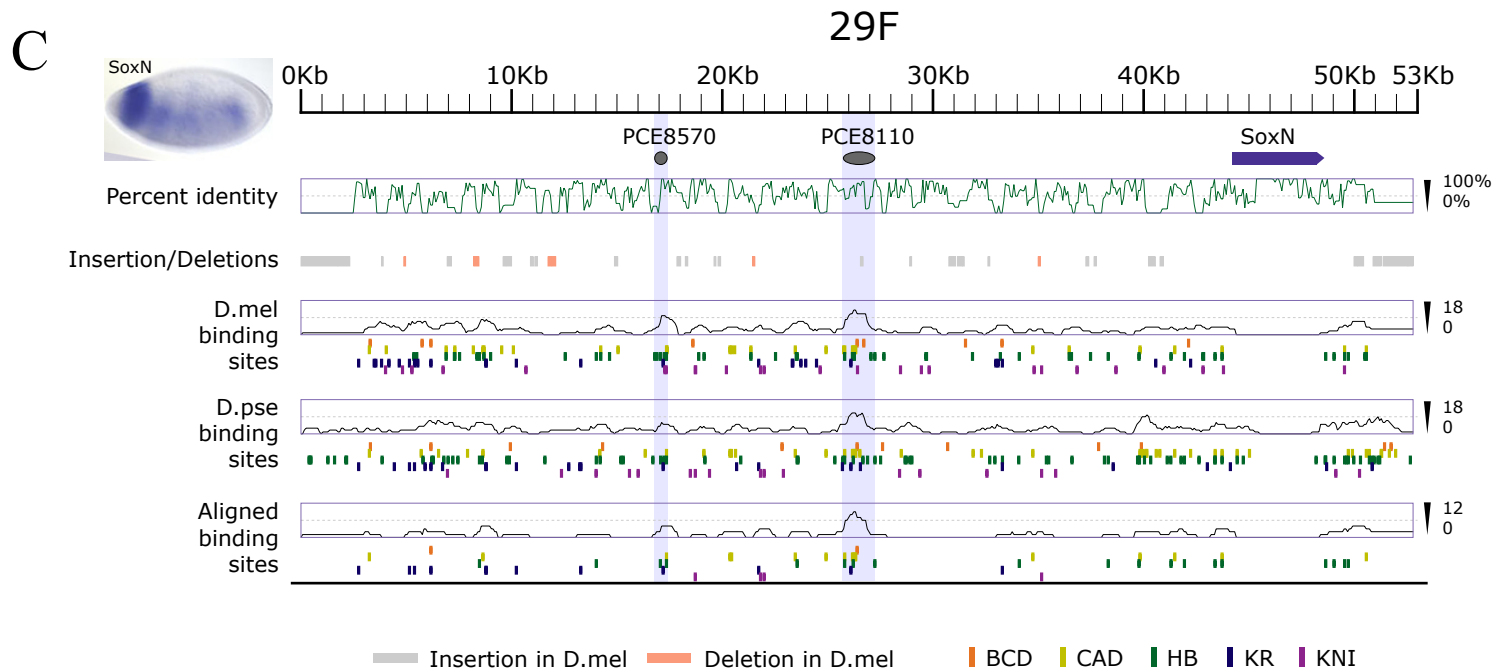

Supplement: Additional data file 4 — New pCRMs [file gb-2004-5-9-r61-s4.pdf]
