# Supplementary material for: Computational identification of developmental enhancers: conservation and function of transcription factor binding-site clusters in Drosophila melanogaster and Drosophila pseudoobscura
Source: Genome Biol. 2004 Aug 20;5(9):R61. doi: 10.1186/gb-2004-5-9-r61 (PMC522868; doi:10.1186/gb-2004-5-9-r61)
Supplement: Additional data file 5 — The primers used to amplify pCRMs for transgenics [file gb-2004-5-9-r61-s5.pdf]

pCRM	name	BPB2002 ID	Chrom arm	pCRM start	pCRM end	pCRM len	tested fragment start	tested fragment end	tested fragment len	forward primer	reverse primer
PCE7001	runt stripe 3		X	20,357,206	20,358,294	1,089	20,355,604	20,358,006	2,403		
PCE7002	eve stripes 3/7		2R	5,035,494	5,036,771	1,278	5,036,134	5,036,643	510		
PCE7003	eve stripe 2		2R	5,038,454	5,039,040	587	5,038,623	5,039,014	392		
PCE7004	eve stripes 4/6		2R	5,044,597	5,045,395	799	5,044,530	5,045,130	601		
PCE7005	hairy stripe 7		3L	8,624,351	8,625,245	895	8,623,788	8,624,718	931		
PCE7006	hairy stripe 6		3L	8,625,452	8,626,319	868	8,625,560	8,626,105	546		
PCE7007	hairy stripes 1,5		3L	8,629,180	8,629,966	787	8,628,790	8,630,509	1,720		
PCE7008	kni upstream		3L	20,615,070	20,616,425	1,356	20,615,080	20,616,107	1,028		
PCE7009	hb HZ1.4		3R	4,526,315	4,527,521	1,207	4,526,537	4,527,957	1,421		
PCE8001	gt posterior domain	1	X	2,187,439	2,188,382	944	2,187,356	2,188,453	1,098	5'-TTAGGCGCGCCAGAAACTTACCATCACTTCG-3'	5'-ATTGCGGCCGCCCCATTCAGGGGGATTGGG-3'
PCE8010	odd stripes 3/6	2	2L	3,601,750	3,602,509	760	3,601,404	3,602,776	1,373	5'-TTAGGCGCGCCAGAGCGTAAATGGCGTACTGA-3'	5'-ATTGCGGCCGCCTTTAGCCTCGGCGTTTG-3'
PCE8011	pdm1 blastoderm	3	2L	12,605,345	12,606,039	695	12,605,003	12,606,214	1,212	5'-TTAGGCGCGCCCATAGGTGAGTGCGAAAGGGT-3'	5'-ATTGCGGCCGCTGTGCATCAGGTGTTAAGCCA-3'
PCE8024	ftz stripes 1/5	4	3R	2,693,713	2,694,405	693	2,693,266	2,694,697	1,432	5'-TTAGGCGCGCCCAAATACAGTATGCGTCAGAA-3'	5'-ATTGCGGCCGCGGCCAAACGACAGACTAA-3'
PCE8012	pdm2 neurogenic	5	2L	12,663,878	12,664,600	723	12,663,605	12,664,915	1,311	5'-TTAGGCGCGCCGGAGTCGGTTGGTGTAGCG-3'	5'-ATTGCGGCCGCGGGTTTATGGGCATTAGTTGG-3'
PCE8027	sqz neurogenic	6	3R	15,000,096	15,000,905	810	14,999,957	15,001,318	1,362	5'-TTAGGCGCGCCGGCGAATCAAACACTTAAC-3'	5'-ATTGCGGCCGCAGATAAACAAATAACCGAAGG-3'
PCE8005	cluster_at_7A	7	X	6,996,209	6,996,756	548	6,995,933	6,997,124	1,192	5'-TTAGGCGCGCCGGCGATTCGGCAGTATCCAA-3'	5'-ATTGCGGCCGCCTGGCGGCTTCTCCTTCGTAT-3'
PCE8016	cluster_at_55C	8	2R	13,354,407	13,355,109	703	13,354,069	13,355,591	1,523	5'-TTAGGCGCGCCCAAGAAGACGCTGTCAGTTGC-3'	5'-ATTGCGGCCGCCTGTTGCCGTTCCGATTC-3'
PCE8020	cluster_at_70F	9	3L	14,665,967	14,666,676	710	14,664,995	14,666,459	1,465	5'-TTAGGCGCGCCGTTGACAGCCAGCCTAGATCC-3'	5'-ATTGCGGCCGCCAGGCAAGCAGAAGCGAG-3'
PCE8002	cluster_at_4B	10	X	4,124,119	4,125,459	1,341	4,123,730	4,125,859	2,130	5'-TTAGGCGCGCCTCTTCACGACGAGGACGA-3'	5'-ATTGCGGCCGCGGACAAGTGCGCCCATTA-3'
PCE8003	cluster_at_5C.1	11	X	5,658,504	5,659,131	628	5,658,257	5,659,392	1,136	5'-TTAGGCGCGCCCACACCAACACTAGCGCACAG-3'	5'-ATTGCGGCCGCCGTTGTTCTCGCCGAAGGTT-3'
PCE8004	cluster_at_5C.2	12	X	5,674,913	5,675,606	694	5,674,716	5,675,960	1,245	5'-TTAGGCGCGCCAATGGCATTTCGCTGTTC-3'	5'-ATTGCGGCCGCGGCGGTGTTGGCATAA-3'
PCE8006	cluster_at_7B	13	X	7,239,486	7,240,124	639	7,239,133	7,240,622	1,490	5'-TTAGGCGCGCCCTGCTGCTATGCGTGTAATTC-3'	5'-ATTGCGGCCGCTGCGTGTCCGTTAAACCA-3'
PCE8007	cluster_at_7F	14	X	8,350,658	8,351,315	658	8,350,159	8,351,727	1,569	5'-TTAGGCGCGCCTAGCGTAGATTTTTAACCCAT-3'	5'-ATTGCGGCCGCGGAACACATTTGGGATATG-3'
PCE8008	cluster_at_8F	15	X	9,457,631	9,458,375	745	9,457,472	9,458,697	1,226	5'-TTAGGCGCGCCACTCTCTGGAACTGCGAAACA-3'	5'-ATTGCGGCCGCTGGCCCAAACGAACGA-3'
PCE8009	cluster_at_12E	16	X	14,146,556	14,147,218	663	14,146,290	14,147,566	1,277	5'-TTAGGCGCGCCTATCCGAGCATCCGCCTAC-3'	5'-ATTGCGGCCGCCGGCGAAAGGACCTACCAAC-3'
PCE8013	cluster_at_34E	17	2L	13,989,283	13,990,132	850	13,989,186	13,990,523	1,338	5'-TTAGGCGCGCCAGTTGGCTTCGGGTGA-3'	5'-ATTGCGGCCGCGTGGCGATTCTAATTGTATGA-3'
PCE8014	cluster_at_36F	18	2L	18,400,758	18,401,458	701	18,400,400	18,401,689	1,290	5'-TTAGGCGCGCCGCCAAACTCTCCCTAAACCA-3'	5'-ATTGCGGCCGCGTAACAAATAAGCCGCACAAG-3'
PCE8015	cluster_at_47A	19	2R	5,664,440	5,665,094	655	5,664,034	5,665,331	1,298	5'-TTAGGCGCGCCTGTAGATACACTTCGGTTTCC-3'	5'-ATTGCGGCCGCATGATGCCCACATACTGAC-3'
PCE8017	cluster_at_56B	20	2R	14,266,629	14,267,261	633	14,266,335	14,267,714	1,380	5'-TTAGGCGCGCCAGTCCTAATCCTCGCCCATA-3'	5'-ATTGCGGCCGCTCAGTCAAAGTTGTCGCACAT-3'
PCE8018	cluster_at_59B	21	2R	17,995,894	17,996,609	716	17,995,467	17,996,855	1,389	5'-TTAGGCGCGCCGCAGCGAAGTGGTCTCAATCT-3'	5'-ATTGCGGCCGCCGGCTGCACTTACGTTTCAC-3'
PCE8019	cluster_at_67B	22	3L	9,529,913	9,530,579	667	9,529,465	9,530,697	1,233	5'-TTAGGCGCGCCAAATCCGCAAATGTAAGGTAT-3'	5'-ATTGCGGCCGCGGGCGAAAGACAAGTTAAA-3'
PCE8021	cluster_at_75C	23	3L	18,339,914	18,340,665	752	18,339,620	18,340,904	1,285	5'-TTAGGCGCGCCGAGCGAAGGATACAGGATCTA-3'	5'-ATTGCGGCCGCCGCACAGTCCACTCGTAA-3'
PCE8022	cluster_at_76C	24	3L	19,594,180	19,594,883	704	19,594,141	19,595,365	1,225	5'-TTAGGCGCGCCGCCGCAGGGAAGGTAAGTT-3'	5'-ATTGCGGCCGCACTTAATATGCACGGCGAGTG-3'
PCE8023	cluster_at_84A	25	3R	2,595,162	2,595,926	765	2,595,080	2,596,370	1,291	5'-TTAGGCGCGCCGGTTGCGGTTCGTGCTC-3'	5'-ATTGCGGCCGCAGTATCACTGGTTGGCGATGG-3'
PCE8025	cluster_at_85C	26	3R	4,944,607	4,945,444	838	4,944,225	4,945,690	1,466	5'-TTAGGCGCGCCAAAGCCACAGAACTCGCATAC-3'	5'-ATTGCGGCCGCGGCCTTGAGAAATGTTTACCA-3'
PCE8026	cluster_at_88F	27	3R	11,424,315	11,424,996	682	11,423,952	11,425,201	1,250	5'-TTAGGCGCGCCTGTCCTGCGACGAGTGTG-3'	5'-ATTGCGGCCGCAAATAAGTGGCATCCCGTTGT-3'
PCE8028	cluster_at_95C	28	3R	19,757,908	19,758,531	624	19,757,427	19,758,699	1,273	5'-TTAGGCGCGCCGGCATTTCTGGCATTTCGTCT-3'	5'-ATTGCGGCCGCGAACAAAGCCGAACGGACA-3'
