# Supplementary material for: Computational identification of developmental enhancers: conservation and function of transcription factor binding-site clusters in Drosophila melanogaster and Drosophila pseudoobscura
Source: Genome Biol. 2004 Aug 20;5(9):R61. doi: 10.1186/gb-2004-5-9-r61 (PMC522868; doi:10.1186/gb-2004-5-9-r61)
Supplement: Additional data file 6 — Additional information from Table 2 [file gb-2004-5-9-r61-s6.pdf]

pCRM	name	BPB2002 ID	Chrom arm	pCRM start	pCRM end	pCRM len	D.pse scaffold	Alignment length	D.pse start	D.pse end	Dpse len	Dmel noncoding bases	Dpse noncoding bases
PCE7001	runt stripe 3		X	20,357,206	20,358,294	1,089	Dpse_3212383_1_44815	61,982	17,707	19,210	1,504	18,864	17,308
PCE7002	eve stripes 3/7		2R	5,035,494	5,036,771	1,278	Dpse_3213009_3212129_1_42085	56,885	19,302	20,415	1,114	14,177	12,863
PCE7003	eve stripe 2		2R	5,038,454	5,039,040	587	Dpse_3213009_3212129_1_41074	56,583	19,594	20,364	771	14,959	14,824
PCE7004	eve stripes 4/6		2R	5,044,597	5,045,395	799	Dpse_3213009_3212129_1_41218	56,239	21,774	22,776	1,003	15,405	12,863
PCE7005	hairy stripe 7		3L	8,624,351	8,625,245	895	Dpse_3212377_1_51998	62,992	24,614	25,482	869	19,610	20,337
PCE7006	hairy stripe 6		3L	8,625,452	8,626,319	868	Dpse_3212377_1_51998	62,992	25,642	26,593	952	19,509	19,782
PCE7007	hairy stripes 1,5		3L	8,629,180	8,629,966	787	Dpse_3212377_1_52744	63,742	26,928	27,650	723	17,879	18,656
PCE7008	kni upstream		3L	20,615,070	20,616,425	1,356	Dpse_3213110_1_51190	61,930	23,760	25,413	1,654	17,207	16,065
PCE7009	hb HZ1.4		3R	4,526,315	4,527,521	1,207	Dpse_3213225_3212440_1_37623	58,662	21,040	22,422	1,383	16,823	15,791
PCE8001	gt posterior domain	1	X	2,187,439	2,188,382	944	Dpse_3212166_3212206_3213217_1_45443	62,659	16,447	17,538	1,092	17,609	16,370
PCE8010	odd stripes 3/6	2	2L	3,601,750	3,602,509	760	Dpse_3213134_1_63579	71,306	26,093	26,917	825	17,010	28,745
PCE8011	pdm1 blastoderm	3	2L	12,605,345	12,606,039	695	Dpse_3212646_1_58383	66,165	28,248	29,141	894	15,893	16,943
PCE8024	ftz stripes 1/5	4	3R	2,693,713	2,694,405	693	Dpse_3212942_1_49671	59,840	24,755	25,498	744	18,242	16,659
PCE8012	pdm2 neurogenic	5	2L	12,663,878	12,664,600	723	Dpse_3212646_3212123_3213189_1_68286	74,438	28,671	29,393	723	15,638	18,408
PCE8027	sqz neurogenic	6	3R	15,000,096	15,000,905	810	Dpse_3212786_3212587_1_36348	58,805	11,368	12,185	818	13,520	11,955
PCE8005	cluster_at_7A	7	X	6,996,209	6,996,756	548	Dpse_3213012_1_61104	69,604	28,638	29,456	819	20,000	24,123
PCE8016	cluster_at_55C	8	2R	13,354,407	13,355,109	703	Dpse_3212407_1_50564	59,819	24,616	26,232	1,617	10,316	9,120
PCE8020	cluster_at_70F	9	3L	14,665,967	14,666,676	710	Dpse_3213050_1_40411	57,919	23,210	23,747	538	20,000	16,281
PCE8002	cluster_at_4B	10	X	4,124,119	4,125,459	1,341	Dpse_3212444_1_57148	69,031	27,381	27,452	72	19,172	21,268
PCE8003	cluster_at_5C.1	11	X	5,658,504	5,659,131	628	Dpse_3208392_3212169_1_53199	81,985	28,460	28,588	129	13,848	5,725
PCE8004	cluster_at_5C.2	12	X	5,674,913	5,675,606	694	Dpse_3215593_1_56294	76,411	28,230	29,072	843	16,052	13,136
PCE8006	cluster_at_7B	13	X	7,239,486	7,240,124	639	Dpse_3213012_1_57731	69,290	25,639	26,301	663	19,143	24,693
PCE8007	cluster_at_7F	14	X	8,350,658	8,351,315	658	Dpse_3213180_3212715_3211870_1_47844	61,404	24,542	24,670	129	16,259	18,271
PCE8008	cluster_at_8F	15	X	9,457,631	9,458,375	745	Dpse_3212341_1_51935	62,233	24,374	25,089	716	20,000	19,255
PCE8009	cluster_at_12E	16	X	14,146,556	14,147,218	663	Dpse_3212152_1_43438	64,357	24,716	25,353	638	19,714	16,505
PCE8013	cluster_at_34E	17	2L	13,989,283	13,990,132	850	Dpse_3211777_1_59057	68,087	29,315	30,233	919	18,451	23,991
PCE8014	cluster_at_36F	18	2L	18,400,758	18,401,458	701	Dpse_3212190_1_56443	73,446	23,823	24,418	596	20,000	30,680
PCE8015	cluster_at_47A	19	2R	5,664,440	5,665,094	655	Dpse_3213122_1_27515	55,252	14,195	14,846	652	17,429	15,278
PCE8017	cluster_at_56B	20	2R	14,266,629	14,267,261	633	Dpse_3213226_3212141_1_38431	56,565	16,180	16,510	331	19,579	18,004
PCE8018	cluster_at_59B	21	2R	17,995,894	17,996,609	716	Dpse_3212648_1_45519	61,494	21,221	22,180	960	18,871	14,029
PCE8019	cluster_at_67B	22	3L	9,529,913	9,530,579	667	Dpse_3212377_1_44926	57,956	24,327	25,001	675	19,664	15,627
PCE8021	cluster_at_75C	23	3L	18,339,914	18,340,665	752	Dpse_3212212_1_56415	67,137	31,359	31,998	640	19,149	18,670
PCE8022	cluster_at_76C	24	3L	19,594,180	19,594,883	704	Dpse_3213110_1_49535	60,524	22,994	23,718	725	14,158	14,759
PCE8023	cluster_at_84A	25	3R	2,595,162	2,595,926	765	Dpse_3212942_3212942_1_46935	59,140	23,130	24,130	1,001	18,018	18,086
PCE8025	cluster_at_85C	26	3R	4,944,607	4,945,444	838	Dpse_3213126_1_55976	65,240	27,604	28,430	827	20,000	24,088
PCE8026	cluster_at_88F	27	3R	11,424,315	11,424,996	682	Dpse_3212649_1_45115	63,991	28,504	29,599	1,096	20,000	15,310
PCE8028	cluster_at_95C	28	3R	19,757,908	19,758,531	624	Dpse_3213015_3213015_3213215_1_54769	63,690	22,188	22,910	723	13,089	20,012
