# Supplementary material for: Computational identification of developmental enhancers: conservation and function of transcription factor binding-site clusters in Drosophila melanogaster and Drosophila pseudoobscura
Source: Genome Biol. 2004 Aug 20;5(9):R61. doi: 10.1186/gb-2004-5-9-r61 (PMC522868; doi:10.1186/gb-2004-5-9-r61)
Supplement: Additional data file 7 — All new pCRMs from genome-wide eCIS-ANALYST located within 20 kb of annotated transcript [file gb-2004-5-9-r61-s7.pdf]

	CRM	Overlaps known element	Chrom arm	pCRM start	pCRM end	pCRM len	5' gene	pCRM relative position	3' gene	pCRM relative position	Aligned sites	Aligned + preserved sites	Aligned site dens	Aligned + preserved site dens	z-score	Additional Gap/pair-rule gene within 20kb	pCRM relative position
1	PCE8050	hairy stripes 3/4,6,7	3L	8,622,879	8,626,839	3,961	CG6486	+14646	**h**	-7829	36	62	9	16	20.1		
2	PCE8051	kni upstream	3L	20,614,714	20,617,020	2,307	**kni**	-813	CG13253	+20716	25	31	11	13	13.2		
3	PCE8052	pdm1 blastoderm	2L	12,604,311	12,606,913	2,603	CG15488	+1653	**nub**	-304	20	33	8	13	11.6		
4	PCE8053	eve stripes 3/7	2R	5,035,493	5,037,290	1,798	CG12134	+3712	**eve**	-2433	21	24	12	13	11.5	**Adam**	+5901
5	PCE8054	hairy stripes 1,5	3L	8,628,846	8,631,011	2,166	CG6486	+20613	**h**	-3657	17	29	8	13	10.5		
6	PCE8055	runt stripe 3	X	20,356,848	20,360,054	3,207	CG1338	-9192	**run**	-6801	17	34	5	11	10.3		
7	PCE8056		X	20,323,964	20,326,397	2,434	CG11692	-12536	Cyp6v1	-4186	16	28	7	12	9.6		
8	PCE8057	hb HZ1.4	3R	4,526,225	4,527,991	1,767	**hb**	-2670	CG8112	+1273	17	21	10	12	9.5		
9	PCE8059	eve stripes 4/6	2R	5,044,597	5,046,030	1,434	**eve**	+4874	TER94	-3763	15	18	10	13	9.0	**Adam**	+15005
10	PCE8060	gt posterior domain	X	2,186,709	2,189,069	2,361	**gt**	-974	tko	+11679	18	21	8	9	8.9		
11	PCE8061		X	3,169,806	3,172,348	2,543	CG12535	-17954	CG14269	+21857	13	29	5	11	8.8		
12	PCE8063	CE8021	3L	18,339,914	18,341,941	2,028	**grim**	-86621	**rpr**	+5341	16	20	8	10	8.5		
13	PCE8064		3R	6,255,663	6,256,945	1,283	CG6345	-13879	Cyp12e1	-3594	13	17	10	13	8.4		
14	PCE8065		3R	4,026,032	4,027,816	1,785	**grn**	-18853	CG7800	-15898	15	19	8	11	8.4		
15	PCE8066		X	20,348,460	20,352,624	4,165	CG1338	-804	**run**	-14231	16	28	4	7	8.3		
16	PCE8067	ftz upstream (partial)	3R	2,682,314	2,684,591	2,278	**Scr**	-7972	**ftz**	-5455	15	22	7	10	8.3		
17	PCE8068		X	18,701,007	18,702,700	1,694	CG32541	+39691	CG32541	+39691	12	22	7	13	8.2		
18	PCE8069		2R	17,274,311	17,276,017	1,707	CG3380	-2521	dve	-11496	14	19	8	11	8.2		
19	PCE8070		2L	7,616,050	7,618,366	2,317	CG6739	+15430	CG13792	+19862	14	23	6	10	8.1		
20	PCE8071	sqz neurogenic	3R	14,999,463	15,001,552	2,090	sqz	+9504	CG14282	-1186	12	24	6	11	8.0	**nos**	+16485
21	PCE8072		X	5,674,422	5,676,386	1,965	CG3726	+16870	CG12728	-6597	11	24	6	12	7.8		
22	PCE8073		2R	14,903,099	14,903,925	827	Toll-7	+12482	Obp56i	-27903	11	11	13	13	7.8		
23	PCE8074		3R	23,192,304	23,192,750	447	CG13980	+8073	side	+40862	7	8	16	18	7.7		
24	PCE8075		3R	10,762,920	10,764,750	1,831	CG3837	+18501	CG14861	-75759	13	19	7	10	7.6		
25	PCE8076	eve stripe 2	2R	5,038,454	5,039,041	588	CG12134	+6673	**eve**	-682	8	10	14	17	7.6	**Adam**	+8862
26	PCE8077		2L	13,541,662	13,542,651	990	kuz	+9371	kuz	+9371	11	13	11	13	7.6		
27	PCE8078		2L	14,424,056	14,425,158	1,103	BG:DS06238.4	-16773	BG:DS08340.1	+7810	12	13	11	12	7.6		
28	PCE8080	odd stripes 3/6	2L	3,601,045	3,602,748	1,704	**odd**	-1728	Dot	-9112	12	19	7	11	7.5		
29	PCE8081		3L	17,412,324	17,413,414	1,091	CG18265	+24035	CG7603	-1413	11	14	10	13	7.5		
30	PCE8083		3L	14,121,556	14,123,127	1,572	Sox21b	-41352	**D**	+4373	12	17	8	11	7.3		
31	PCE8084		2L	4,098,489	4,099,006	518	ed	+74542	ed	+74542	7	9	14	17	7.3		
32	PCE8085		2R	12,253,766	12,255,302	1,537	CG10953	-23540	CG10950	-3625	13	15	8	10	7.2		
33	PCE8086		3L	20,612,647	20,614,073	1,427	**kni**	+1254	CG13253	+23663	11	17	8	12	7.2		
34	PCE8087		2R	3,391,037	3,391,561	525	CG30358	+10444	CG14755	-16724	7	9	13	17	7.2		
35	PCE8088		3L	16,418,107	16,418,469	363	CG33158	+49435	**argos**	+14111	6	6	17	17	7.2		
36	PCE8089		3R	12,368,159	12,368,687	529	CG11769	+28970	CG31448	-670	7	9	13	17	7.2	**CG14889**	-13735
37	PCE8091		3L	11,213,064	11,213,664	601	scylla	+3224	CG32083	+24695	8	9	13	15	7.1		
38	PCE8092		2L	1,233,357	1,235,228	1,872	CG5156	+3715	CG5397	-6475	9	23	5	12	7.1		
39	PCE8093		3L	15,688,222	15,691,204	2,983	**comm**	-10920	CG13445	-67172	13	22	4	7	7.0		
40	PCE8094		2R	10,492,861	10,493,546	686	CG30472	-5321	CG12959	-26488	9	9	13	13	7.0		
41	PCE8095		3R	23,894,562	23,895,459	898	CG12870	+31901	CG12870	+31901	10	11	11	12	7.0		
42	PCE8096		3L	6,762,543	6,765,157	2,615	vvl	+12855	Prat2	+108336	13	20	5	8	6.9		
43	PCE8097		3R	10,238,130	10,238,652	523	CG14846	-1983	CG14847	+4557	7	8	13	15	6.8		
44	PCE8099		2L	18,305,051	18,306,251	1,201	Fas3	+6868	Fas3	+6868	10	14	8	12	6.7		
45	PCE8100	eve early APR	2R	5,042,174	5,042,884	711	**eve**	+2451	TER94	-6909	8	10	11	14	6.7	**Adam**	+12582
46	PCE8102	tll posterior	3R	26,663,942	26,665,204	1,263	CG15544	+21005	**tll**	-2251	11	13	9	10	6.6		
47	PCE8104	ems neurogenic	3R	9,723,602	9,724,936	1,335	E5	-23682	**ems**	-2663	12	12	9	9	6.6		
48	PCE8105		3R	17,817,909	17,818,791	883	Eip93F	+25598	Eip93F	+25598	9	11	10	12	6.6		
49	PCE8106		3L	10,499,018	10,501,551	2,534	CG32062	+25485	CG32062	+25485	11	21	4	8	6.6		
50	PCE8107		3L	4,612,891	4,614,005	1,115	CG13716	-161	CG13715	+1681	11	11	10	10	6.6		
51	PCE8108		2L	14,403,771	14,404,937	1,167	CG15284	-4301	BG:DS06238.4	+2346	10	13	9	11	6.5		
52	PCE8109		3R	7,941,601	7,942,426	826	CG31361	+17775	**CG4702**	+11512	9	10	11	12	6.5		
53	PCE8110		2L	8,804,166	8,805,336	1,171	CG9468	-30684	**SoxN**	-12519	10	13	9	11	6.5		
54	PCE8111		3L	8,612,337	8,613,016	680	CG6486	+4104	**h**	-21652	8	9	12	13	6.5		
55	PCE8112		3L	4,377,989	4,379,208	1,220	CG7447	+13842	Syx17	-3984	11	12	9	10	6.5		
56	PCE8113		2L	14,113,291	14,113,893	603	CG15292	-3974	CG31768	-6693	7	9	12	15	6.5		
57	PCE8114		3L	3,997,600	3,998,923	1,324	CG14985	+13500	fd64A	-799	11	13	8	10	6.5		
58	PCE8115	eve stripe 1	2R	5,046,559	5,047,297	739	**eve**	+6836	TER94	-2496	8	10	11	14	6.5	**Adam**	+16967
59	PCE8116		2R	16,921,501	16,922,240	740	CG13493	-11091	PpN58A	+4194	8	10	11	14	6.5		
60	PCE8118		3R	14,822,848	14,823,484	637	**gukh**	+13085	**gukh**	+13085	8	8	13	13	6.4		
61	PCE8119		3R	12,671,525	12,672,987	1,463	**abd-A**	-15737	CG10349	-32477	11	14	8	10	6.4		
62	PCE8120		3L	10,492,688	10,495,539	2,852	CG32062	+19155	CG32062	+19155	10	23	4	8	6.4		
63	PCE8121		2L	16,841,696	16,842,392	697	CG6012	-2193	CG31781	-5178	8	9	11	13	6.4		

	CRM	Overlaps known element	Chrom arm	pCRM start	pCRM end	pCRM len	5' gene	pCRM relative position	3' gene	pCRM relative position	Aligned sites	Aligned + preserved sites	Aligned site dens	Aligned + preserved site dens	z-score	Additional Gap/pair-rule gene within 20kb	pCRM relative position
64	PCE8122		3L	6,885,832	6,887,436	1,605	Prat2	-11445	CG14820	-5022	11	15	7	9	6.4		
65	PCE8123		2L	15,162,778	15,164,524	1,747	BG:DS03192.2	-6373	BG:DS07295.1	+59479	11	16	6	9	6.4		
66	PCE8124		2R	6,888,483	6,889,700	1,218	CG12443	+13963	CG13192	-428	10	13	8	11	6.4		
67	PCE8125		2L	20,466,022	20,467,708	1,687	CG2493	-32831	CG15476	+4184	10	17	6	10	6.4		
68	PCE8126		3L	2,779,198	2,779,658	461	CG2083	+1101	CG2083	+1101	6	7	13	15	6.3		
69	PCE8127		X	4,630,473	4,632,106	1,634	CG12681	+14179	CG15470	-3196	9	18	6	11	6.3		
70	PCE8128		3R	27,713,381	27,715,087	1,707	heph	+35171	heph	+35171	10	17	6	10	6.3		
71	PCE8130		3R	12,383,752	12,385,269	1,518	**CG14889**	+1858	**CG14889**	+1858	11	14	7	9	6.3		
72	PCE8131		3R	21,329,716	21,331,058	1,343	CG5111	+8355	msi	-2351	8	17	6	13	6.3		
73	PCE8132		3R	16,242,660	16,243,128	469	CG10881	+8657	CG17208	+20535	6	7	13	15	6.3		
74	PCE8133		3R	24,120,296	24,122,240	1,945	CG12516	-668	larp	+19112	12	15	6	8	6.2		
75	PCE8134		3L	8,733,754	8,734,394	641	CG32030	+8601	CG32030	+8601	7	9	11	14	6.2		
76	PCE8135		X	17,716,817	17,717,958	1,142	Sh	+3615	Sh	+3615	9	13	8	11	6.2		
77	PCE8137		3R	12,053,627	12,055,472	1,846	**tara**	+2239	**tara**	+2239	10	17	5	9	6.1		
78	PCE8138		X	7,396,751	7,398,239	1,489	ct	+24149	ct	+24149	9	16	6	11	6.1		
79	PCE8139		2R	6,573,169	6,574,383	1,215	inv	+32752	CG30034	+12378	10	12	8	10	6.1	**en**	+19407
80	PCE8140		2R	15,167,055	15,168,270	1,216	CG16898	-98356	**18w**	-6952	10	12	8	10	6.1		
81	PCE8141		3R	10,893,081	10,893,688	608	CG12601	-3999	CG31114	+9257	7	8	12	13	6.1		
82	PCE8142		3R	9,911,719	9,912,536	818	foxo	+29007	CG3153	-2727	8	10	10	12	6.1		
83	PCE8144		3L	3,503,831	3,504,156	326	Eip63E	+7518	Eip63E	+7518	4	6	12	18	6.1	**ImpE2**	-10525
84	PCE8145		3R	4,536,237	4,536,936	700	CG8112	+1795	CG8112	+1795	8	8	11	11	6.0	**hb**	-12682
85	PCE8146		2R	16,824,156	16,825,175	1,020	CG4021	+7261	lox2	+10031	9	11	9	11	6.0		
86	PCE8147		2L	4,145,728	4,147,090	1,363	CG2955	+3881	Or24a	+11580	8	16	6	12	6.0		
87	PCE8148		2L	11,942,898	11,943,920	1,023	CG16964	+6063	CG17745	-11559	9	11	9	11	6.0		
88	PCE8149		2R	18,266,546	18,267,656	1,111	CG3162	-11439	CG3092	-8537	9	12	8	11	6.0		
89	PCE8150		3R	6,379,567	6,380,474	908	**hth**	+50936	**hth**	+50936	8	11	9	12	6.0		
90	PCE8151		2L	15,776,939	15,777,643	705	BG:DS02252.3	+14601	BG:DS02252.4	+36049	6	11	9	16	6.0		
91	PCE8152		2L	16,363,535	16,364,908	1,374	BG:DS09218.5	+19533	**BG:DS02780.1**	+61641	8	16	6	12	6.0		
92	PCE8153		2L	754,034	754,662	629	Eaat2	-7974	GABA-B-R3	-121	7	8	11	13	5.9		
93	PCE8154		2R	14,014,526	14,016,536	2,011	sano	+3479	sano	+3479	10	17	5	8	5.9		
94	PCE8155		X	2,143,383	2,144,016	634	EG:BACH7M4.1	+420	EG:BACH7M4.1	+420	7	8	11	13	5.9		
95	PCE8156		3R	7,009,831	7,010,437	607	Ugt86Dh	+8520	CG18577	-11408	6	9	10	15	5.9		
96	PCE8158		2R	18,241,861	18,243,915	2,055	CG13539	+13886	CG3162	+11192	10	17	5	8	5.9		
97	PCE8159		3R	22,414,628	22,415,746	1,119	CG5467	+1870	CG5467	+1870	10	10	9	9	5.9		
98	PCE8160		2L	14,699,578	14,700,407	830	BG:DS07721.3	+4759	BG:DS07721.6	+10594	7	11	8	13	5.9		
99	PCE8161		3R	11,285,379	11,285,970	592	CG14869	+16554	CG31297	-5441	5	10	8	17	5.8		
100	PCE8162		2L	5,444,551	5,445,324	774	CG31647	-3134	CG6634	-8531	7	10	9	13	5.8		
101	PCE8163		2L	11,333,117	11,334,119	1,003	CG14926	-983	salr	-20056	9	10	9	10	5.8		
102	PCE8164		2L	8,839,488	8,841,115	1,628	**SoxN**	+21633	CG32986	-13922	10	14	6	9	5.8		
103	PCE8165		X	8,390,109	8,392,075	1,967	**oc**	-513	CG12772	-23984	10	16	5	8	5.8		
104	PCE8166		3R	12,570,467	12,571,123	657	**Ubx**	-10101	CG31275	+5951	7	8	11	12	5.7		
105	PCE8167	Ubx S1	3R	12,589,099	12,589,755	657	**CG31275 (Ubx adj.)**	-11970	Glut3	-24295	7	8	11	12	5.7		
106	PCE8168		3R	22,448,085	22,448,743	659	CG5476	+1126	CG31080	-77	7	8	11	12	5.7		
107	PCE8169	ftz stripes 1/5	3R	2,693,336	2,694,915	1,580	**ftz**	+3290	**Antp**	+63624	11	12	7	8	5.7		
108	PCE8170		3R	2,670,658	2,672,242	1,585	**Scr**	+2100	**Scr**	+2100	9	15	6	9	5.7	**ftz**	-19388
109	PCE8171		X	1,302,444	1,303,108	665	EG:196F3.2	+31949	EG:56G7.1	+11836	7	8	11	12	5.7		
110	PCE8172		3L	18,399,768	18,400,890	1,123	skl	-10952	CG32196	+8305	9	11	8	10	5.7		
111	PCE8173		3R	9,681,376	9,682,499	1,124	CG14362	-186	E5	+17421	9	11	8	10	5.7		
112	PCE8174		3L	11,293,375	11,294,546	1,172	CG6168	-14124	CG6163	+27871	8	13	7	11	5.7		
113	PCE8175		2R	18,095,222	18,095,804	583	twi	+5547	CG30194	-3259	6	8	10	14	5.7		
114	PCE8176		X	17,123,624	17,125,668	2,045	B-H2	+78085	B-H1	-2791	10	16	5	8	5.7		
115	PCE8177		2R	5,634,520	5,635,604	1,085	**psq**	+4661	**psq**	+4661	8	12	7	11	5.7		
116	PCE8178		3R	4,940,064	4,941,355	1,292	pum	+41733	pum	+41733	8	14	6	11	5.7		
117	PCE8179		2L	1,211,417	1,212,502	1,086	CG14342	+5436	CG14343	-8526	8	12	7	11	5.7		
118	PCE8180		3R	26,349,418	26,350,118	701	spdo	+1448	spdo	+1448	6	10	9	14	5.7		
119	PCE8182		3R	15,393,929	15,394,541	613	Dys	+64400	CG7344	-409	5	10	8	16	5.6		
120	PCE8183		2L	7,305,525	7,305,940	416	**wg**	+4205	**wg**	+4205	5	6	12	14	5.6		
121	PCE8184		3R	3,135,927	3,137,173	1,247	rn	-1859	CG32468	+3477	9	12	7	10	5.6		
122	PCE8185		3R	710,838	711,575	738	CG14660	-6584	Gnf1	-20769	7	9	9	12	5.6		
123	PCE8186		3L	8,453,917	8,454,507	591	CG6902	-896	CG6694	+13950	6	8	10	14	5.6		
124	PCE8187		2L	8,286,022	8,287,399	1,378	**Btk29A**	+5904	**Btk29A**	+5904	9	13	7	9	5.6		
125	PCE8188		3L	22,062,292	22,063,071	780	msopa	+62207	CG15374	-19633	8	8	10	10	5.6		
126	PCE8189		2R	6,792,459	6,794,046	1,588	CG13194	+3824	CG13193	-39119	10	13	6	8	5.6		

	CRM	Overlaps known element	Chrom arm	pCRM start	pCRM end	pCRM len	5' gene	pCRM relative position	3' gene	pCRM relative position	Aligned sites	Aligned + preserved sites	Aligned site dens	Aligned + preserved site dens	z-score	Additional Gap/pair-rule gene within 20kb	pCRM relative position
127	PCE8190		3L	6,589,453	6,590,721	1,269	**Glu-RI**	+5891	**Glu-RI**	+5891	9	12	7	9	5.6		
128	PCE8191		3L	1,385,892	1,386,270	379	CG32320	+6660	CG9168	-5584	5	5	13	13	5.6		
129	PCE8192		3R	23,242,393	23,243,019	627	side	-8781	CG13978	-36256	7	7	11	11	5.6		
130	PCE8193	Kr CD2	2R	20,268,656	20,269,940	1,285	CG9380	-36249	**Kr**	-244	7	15	5	12	5.5		
131	PCE8194		3R	23,969,559	23,970,425	867	CG12425	+7648	CG4787	-79761	8	9	9	10	5.5		
132	PCE8195		3L	5,126,445	5,126,805	361	**CG32423**	+17297	**CG32423**	+17297	4	6	11	17	5.5		
133	PCE8196		3L	2,826,468	2,828,094	1,627	CG2083	-45709	CG14952	+430	10	13	6	8	5.5		
134	PCE8197		2L	16,126,643	16,128,124	1,482	BG:DS04095.3	-11046	Ca-alpha1D	-20616	8	15	5	10	5.5		
135	PCE8198		2L	3,767,311	3,769,396	2,086	**bowl**	+2110	**bowl**	+2110	9	17	4	8	5.5		
136	PCE8199		3L	15,431,502	15,432,168	667	CG7804	-8513	ran-like	+2113	6	9	9	13	5.5		
137	PCE8200		2L	15,548,655	15,549,454	800	BG:DS04862.2	+20100	kek3	-7582	6	11	8	14	5.5		
138	PCE8201		2L	5,380,416	5,381,185	770	nompC	+41609	H15	-15371	7	9	9	12	5.5		
139	PCE8202		2L	2,624,801	2,625,854	1,054	CG15395	+22089	CG9962	+14358	8	11	8	10	5.5		
140	PCE8203		2R	14,009,681	14,010,690	1,010	sano	+333	sano	+333	7	12	7	12	5.5		
141	PCE8204		3L	12,572,530	12,573,035	506	caup	+4858	caup	+4858	6	6	12	12	5.5		
142	PCE8205		2R	7,062,819	7,064,497	1,679	CG8298	+8959	otk	+20040	10	13	6	8	5.5		
143	PCE8206		3R	21,617,170	21,618,622	1,453	CG31093	-11612	CG5024	-7560	7	16	5	11	5.5		
144	PCE8207		X	7,355,969	7,356,818	850	ct	+6445	ct	+6445	7	10	8	12	5.4		
145	PCE8208		2L	11,844,176	11,844,657	482	CG14935	+4999	CG14943	+3564	5	7	10	15	5.4		
146	PCE8209		3R	14,292,390	14,295,410	3,021	fru	+30861	fru	+30861	9	20	3	7	5.4		
147	PCE8210		3L	7,925,371	7,926,049	679	**exex**	+17651	RNaseX25	-4074	6	9	9	13	5.4		
148	PCE8212		3L	10,490,647	10,491,985	1,339	CG32062	+17114	CG32062	+17114	7	15	5	11	5.4		
149	PCE8214		2L	12,601,146	12,602,225	1,080	ref2	-895	CG15488	-433	8	11	7	10	5.4	**nub**	-6071
150	PCE8215		2L	1,262,811	1,263,598	788	robo3	+4320	robo3	+4320	7	9	9	11	5.4		
151	PCE8216		3L	2,124,879	2,125,504	626	CG11952	-2146	CG11953	+5214	6	8	10	13	5.4		
152	PCE8217		3L	6,427,171	6,428,277	1,107	CG14910	+5977	CG14911	-15304	8	11	7	10	5.3		
153	PCE8218		2L	10,545,226	10,547,197	1,972	**CG31721**	+7937	**CG31721**	+7937	10	14	5	7	5.3		
154	PCE8219		2L	20,565,501	20,566,968	1,468	CG15477	+51119	CG31677	+6970	8	14	5	10	5.3		
155	PCE8220		3L	11,285,342	11,286,077	736	CG6168	-6091	CG6163	+36340	7	8	10	11	5.3		
156	PCE8221		2R	7,617,876	7,619,748	1,873	fra	+24108	CG30056	-7049	7	18	4	10	5.3		
157	PCE8222		2L	5,405,877	5,406,553	677	H15	+9321	H15	+9321	7	7	10	10	5.3		
158	PCE8223		2R	4,530,951	4,532,251	1,301	CG13953	+12280	Camta	+142	9	11	7	8	5.3		
159	PCE8224		2L	13,051,685	13,052,501	817	CG9932	-31482	CG31856	+10214	5	12	6	15	5.3		
160	PCE8225		3R	18,797,064	18,797,775	712	CG17244	+3416	Or94a	-5883	6	9	8	13	5.3		
161	PCE8226		2L	12,541,433	12,542,145	713	**bun**	-11992	CG15489	-40512	6	9	8	13	5.2		
162	PCE8228		3R	22,644,179	22,644,773	595	Tl	+30050	Tl	+30050	6	7	10	12	5.2		
163	PCE8229		X	18,648,038	18,649,904	1,867	CG7378	+16671	CG32541	-11412	6	19	3	10	5.2		
164	PCE8230		3R	26,522,426	26,523,531	1,106	CG31010	-1245	CG1340	-17643	9	9	8	8	5.2		
165	PCE8231		3L	14,206,111	14,206,767	657	CG7906	+20508	fz	-16459	6	8	9	12	5.2		
166	PCE8232		3R	18,053,073	18,054,179	1,107	CG31163	+40454	CG31163	+40454	9	9	8	8	5.2		
167	PCE8233		2L	20,451,718	20,452,478	761	CG2493	-18527	CG15476	+19414	7	8	9	11	5.2		
168	PCE8234		2L	9,337,120	9,337,956	837	CG3748	+1891	CG13110	-4755	7	9	8	11	5.2		
169	PCE8235		X	2,190,216	2,191,697	1,482	**gt**	-4481	tko	+9051	9	12	6	8	5.2		
170	PCE8237		2L	12,670,755	12,671,417	663	**pdm2**	+3280	**pdm2**	+3280	6	8	9	12	5.2		
171	PCE8239		3R	1,972,623	1,973,690	1,068	CG1076	+24245	CG31563	-5208	8	10	7	9	5.2		
172	PCE8240		3R	12,696,513	12,697,212	700	**abd-A**	-40725	CG10349	-8252	7	7	10	10	5.1		
173	PCE8242		3L	6,855,809	6,857,382	1,574	vvl	+106121	Prat2	+16111	8	14	5	9	5.1		
174	PCE8243		2L	16,895,393	16,896,128	736	CG4841	+6632	beat-IIIb	-66610	6	9	8	12	5.1		
175	PCE8244		2R	6,785,122	6,787,280	2,159	CG13195	-17960	CG13194	-1355	10	14	5	6	5.1		
176	PCE8245		3L	6,438,623	6,439,396	774	CG14910	+17429	CG14911	-4185	7	8	9	10	5.1		
177	PCE8246		3L	7,838,003	7,839,579	1,577	CG32365	+9431	CG32365	+9431	6	17	4	11	5.1		
178	PCE8247		3R	15,388,862	15,389,498	637	Dys	+59333	CG7344	-5452	5	9	8	14	5.1		
179	PCE8248		2L	19,448,998	19,450,673	1,676	CG10034	+6154	CG10195	+12112	9	13	5	8	5.1		
180	PCE8249		3R	26,129,302	26,130,442	1,141	hdc	+36281	hdc	+36281	9	9	8	8	5.1		
181	PCE8251		3R	9,382,257	9,382,780	524	CG31337	-11132	CG14370	-31147	5	7	10	13	5.1		
182	PCE8252		X	7,380,962	7,382,647	1,686	ct	+8360	ct	+8360	7	16	4	9	5.1		
183	PCE8253		2L	18,416,318	18,417,309	992	CG31749	+51922	RpS26	+3903	6	12	6	12	5.1		
184	PCE8254		X	20,873,939	20,874,492	554	CG14579	+8229	CG1724	+1262	6	6	11	11	5.1		
185	PCE8255	ubx PBX	3R	12,598,962	12,599,746	785	**CG31275 (Ubx adj.)**	-21833	Glut3	-14304	7	8	9	10	5.1		
186	PCE8257		3L	11,233,121	11,234,915	1,795	scylla	+23281	CG32083	+3444	8	15	4	8	5.1		
187	PCE8258		3L	15,491,385	15,492,925	1,541	**CrebA**	+7093	**CrebA**	+7093	7	15	5	10	5.1		
188	PCE8259		3R	27,171,619	27,172,891	1,273	CG11339	+5461	CG11339	+5461	9	10	7	8	5.1		
189	PCE8260		3R	4,252,683	4,253,299	617	Obp85a	-2161	CG31369	+16263	6	7	10	11	5.1		

	CRM	Overlaps known element	Chrom arm	pCRM start	pCRM end	pCRM len	5' gene	pCRM relative position	3' gene	pCRM relative position	Aligned sites	Aligned + preserved sites	Aligned site dens	Aligned + preserved site dens	z-score	Additional Gap/pair-rule gene within 20kb	pCRM relative position
190	PCE8261		2R	8,190,874	8,191,590	717	CG13325	+9856	CG3955	+2178	7	7	10	10	5.1		
191	PCE8263		X	11,976,692	11,977,374	683	Ten-a	+16005	Ten-a	+16005	6	8	9	12	5.1		
192	PCE8264		3R	2,558,396	2,559,114	719	CG31481	-19647	zen2	+10749	7	7	10	10	5.1		
193	PCE8267		X	19,555,634	19,556,194	561	CG11942	-675	CG11940	+23219	6	6	11	11	5.0		
194	PCE8268		2L	12,139,561	12,140,905	1,345	CG31760	+7907	CG31760	+7907	6	15	4	11	5.0		
195	PCE8269		2L	1,835,449	1,836,676	1,228	CG15623	-1227	CG31665	-7987	8	11	7	9	5.0		
196	PCE8270		3L	16,421,730	16,422,846	1,117	**argos**	+9734	**argos**	+9734	8	10	7	9	5.0		
197	PCE8271		2R	14,861,230	14,861,852	623	Obp56g	-13497	Obp56h	-17437	6	7	10	11	5.0		
198	PCE8272		3R	24,056,635	24,057,259	625	CG12852	+3761	CG16918	-39531	6	7	10	11	5.0		
199	PCE8273		3L	13,607,859	13,609,095	1,237	bru-3	+15222	bru-3	+15222	8	11	6	9	5.0		
200	PCE8274		2R	7,902,167	7,902,858	692	CG17580	+30777	Cyp9h1	+4377	6	8	9	12	5.0		
201	PCE8275		3L	18,329,419	18,330,261	843	**grim**	-76126	**rpr**	+17021	6	10	7	12	5.0		
202	PCE8276		3R	26,479,187	26,480,315	1,129	CG15541	+14933	CG1342	-25416	8	10	7	9	5.0		
203	PCE8277		3R	6,448,750	6,449,993	1,244	**hth**	+8759	**hth**	+8759	6	14	5	11	5.0		
204	PCE8278		3R	25,283,631	25,284,260	630	Ptp99A	+6731	Ptp99A	+6731	6	7	10	11	5.0		
205	PCE8279		2R	15,932,538	15,933,268	731	otp	+7859	otp	+7859	5	10	7	14	5.0		
206	PCE8280		X	7,236,210	7,238,303	2,094	CG11368	+43626	CG32719	+14917	10	13	5	6	5.0		
207	PCE8281		2L	3,877,335	3,878,714	1,380	capu	+2679	capu	+2679	6	15	4	11	5.0		
208	PCE8282		2R	10,657,299	10,657,963	665	CG8204	-19583	CG30465	-12028	5	9	8	14	5.0		
209	PCE8283		2L	12,193,053	12,193,686	634	CG6579	-26296	aret	-1027	6	7	9	11	5.0		
210	PCE8284		3R	18,033,444	18,034,110	667	CG5732	+4935	CG31163	+60523	5	9	7	13	5.0		
211	PCE8285		2L	17,682,342	17,683,045	704	CadN	+34559	CadN	+34559	6	8	9	11	4.9		
212	PCE8286		3R	25,392,093	25,392,797	705	**Dr**	+20619	CG7567	+10391	6	8	9	11	4.9		
213	PCE8287		3R	27,129,709	27,130,860	1,152	CG11340	-9539	CG11339	-35298	8	10	7	9	4.9		
214	PCE8290		X	13,858,700	13,859,698	999	CG12454	+58500	CG32614	-3018	7	10	7	10	4.9		
215	PCE8292		2L	14,441,774	14,442,414	641	BG:DS08340.1	-8806	**noc**	-30599	6	7	9	11	4.9		
216	PCE8293		2L	5,932,279	5,932,988	710	dsf	+14128	Gpdh	-2908	6	8	8	11	4.9		
217	PCE8294		3R	11,438,518	11,440,000	1,483	CG18516	-60006	CG5302	-18622	5	17	3	11	4.9		
218	PCE8295		3L	16,111,912	16,113,071	1,160	CG5151	-14299	CG13073	+49908	6	13	5	11	4.9		
219	PCE8296		3R	8,984,274	8,985,768	1,495	timeout	+69883	timeout	+69883	9	11	6	7	4.9		
220	PCE8297		2R	20,280,374	20,281,018	645	**Kr**	+10190	CG30429	-9080	6	7	9	11	4.9		
221	PCE8298		3L	10,192,663	10,193,451	789	CG32056	-9894	Or67c	+8784	6	9	8	11	4.9		
222	PCE8300		X	7,141,490	7,142,043	554	CG1677	+4647	CG15035	+1508	5	7	9	13	4.9		
223	PCE8301		X	20,408,532	20,409,828	1,297	CG1324	-157	CG15452	+8536	6	14	5	11	4.9		
224	PCE8302		X	12,212,589	12,213,655	1,067	CG15926	+10262	CG2560	-3977	6	12	6	11	4.9		
225	PCE8304		2R	18,532,044	18,532,694	651	CG18678	+3454	CG13556	-711	6	7	9	11	4.9		
226	PCE8305		3R	24,029,896	24,030,267	372	CG12425	+67985	CG4787	-19919	4	5	11	13	4.9		
227	PCE8306		3L	12,278,550	12,279,346	797	CG4328	-28041	**CG32105**	-7436	6	9	8	11	4.9		
228	PCE8307		3L	5,580,997	5,581,649	653	CG12756	-13449	**CG5249**	-8641	6	7	9	11	4.9		
229	PCE8308		3L	6,976,211	6,978,322	2,112	CG33171	+2558	CG33171	+2558	9	14	4	7	4.9		
230	PCE8309		2L	3,825,809	3,827,419	1,611	**slp1**	+7561	**slp2**	-1991	8	13	5	8	4.9		
231	PCE8310		X	20,516,785	20,517,812	1,028	CG12678	-18024	CG1314	+24000	7	10	7	10	4.8		
232	PCE8311		3L	21,301,637	21,302,831	1,195	BcDNA:GH11973	+1838	BcDNA:GH11973	+1838	8	10	7	8	4.8		
233	PCE8312		3R	21,186,737	21,187,932	1,196	Fur1	+101406	CG11910	+11247	8	10	7	8	4.8		
234	PCE8314		2L	3,842,537	3,843,621	1,085	**slp2**	+13127	CG3964	-11628	6	12	6	11	4.8		
235	PCE8315		3L	918,940	920,143	1,204	Glut1	+20147	Glut1	+20147	8	10	7	8	4.8		
236	PCE8317		3L	14,832,855	14,834,580	1,726	CG17839	+78724	CG13467	-18412	9	12	5	7	4.8		
237	PCE8318		3R	13,029,447	13,030,254	808	CG4090	-43762	CG31262	+14915	6	9	7	11	4.8		
238	PCE8319		2L	11,240,184	11,241,449	1,266	ab	+37095	CG32830	-1995	7	12	6	9	4.8		
239	PCE8320		2L	2,004,674	2,005,266	593	CG15354	-49	CG15353	+3592	4	9	7	15	4.8		
240	PCE8321		3R	17,803,291	17,803,917	627	CG31353	-7506	Eip93F	+11606	5	8	8	13	4.8		
241	PCE8322		3R	8,658,121	8,658,685	565	CG31345	-15351	beat-Va	+10626	5	7	9	12	4.8		
242	PCE8323		3R	22,463,533	22,464,267	735	CG14243	+8421	CG14248	+2409	6	8	8	11	4.8		
243	PCE8324		2L	4,046,505	4,047,548	1,044	ed	+22558	ed	+22558	7	10	7	10	4.8		
244	PCE8325		2L	5,752,282	5,753,225	944	CG11030	+35410	CG11142	+5131	5	12	5	13	4.8		
245	PCE8326		X	20,410,563	20,411,780	1,218	CG1324	-2188	CG15452	+6584	8	10	7	8	4.8		
246	PCE8327		3R	17,959,744	17,960,442	699	CG31163	+9201	CG31163	+9201	5	9	7	13	4.8		
247	PCE8328		2L	16,418,533	16,419,580	1,048	**BG:DS02780.1**	+8016	Idgf1	-3783	7	10	7	10	4.8		
248	PCE8329		X	7,008,925	7,009,873	949	CG1958	+1645	CG1677	+16697	7	9	7	9	4.8		
249	PCE8330		3L	647,371	647,938	568	Rev1	+12382	CG17129	+12961	5	7	9	12	4.8		
250	PCE8331		3L	5,582,709	5,583,340	632	CG12756	-15161	**CG5249**	-6950	5	8	8	13	4.8		
251	PCE8332		3R	2,725,376	2,726,195	820	**Antp**	+32344	**Antp**	+32344	6	9	7	11	4.8		
252	PCE8333		3R	17,237,677	17,238,345	669	lbl	+12464	lbl	+12464	6	7	9	10	4.8		

	CRM	Overlaps known element	Chrom arm	pCRM start	pCRM end	pCRM len	5' gene	pCRM relative position	3' gene	pCRM relative position	Aligned sites	Aligned + preserved sites	Aligned site dens	Aligned + preserved site dens	z-score	Additional Gap/pair-rule gene within 20kb	pCRM relative position
253	PCE8334		2R	12,893,261	12,894,492	1,232	grh	+1837	grh	+1837	8	10	6	8	4.8		
254	PCE8335		3L	14,237,854	14,239,634	1,781	fz	+14600	fz	+14600	9	12	5	7	4.8		
255	PCE8336		3R	15,506,370	15,507,007	638	CG31216	+9171	CG31216	+9171	5	8	8	13	4.7		
256	PCE8337		2R	17,333,048	17,334,170	1,123	dve	+45535	CG5819	-2839	6	12	5	11	4.7		
257	PCE8338		3R	3,987,824	3,989,532	1,709	**grn**	+17647	**grn**	+17647	8	13	5	8	4.7		
258	PCE8339		3R	8,778,516	8,780,328	1,813	BcDNA:LD41548	+7048	BcDNA:LD41548	+7048	9	12	5	7	4.7		
259	PCE8340		3L	4,840,194	4,841,268	1,075	CG17150	+794	CG17150	+794	7	10	7	9	4.7		
260	PCE8341		3L	13,561,767	13,562,378	612	bru-3	+61939	bru-3	+61939	6	6	10	10	4.7		
261	PCE8342		3L	7,797,257	7,798,139	883	Pdp1	+4655	Pdp1	+4655	7	8	8	9	4.7		
262	PCE8343		2L	13,598,988	13,600,068	1,081	kuz	+66697	kuz	+66697	7	10	6	9	4.7		
263	PCE8344		2L	9,054,174	9,054,891	718	CG31708	+8502	CG31708	+8502	5	9	7	13	4.7		
264	PCE8345		2L	16,619,155	16,620,782	1,628	CG31738	+14416	CG31738	+14416	5	17	3	10	4.7		
265	PCE8346		2L	12,569,943	12,571,337	1,395	**bun**	-40502	CG15489	-11320	6	14	4	10	4.7		
266	PCE8347		X	7,198,428	7,200,261	1,834	CG11368	+5844	CG32719	+52959	9	12	5	7	4.7		
267	PCE8348		3L	18,966,181	18,967,380	1,200	**nkd**	+26830	**nkd**	+26830	7	11	6	9	4.7		
268	PCE8349		X	8,505,435	8,506,838	1,404	Lim1	+37936	Lim1	+37936	8	11	6	8	4.7		
269	PCE8350		3L	2,812,397	2,813,478	1,082	CG2083	-31638	CG14952	+15046	5	13	5	12	4.7		
270	PCE8351		2L	19,619,536	19,620,568	1,033	Lar	+30044	Lar	+30044	6	11	6	11	4.7		
271	PCE8352		2L	14,836,647	14,837,731	1,085	BG:DS03431.1	-6814	CG4480	+12375	5	13	5	12	4.7		
272	PCE8353		3L	408,630	409,181	552	trh	-50684	CG13891	+5339	4	8	7	14	4.7		
273	PCE8354		2L	7,266,538	7,267,472	935	Wnt4	+1927	Wnt4	+1927	6	10	6	11	4.7		
274	PCE8355		3R	6,421,647	6,422,583	937	**hth**	+8827	**hth**	+8827	6	10	6	11	4.7		
275	PCE8356		3L	22,244,275	22,244,894	620	**Ten-m**	+80890	CG32450	-2161	6	6	10	10	4.7		
276	PCE8357		3R	13,066,783	13,068,448	1,666	beat-IIb	+5165	beat-IIb	+5165	9	11	5	7	4.7		
277	PCE8358		3R	26,740,914	26,742,495	1,582	**Ptx1**	+2496	**Ptx1**	+2496	8	12	5	8	4.7		
278	PCE8359		3R	3,961,949	3,962,994	1,046	CG7891	+9599	**grn**	+44185	8	8	8	8	4.7		
279	PCE8360		3R	11,566,576	11,567,198	623	CG31461	+20324	CG4520	-3195	6	6	10	10	4.6		
280	PCE8361	Ubx BRE	3R	12,526,665	12,527,949	1,285	**Ubx**	+32417	**Ubx**	+32417	6	13	5	10	4.6		
281	PCE8362		X	670,447	671,138	692	CG11663	+18529	EG:BACR7A4.19	+13862	4	10	6	14	4.6		
282	PCE8363		3R	8,168,121	8,170,217	2,097	CG10013	+18858	CG10038	+6133	5	19	2	9	4.6		
283	PCE8364		2L	3,951,733	3,952,895	1,163	fred	-12004	CG15422	-44729	6	12	5	10	4.6		
284	PCE8365		3R	13,929,707	13,930,440	734	sr	+13168	sr	+13168	5	9	7	12	4.6		
285	PCE8367		2R	4,771,288	4,771,881	594	CG10459	+3018	**dap**	-1074	5	7	8	12	4.6		
286	PCE8368		3R	16,057,342	16,058,157	816	CG31209	+6389	CG31209	+6389	5	10	6	12	4.6		
287	PCE8369		3L	14,540,753	14,541,382	630	**HGTX**	+7066	**HGTX**	+7066	6	6	10	10	4.6		
288	PCE8370		3L	2,395,158	2,396,393	1,236	CG13800	+12412	**CG32306**	-13538	5	14	4	11	4.6		
289	PCE8371		3L	6,879,258	6,879,960	703	Prat2	-4871	CG14820	-12498	6	7	9	10	4.6		
290	PCE8373		3R	13,397,033	13,398,583	1,551	CG18139	+3343	CG7587	-21576	9	10	6	6	4.6		
291	PCE8376		3R	21,352,808	21,354,366	1,559	msi	+19399	msi	+19399	7	13	4	8	4.6		
292	PCE8377		X	14,416,809	14,417,729	921	CG5321	+6191	**NetB**	+62559	5	11	5	12	4.6		
293	PCE8378		3L	5,114,119	5,116,458	2,340	Srp54k	+2242	**CG32423**	+27644	8	15	3	6	4.6		
294	PCE8379		2R	19,431,133	19,431,614	482	mAcR-60C	+1666	mAcR-60C	+1666	5	5	10	10	4.6		
295	PCE8380		3R	9,636,667	9,638,244	1,578	CG14363	-19947	Mst87F	+15207	9	10	6	6	4.5		
296	PCE8381		3L	2,143,526	2,144,132	607	CG15822	+357	CG15822	+357	5	7	8	12	4.5		
297	PCE8382		2L	12,271,853	12,272,460	608	aret	+3635	aret	+3635	5	7	8	12	4.5		
298	PCE8383		3R	11,464,046	11,464,759	714	CG5302	+5424	CG5302	+5424	4	10	6	14	4.5		
299	PCE8385		3R	13,941,480	13,942,158	679	sr	+5776	sr	+5776	5	8	7	12	4.5		
300	PCE8386		2L	18,751,913	18,752,630	718	CG31753	+1827	CG31753	+1827	6	7	8	10	4.5		
301	PCE8388		3L	1,256,948	1,257,837	890	CG32333	+348	CG32333	+348	6	9	7	10	4.5		
302	PCE8389		3R	22,312,308	22,312,955	648	CG6490	-19138	scrib	-38477	6	6	9	9	4.5		
303	PCE8390		3L	1,902,800	1,903,522	723	CG13925	+757	CG12361	-1426	6	7	8	10	4.5		
304	PCE8391		3L	5,254,002	5,254,895	894	**CG32423**	-16750	lama	+55892	6	9	7	10	4.5		
305	PCE8392		2L	18,320,177	18,321,340	1,164	Fas3	+21994	Fas3	+21994	7	10	6	9	4.5		
306	PCE8393		2L	4,084,125	4,084,704	580	ed	+60178	ed	+60178	4	8	7	14	4.5		
307	PCE8394	Kr 730	2R	20,266,323	20,267,047	725	CG9380	-33916	**Kr**	-3137	6	7	8	10	4.5		
308	PCE8395		3R	10,157,629	10,158,244	616	spn-B	+3825	NK7.1	+34788	5	7	8	11	4.5		
309	PCE8396		X	7,288,765	7,290,792	2,028	CG32720	-11499	CG11369	-24182	7	15	3	7	4.5		
310	PCE8397		3R	11,505,261	11,506,493	1,233	pxb	+13336	pxb	-4216	6	12	5	10	4.5		
311	PCE8398		3R	2,770,846	2,771,901	1,056	**Antp**	+12307	**Antp**	+12307	7	9	7	9	4.5		
312	PCE8399		X	20,268,266	20,269,376	1,111	CG15453	-1554	CG1379	+8904	6	11	5	10	4.5		
313	PCE8401		2L	12,660,502	12,661,614	1,113	CG15485	-2463	**pdm2**	+5861	6	11	5	10	4.5		
314	PCE8402		3L	18,077,103	18,078,164	1,062	CG7341	+15260	Cyp312a1	+2781	7	9	7	8	4.5		
315	PCE8403		3R	25,087,655	25,088,209	555	stg	-16787	CG14506	+24108	5	6	9	11	4.5		

	CRM	Overlaps known element	Chrom arm	pCRM start	pCRM end	pCRM len	5' gene	pCRM relative position	3' gene	pCRM relative position	Aligned sites	Aligned + preserved sites	Aligned site dens	Aligned + preserved site dens	z-score	Additional Gap/pair-rule gene within 20kb	pCRM relative position
316	PCE8404		X	19,558,673	19,559,489	817	CG11942	-3714	CG11940	+19924	6	8	7	10	4.5		
317	PCE8405		3L	1,109,402	1,109,990	589	CG32336	-9787	bab2	+47913	4	8	7	14	4.4		
318	PCE8406		3R	23,650,127	23,651,382	1,256	CG5566	-921	Or98a	-9654	6	12	5	10	4.4		
319	PCE8407		2L	11,341,949	11,342,574	626	CG14926	-9815	salr	-11601	5	7	8	11	4.4		
320	PCE8408		X	8,379,690	8,381,014	1,325	**oc**	+8582	**oc**	+8582	5	14	4	11	4.4		
321	PCE8409		2L	21,908,048	21,908,515	468	CG31702	+3820	CG6691	-4232	4	6	9	13	4.4		
322	PCE8410		2L	1,581,371	1,582,072	702	CG14351	+24547	CG10869	+6864	5	8	7	11	4.4		
323	PCE8411		3R	17,913,658	17,914,400	743	CG5791	+9840	CG13407	-5699	6	7	8	9	4.4		
324	PCE8412		X	11,994,565	11,996,148	1,584	Ten-a	+33878	Ten-a	+33878	8	11	5	7	4.4		
325	PCE8413		X	14,942,402	14,943,324	923	CG5662	+991	CG15030	-3478	6	9	7	10	4.4		
326	PCE8415		3R	13,867,601	13,868,164	564	CG7794	+18158	**htl**	+6934	5	6	9	11	4.4		
327	PCE8416		X	7,122,644	7,123,928	1,285	CG15033	+4005	CG1677	+22209	8	9	6	7	4.4		
328	PCE8417		2L	587,804	588,638	835	**Gsc**	+7714	**Gsc**	+7714	6	8	7	10	4.4		
329	PCE8418		3R	18,950,000	18,950,634	635	CG31457	-5638	**hh**	+7739	5	7	8	11	4.4	**cenB1A**	12397
330	PCE8419		2R	5,453,237	5,454,330	1,094	CG12899	-4190	CG12898	+13748	7	9	6	8	4.4		
331	PCE8420		2L	8,641,383	8,642,092	710	Sema-1a	+85357	Sema-1a	+85357	5	8	7	11	4.4		
332	PCE8421		3L	13,240,137	13,240,704	568	caps	+53902	Acp70A	-18498	5	6	9	11	4.4		
333	PCE8422		3R	17,657,890	17,658,819	930	CG13416	+300	Gr93b	+1650	4	12	4	13	4.4		
334	PCE8423		3L	15,970,589	15,971,950	1,362	CG5841	-9898	Notum	-6316	7	11	5	8	4.4		
335	PCE8424		3R	8,070,353	8,071,965	1,613	Cyp313a3	-5503	CG3942	-7962	8	11	5	7	4.4		
336	PCE8425		2R	18,693,096	18,694,318	1,223	**retn**	+16917	CG5411	-6825	7	10	6	8	4.4		
337	PCE8426		2L	1,268,149	1,268,904	756	robo3	+9658	robo3	+9658	6	7	8	9	4.4		
338	PCE8427		3R	9,625,048	9,625,469	422	CG14363	-8328	Mst87F	+27982	4	5	9	12	4.4		
339	PCE8428	CE8013	2L	13,989,283	13,990,133	851	rk	-5879	bgm	-5766	6	8	7	9	4.3		
340	PCE8429		X	9,756,644	9,758,386	1,743	CG2990	+6234	CG2989	-11633	7	13	4	7	4.3		
341	PCE8430		3L	10,258,913	10,259,595	683	CG6559	+10647	CG6559	+10647	4	9	6	13	4.3		
342	PCE8431		2L	16,508,455	16,510,102	1,648	CG5953	+859	CG5953	+859	6	14	4	8	4.3		
343	PCE8432		2L	21,728,314	21,729,432	1,119	**tsh**	+66599	CG11629	+1950	5	12	4	11	4.3		
344	PCE8433		2L	15,966,384	15,967,151	768	BG:DS07486.2	+8741	beat-Ic	-11469	6	7	8	9	4.3		
345	PCE8434		3R	23,863,568	23,864,216	649	CG12870	+907	CG12870	+907	5	7	8	11	4.3		
346	PCE8435		2L	5,445,679	5,446,492	814	CG31647	-4262	CG6634	-7363	5	9	6	11	4.3		
347	PCE8436		3L	9,967,152	9,967,845	694	CG14162	+35005	CG14162	+35005	6	6	9	9	4.3		
348	PCE8437		2L	893,144	893,838	695	CG15824	-6427	CG11297	-3055	6	6	9	9	4.3		
349	PCE8438		2L	11,536,884	11,537,539	656	CG31706	-16964	Mst33A	+37946	5	7	8	11	4.3		
350	PCE8439		X	4,770,587	4,771,859	1,273	CG12680	+32240	**ovo**	-17051	7	10	5	8	4.3		
351	PCE8440		3L	11,295,694	11,296,961	1,268	CG6168	-16443	CG6163	+25456	5	13	4	10	4.3		
352	PCE8441		2R	8,434,804	8,435,354	551	CG17048	-15757	CG10814	+7074	4	7	7	13	4.3		
353	PCE8442		X	8,521,716	8,522,921	1,206	Lim1	+21853	Lim1	+21853	6	11	5	9	4.3		
354	PCE8443		2L	20,456,074	20,457,355	1,282	CG2493	-22883	CG15476	+14537	7	10	5	8	4.3		
355	PCE8444		3L	18,330,763	18,332,045	1,283	**grim**	-77470	**rpr**	+15237	7	10	5	8	4.3		
356	PCE8445		3R	9,353,080	9,354,054	975	CG12538	-3804	CG31337	+17071	6	9	6	9	4.3		
357	PCE8446		3R	22,428,048	22,429,201	1,154	CG5467	+15290	CG5468	-5008	7	9	6	8	4.3		
358	PCE8447		3R	17,401,939	17,403,156	1,218	InR	-4724	**E2f**	+46552	6	11	5	9	4.3		
359	PCE8448		X	7,394,857	7,395,556	700	ct	+22255	ct	+22255	4	9	6	13	4.3		
360	PCE8449		3R	9,269,352	9,270,575	1,224	CG9764	-8629	CG14372	-11080	8	8	7	7	4.3		
361	PCE8450		3L	5,141,131	5,141,793	663	**CG32423**	+2971	CG10677	-438	5	7	8	11	4.3		
362	PCE8451		2L	15,947,581	15,948,244	664	CG31736	+1080	BG:DS07486.2	-9399	5	7	8	11	4.2		
363	PCE8452		2L	16,567,799	16,568,462	664	CG13260	+31587	CG31815	-11534	5	7	8	11	4.2		
364	PCE8453		3R	24,035,108	24,036,202	1,095	CG12425	+73197	CG4787	-13984	6	10	5	9	4.2		
365	PCE8454		2L	2,999,166	2,999,722	557	BEST:LD23852	+13634	CG17265	-6347	4	7	7	13	4.2		
366	PCE8455		3R	2,388,846	2,389,510	665	CG1137	-2772	CG33202	+29543	5	7	8	11	4.2		
367	PCE8456		2L	9,059,953	9,060,882	930	CG31708	+2511	CG31708	+2511	5	10	5	11	4.2		
368	PCE8458		3L	19,101,833	19,102,666	834	**fz2**	+6194	**fz2**	+6194	5	9	6	11	4.2		
369	PCE8459		3R	9,444,257	9,444,783	527	CG9269	+2513	CG14368	+6587	5	5	9	9	4.2		
370	PCE8460		2L	9,962,785	9,964,166	1,382	RpL13	+3065	bib	-12889	8	9	6	7	4.2		
371	PCE8461		X	13,691,125	13,692,755	1,631	CG11068	+5216	CG32613	+5235	5	15	3	9	4.2		
372	PCE8462		2L	11,578,719	11,579,821	1,103	Mst33A	-3015	CG32952	+730	6	10	5	9	4.2		
373	PCE8463		3L	7,777,464	7,778,568	1,105	Pdp1	+7547	Pdp1	+7547	6	10	5	9	4.2		
374	PCE8464		3L	17,314,105	17,314,815	711	**tap**	+5577	**Cad74A**	+13577	6	6	8	8	4.2		
375	PCE8465		2R	17,318,862	17,320,846	1,985	dve	+31349	dve	+31349	8	12	4	6	4.2		
376	PCE8466		3L	4,137,270	4,137,983	714	CG15000	-148	CG15001	+3292	6	6	8	8	4.2		
377	PCE8467		X	19,624,303	19,625,098	796	CG32529	+1156	CG32529	+1156	4	10	5	13	4.2		
378	PCE8468		3R	24,305,523	24,305,962	440	Dhc98D	+3483	Dhc98D	+3483	4	5	9	11	4.2		

	CRM	Overlaps known element	Chrom arm	pCRM start	pCRM end	pCRM len	5' gene	pCRM relative position	3' gene	pCRM relative position	Aligned sites	Aligned + preserved sites	Aligned site dens	Aligned + preserved site dens	z-score	Additional Gap/pair-rule gene within 20kb	pCRM relative position
379	PCE8469		3R	9,771,895	9,772,496	602	CG9927	-372	BcDNA:LD34214	-1679	5	6	8	10	4.2		
380	PCE8470	CE8014	2L	18,400,276	18,401,850	1,575	Fas3	+102093	RpS26	+19362	6	13	4	8	4.2		
381	PCE8471		3R	9,696,260	9,697,381	1,122	E5	+2539	E5	+2539	6	10	5	9	4.2		
382	PCE8472		2L	12,563,964	12,564,680	717	**bun**	-34523	CG15489	-17977	4	9	6	13	4.2		
383	PCE8473		3R	26,758,899	26,759,618	720	CG15550	-6713	CG15548	-15276	6	6	8	8	4.2		
384	PCE8474		2R	16,929,770	16,930,373	604	PpN58A	-3336	CG4054	-42697	5	6	8	10	4.2		
385	PCE8475		3L	13,564,776	13,566,272	1,497	bru-3	+58045	bru-3	+58045	7	11	5	7	4.2		
386	PCE8476		2L	2,611,713	2,612,394	682	CG15395	+9001	CG9962	+27818	5	7	7	10	4.2		
387	PCE8477		2L	12,886,108	12,886,791	684	kek1	-74451	ACXC	-14608	5	7	7	10	4.2		
388	PCE8478		3R	14,587,862	14,588,629	768	CG14299	-3257	CG31227	+9876	5	8	7	10	4.1		
389	PCE8479		3L	12,644,990	12,646,008	1,019	CG32111	+59508	mirr	-5286	6	9	6	9	4.1		
390	PCE8480		X	13,596,848	13,598,453	1,606	CG11071	-9065	CG32611	+17718	6	13	4	8	4.1		
391	PCE8481		3L	18,208,922	18,209,999	1,078	CG7313	+7947	CG5103	+33247	5	11	5	10	4.1		
392	PCE8483		2L	8,265,854	8,267,283	1,430	**Btk29A**	+2646	**Btk29A**	+2646	4	15	3	10	4.1		
393	PCE8484		3L	7,158,937	7,160,467	1,531	CG32387	-15506	CG14826	-33845	7	11	5	7	4.1		
394	PCE8485		3L	5,784,325	5,785,412	1,088	vn	+19510	vn	+19510	7	8	6	7	4.1		
395	PCE8486		3L	5,444,803	5,446,015	1,213	CG4835	+5360	CG10630	+12223	5	12	4	10	4.1		
396	PCE8487		3R	24,227,355	24,228,443	1,089	Or98b	+60026	beat-VI	-4848	7	8	6	7	4.1		
397	PCE8488		X	211,076	211,586	511	pcl	-16196	ase	-6351	4	6	8	12	4.1		
398	PCE8489		2L	17,702,171	17,703,204	1,034	CadN	+14400	CadN	+14400	6	9	6	9	4.1		
399	PCE8490		3R	17,791,038	17,791,617	580	CG31171	-41532	Eip93F	-694	4	7	7	12	4.1		
400	PCE8491		3L	2,162,230	2,163,458	1,229	CG15820	-7269	CG13810	-3167	7	9	6	7	4.1		
401	PCE8492		2L	17,689,744	17,690,224	481	CadN	+27380	CadN	+27380	3	7	6	15	4.1		
402	PCE8493		3R	6,403,852	6,405,604	1,753	**hth**	+25806	**hth**	+25806	7	12	4	7	4.1		
403	PCE8494		3R	7,931,641	7,932,680	1,040	CG31361	+7815	**CG31361**	+7815	6	9	6	9	4.1		
404	PCE8495		2L	5,214,677	5,215,845	1,169	CG6514	+3847	**tkv**	+14084	6	10	5	9	4.1		
405	PCE8497		3L	3,386,043	3,387,280	1,238	sty	+11276	sty	+11276	5	12	4	10	4.1		
406	PCE8498		3R	13,968,428	13,969,741	1,314	sr	+20494	CG14316	-8115	6	11	5	8	4.1		
407	PCE8499		2R	10,661,967	10,662,848	882	CG8204	-24251	CG30465	-7143	5	9	6	10	4.1		
408	PCE8500		X	6,969,321	6,970,022	702	CG9650	+5106	CG9650	+5106	5	7	7	10	4.1		
409	PCE8501		2L	5,247,719	5,248,767	1,049	**tkv**	+10898	Cyp4ac1	-7804	6	9	6	9	4.1		
410	PCE8502		3L	5,346,875	5,347,536	662	CG10633	-4746	CG4814	-44778	4	8	6	12	4.1		
411	PCE8503		X	7,105,113	7,106,001	889	CG1999	+5941	CG1677	+40136	5	9	6	10	4.1		
412	PCE8504		2R	14,950,058	14,951,645	1,588	CG13872	-5425	CG30447	-59383	7	11	4	7	4.1		
413	PCE8505		3L	9,077,635	9,078,190	556	bol	+5815	bol	+5815	5	5	9	9	4.1		
414	PCE8507		X	7,100,782	7,101,787	1,006	CG1999	+1610	CG1677	+44350	7	7	7	7	4.0		
415	PCE8509		2L	2,494,167	2,495,118	952	oaf	+8646	CG3528	+15486	6	8	6	8	4.0		
416	PCE8510		2L	14,866,153	14,866,865	713	Mst35Bb	-4354	CG15277	+20986	5	7	7	10	4.0		
417	PCE8511		3R	6,469,170	6,470,599	1,430	**hth**	-4766	CG6465	+32311	7	10	5	7	4.0		
418	PCE8512	pdm2 neurogenic	2L	12,663,453	12,664,721	1,269	**pdm2**	+2754	**pdm2**	+2754	5	12	4	9	4.0		
419	PCE8513		3L	14,550,945	14,551,746	802	**HGTX**	-2497	Cyp314a1	-16963	5	8	6	10	4.0		
420	PCE8514		2L	20,469,960	20,470,764	805	CG2493	-36769	CG15476	+1128	5	8	6	10	4.0		
421	PCE8515		2L	16,390,610	16,392,235	1,626	**BG:DS02780.1**	+34314	**BG:DS02780.1**	+34314	7	11	4	7	4.0		
422	PCE8516		2R	17,095,726	17,096,802	1,077	PpD5	-9293	CG13500	+4430	6	9	6	8	4.0		
423	PCE8517		3L	11,326,031	11,327,309	1,279	CG6163	-3614	CG11726	-24993	5	12	4	9	4.0		
424	PCE8518		3L	7,224,680	7,225,208	529	CG14830	-1234	unc-13-4A	+19308	4	6	8	11	4.0		
425	PCE8519		3L	8,975,309	8,975,873	565	**Doc2**	+2077	**Doc2**	+2077	5	5	9	9	4.0	**Doc3**	11402
426	PCE8520		2L	12,080,772	12,081,448	677	**prd**	-5445	CG5325	-1193	4	8	6	12	4.0		
427	PCE8521		2L	7,252,370	7,253,008	639	CG31909	+2569	Wnt4	+16391	5	6	8	9	4.0	**Ndae1**	-19639
428	PCE8522		3R	15,325,630	15,326,485	856	Dys	+3044	Dys	+3044	4	10	5	12	4.0		
429	PCE8523		3R	13,942,711	13,943,621	911	sr	+4313	sr	+4313	5	9	5	10	4.0		
430	PCE8524		3L	7,144,287	7,145,055	769	CG32387	-856	CG14826	-49257	6	6	8	8	4.0		
431	PCE8525		2L	16,786,999	16,787,766	768	Ugt36Bc	+9887	CG13272	-1982	4	9	5	12	4.0		
432	PCE8527		2L	3,949,933	3,951,233	1,301	fred	-10204	CG15422	-46391	5	12	4	9	4.0		
433	PCE8528		X	14,366,706	14,367,311	606	**NetA**	+17535	**NetA**	+17535	4	7	7	12	4.0		
434	PCE8529		2R	13,008,426	13,009,734	1,309	elk	+70755	PpY-55A	+10561	7	9	5	7	4.0		
435	PCE8530		X	12,580,305	12,581,227	923	CG3754	-18007	CG4661	+7157	5	9	5	10	4.0		
436	PCE8531		3R	6,363,866	6,364,968	1,103	CG31394	-8970	**hth**	+66442	6	9	5	8	4.0		
437	PCE8532		2L	1,404,981	1,405,630	650	lea	+16485	lea	+16485	5	6	8	9	3.9		
438	PCE8533		3R	24,402,963	24,403,946	984	**fkh**	-2792	Noa36	+10421	6	8	6	8	3.9		
439	PCE8534		3R	18,095,882	18,096,534	653	CG31163	+1249	CG31163	+1249	5	6	8	9	3.9		
440	PCE8535		3R	2,859,299	2,860,288	990	**Antp**	-33021	Sodh-1	-17769	6	8	6	8	3.9		
441	PCE8536		3R	12,764,472	12,765,970	1,499	**Abd-B**	+4036	**Abd-B**	+4036	7	10	5	7	3.9		

	CRM	Overlaps known element	Chrom arm	pCRM start	pCRM end	pCRM len	5' gene	pCRM relative position	3' gene	pCRM relative position	Aligned sites	Aligned + preserved sites	Aligned site dens	Aligned + preserved site dens	z-score	Additional Gap/pair-rule gene within 20kb	pCRM relative position
442	PCE8537		X	10,304,767	10,305,879	1,113	spri	-17771	CG15296	+880	6	9	5	8	3.9		
443	PCE8538		3L	14,319,615	14,320,496	882	CG13481	-1092	CG3868	+31103	6	7	7	8	3.9		
444	PCE8539		2R	10,093,650	10,094,587	938	Obp51a	-5085	hbs	+19464	7	6	7	6	3.9		
445	PCE8540		2L	16,605,006	16,605,888	883	CG31738	+4879	CG31738	+4879	6	7	7	8	3.9		
446	PCE8541		2L	2,682,494	2,683,612	1,119	CG31690	+15103	CG15398	+5275	6	9	5	8	3.9		
447	PCE8542		3L	17,960,347	17,960,963	617	Eip75B	+11647	Eip75B	+11647	4	7	6	11	3.9		
448	PCE8543		X	7,088,709	7,089,648	940	CG15034	-838	CG1999	-9524	5	9	5	10	3.9		
449	PCE8544		2R	10,089,704	10,091,422	1,719	Obp51a	-1139	hbs	+16299	7	11	4	6	3.9		
450	PCE8545		2L	19,581,175	19,581,755	581	CG10366	+18834	Lar	-7737	3	8	5	14	3.9		
451	PCE8546		3L	4,674,003	4,674,586	584	CG15876	-1323	CG13713	-1033	5	5	9	9	3.9		
452	PCE8547		X	3,111,977	3,112,560	584	dnc	+19670	**dm**	-19060	5	5	9	9	3.9		
453	PCE8548		3R	4,509,837	4,510,965	1,129	CG11755	+5816	**hb**	+9372	6	9	5	8	3.9		
454	PCE8549		3R	26,836,195	26,837,262	1,068	5-HT7	-4260	CanA1	-17201	7	7	7	7	3.9		
455	PCE8550		3L	12,339,988	12,340,734	747	CG4357	+8228	CG4357	+8228	5	7	7	9	3.9		
456	PCE8552		2L	19,612,876	19,613,719	844	Lar	+23384	Lar	+23384	5	8	6	9	3.9		
457	PCE8553		2R	16,289,218	16,289,840	623	**CG18375**	+4664	**CG18375**	+4664	4	7	6	11	3.9		
458	PCE8554		3R	4,051,838	4,052,844	1,007	CG18249	+5933	DNApol-iota	-6399	4	11	4	11	3.9		
459	PCE8555		3L	3,972,362	3,972,911	550	scrt	+7818	CG14985	-11189	4	6	7	11	3.9		
460	PCE8556		3R	18,054,746	18,055,816	1,071	CG31163	+38817	CG31163	+38817	5	10	5	9	3.9		
461	PCE8557		3L	10,286,478	10,288,336	1,859	CG6559	-16236	CG12362	-82008	6	13	3	7	3.9		
462	PCE8558		3R	26,927,721	26,928,386	666	CG11318	-3445	Prosalpha3T	+10083	5	6	8	9	3.9		
463	PCE8559		3R	26,492,980	26,494,633	1,654	CG15541	+28726	CG1342	-11098	8	9	5	5	3.9		
464	PCE8560		2R	9,444,567	9,445,584	1,018	CG8422	+2717	CG8422	+2717	6	8	6	8	3.9		
465	PCE8561	CE8023	3R	2,594,997	2,596,453	1,457	**Ama**	+5869	**Dfd**	-21105	6	11	4	8	3.9	**bcd**	-9797
466	PCE8563		2R	9,986,845	9,988,405	1,561	BcDNA:GH03482	-10902	CG10253	+8621	7	10	4	6	3.9		
467	PCE8564		X	9,441,828	9,443,049	1,222	**btd**	+8657	Sp1	-48893	5	11	4	9	3.8		
468	PCE8565		2L	17,262,387	17,263,351	965	beat-IIIc	-23572	CG6380	+6939	5	9	5	9	3.8		
469	PCE8566		3L	9,279,858	9,280,823	966	Glu-RIB	+68725	PGRP-LA	-12219	5	9	5	9	3.8		
470	PCE8567		3L	22,362,130	22,362,803	674	CG14460	+20934	CG11449	-8847	5	6	7	9	3.8		
471	PCE8568		2L	9,811,200	9,812,771	1,572	nAcRalpha-30D	+65672	nAcRalpha-30D	+65672	7	10	4	6	3.8		
472	PCE8569		2L	18,312,711	18,313,740	1,030	Fas3	+14528	Fas3	+14528	6	8	6	8	3.8		
473	PCE8570		2L	8,797,367	8,797,855	489	CG9468	-23885	**SoxN**	-20000	4	5	8	10	3.8		
474	PCE8572		2R	12,378,676	12,379,906	1,231	CG33197	+49399	CG33197	+49399	5	11	4	9	3.8		
475	PCE8573		3R	6,374,489	6,376,060	1,572	**hth**	+55350	**hth**	+55350	5	13	3	8	3.8		
476	PCE8574		X	2,666,269	2,667,300	1,032	EG:BACR43E12.4	+16263	EG:100G7.6	-9240	6	8	6	8	3.8		
477	PCE8575		2R	8,372,946	8,374,434	1,489	CG17047	+8578	CG17048	+44613	8	8	5	5	3.8		
478	PCE8576		3R	12,596,022	12,597,333	1,312	CG31275	-18893	Glut3	-16717	6	10	5	8	3.8		
479	PCE8577		3R	12,568,810	12,569,623	814	**Ubx**	-8444	CG31275	+7451	6	6	7	7	3.8		
480	PCE8578		2L	10,564,075	10,564,501	427	CG6138	+4377	CG6138	+4377	4	4	9	9	3.8		
481	PCE8579		2L	2,997,676	2,998,490	815	BEST:LD23852	+12144	CG17265	-7579	6	6	7	7	3.8		
482	PCE8580		2L	10,512,991	10,513,854	864	Lrr47	+3246	CG6113	-7971	5	8	6	9	3.8		
483	PCE8581		2L	16,355,350	16,356,517	1,168	BG:DS09218.5	+11348	**BG:DS02780.1**	+70032	6	9	5	8	3.8		
484	PCE8582		2L	1,929,433	1,930,069	637	CG7337	+17861	CG7337	+17861	4	7	6	11	3.8		
485	PCE8583		2L	11,452,927	11,453,606	680	**salm**	-18453	**sala**	-21453	5	6	7	9	3.8		
486	PCE8584		3R	6,351,140	6,351,819	680	**hth**	+80270	CG31394	+3077	5	6	7	9	3.8		
487	PCE8585		2R	18,154,189	18,155,793	1,605	Gr59d	-5452	Klp59C	+13817	7	10	4	6	3.8		
488	PCE8586		3R	3,124,600	3,125,781	1,182	rn	+9468	CG32467	+2132	6	9	5	8	3.8		
489	PCE8587		3L	2,821,674	2,822,603	930	CG2083	-40915	CG14952	+5921	4	10	4	11	3.8		
490	PCE8588		X	15,857,208	15,857,812	605	disco-r	-2995	**disco**	+89941	3	8	5	13	3.8		
491	PCE8589		2R	7,087,153	7,087,842	690	otk	-2616	CG8964	+2141	5	6	7	9	3.8		
492	PCE8590		2L	1,702,114	1,703,457	1,344	CG31666	+32603	cpb	-3111	4	13	3	10	3.8		
493	PCE8591		3R	17,331,272	17,332,469	1,198	C15	+14332	CG7956	-5157	6	9	5	8	3.8		
494	PCE8592		2L	3,852,586	3,853,784	1,199	**slp2**	+23176	CG3964	-1465	6	9	5	8	3.8		
495	PCE8593		3L	474,796	475,488	693	klar	+45730	klar	+45730	5	6	7	9	3.8		
496	PCE8594		2L	5,555,971	5,557,603	1,633	Glu-RIIB	+4415	CG14011	+15728	5	13	3	8	3.8		
497	PCE8595		3R	23,233,906	23,234,406	501	side	-294	CG13978	-44869	4	5	8	10	3.8		
498	PCE8596		3L	14,869,258	14,870,201	944	CG13467	+16266	CG13466	-378	6	7	6	7	3.8		
499	PCE8597		2L	12,612,518	12,613,584	1,067	**nub**	+5301	**nub**	+5301	6	8	6	7	3.8		
500	PCE8598		2L	7,326,567	7,327,139	573	**wg**	+25247	Wnt6	-15989	4	6	7	10	3.8		
501	PCE8599		X	2,568,404	2,569,406	1,003	EG:BACR43E12.1	+1666	EG:BACR43E12.7	+60186	5	9	5	9	3.8		
502	PCE8600		3L	10,765,390	10,766,595	1,206	CG12523	+6670	CG7958	-48825	6	9	5	7	3.7		
503	PCE8601		3R	6,385,173	6,385,786	614	**hth**	+45624	**hth**	+45624	5	5	8	8	3.7		
504	PCE8602		3L	5,182,947	5,184,150	1,204	**CG32423**	+24805	CG4669	-9636	4	12	3	10	3.7		

	CRM	Overlaps known element	Chrom arm	pCRM start	pCRM end	pCRM len	5' gene	pCRM relative position	3' gene	pCRM relative position	Aligned sites	Aligned + preserved sites	Aligned site dens	Aligned + preserved site dens	z-score	Additional Gap/pair-rule gene within 20kb	pCRM relative position
505	PCE8603		2L	15,081,609	15,083,381	1,773	BG:DS04929.1	+27880	BG:DS04929.3	-4288	8	9	5	5	3.7		
506	PCE8604		3L	3,867,701	3,868,315	615	Awh	+40862	BEST:HL04053	-4887	5	5	8	8	3.7		
507	PCE8605		3L	14,748,256	14,748,909	654	CG9425	+7246	NHP2	+2160	4	7	6	11	3.7		
508	PCE8606		3R	6,925,760	6,926,458	699	CG4695	+56049	CG6629	+14988	5	6	7	9	3.7		
509	PCE8607		3L	621,934	622,549	616	CG32345	-18989	CG12030	-6596	5	5	8	8	3.7		
510	PCE8610		3R	23,227,085	23,228,234	1,150	CG12509	-3840	side	+5378	7	7	6	6	3.7		
511	PCE8611		2L	6,219,157	6,220,296	1,140	**Ugt37b1**	+1893	CG9486	-27794	3	13	3	11	3.7		
512	PCE8612		2L	17,285,680	17,286,383	704	CG6380	-15390	CG31804	-55194	5	6	7	9	3.7		
513	PCE8613		3L	4,141,304	4,142,260	957	CG15001	-29	mas	+5827	4	10	4	10	3.7		
514	PCE8615		3L	20,627,921	20,628,582	662	**kni**	-14020	CG13253	+9154	4	7	6	11	3.7		
515	PCE8616		3L	14,248,363	14,249,388	1,026	fz	+25109	fz	+25109	5	9	5	9	3.7		
516	PCE8617		2L	14,190,690	14,191,849	1,160	smi35A	-10665	wb	-53824	5	10	4	9	3.7		
517	PCE8619		2R	15,484,116	15,485,210	1,095	CG11192	-65381	CG33041	-2836	6	8	5	7	3.7		
518	PCE8620		2R	16,617,033	16,617,743	711	CG10440	-2030	CG30222	-9007	5	6	7	8	3.7		
519	PCE8621		2L	13,925,657	13,926,168	512	CG16885	-8814	BG:DS00180.3	+3458	4	5	8	10	3.7		
520	PCE8622		2L	17,513,131	17,513,936	806	CG15145	-3653	CG7094	+19912	5	7	6	9	3.7		
521	PCE8623		2L	14,296,353	14,297,064	712	CG32970	+6045	wb	+51391	5	6	7	8	3.7		
522	PCE8624		3R	13,644,559	13,645,270	712	**CG31246**	+7279	**CG31246**	+7279	5	6	7	8	3.7		
523	PCE8625		3R	24,747,907	24,748,492	586	CG11828	+9700	CG14521	+36950	4	6	7	10	3.7		
524	PCE8626		X	4,811,169	4,812,137	969	**ovo**	+6323	CG32767	+9550	4	10	4	10	3.7		
525	PCE8627		X	20,585,270	20,587,233	1,964	CG11229	+1175	CG11227	+19264	7	11	4	6	3.7		
526	PCE8628		2L	1,567,014	1,567,727	714	CG14351	+10190	CG14351	+10190	5	6	7	8	3.7		
527	PCE8629		3R	17,876,346	17,877,449	1,104	how	+17429	how	+17429	6	8	5	7	3.7		
528	PCE8631		2L	4,062,649	4,063,364	716	ed	+38702	ed	+38702	5	6	7	8	3.7		
529	PCE8632		3L	5,813,553	5,814,224	672	vn	-8631	CG5568	-16585	4	7	6	10	3.7		
530	PCE8633		3R	19,532,259	19,533,374	1,116	nau	+1362	nau	+1362	6	8	5	7	3.7		
531	PCE8634		3L	5,949,714	5,950,761	1,048	**CG10479**	-14401	CG32406	-5760	5	9	5	9	3.6		
532	PCE8635		3R	10,241,244	10,241,879	636	CG14846	-5097	CG14847	+1330	5	5	8	8	3.6		
533	PCE8636		X	13,608,841	13,609,517	677	CG32611	+6654	CG32611	+6654	4	7	6	10	3.6		
534	PCE8637		3L	2,706,795	2,707,471	677	CG32296	+39638	CG32296	+39638	4	7	6	10	3.6		
535	PCE8638		2L	6,079,474	6,080,398	925	Kr-h1	+3233	Kr-h1	+3233	3	11	3	12	3.6		
536	PCE8639		3R	12,328,716	12,329,536	821	decay	-1304	CG11769	-9653	5	7	6	9	3.6		
537	PCE8640		3R	8,901,043	8,901,919	877	**sim**	+2901	**sim**	+2901	6	6	7	7	3.6		
538	PCE8641		X	2,614,829	2,615,350	522	EG:BACR43E12.1	+48091	EG:BACR43E12.7	+14242	4	5	8	10	3.6		
539	PCE8642		3R	14,287,449	14,288,813	1,365	fru	+25920	fru	+25920	7	8	5	6	3.6		
540	PCE8643		X	7,150,784	7,151,780	997	CG32727	+3198	CG9657	-7190	6	7	6	7	3.6		
541	PCE8644		3R	9,293,440	9,294,271	832	CG17025	-1593	CG12538	+55005	5	7	6	8	3.6		
542	PCE8645		3R	6,414,925	6,415,526	602	**hth**	+15884	**hth**	+15884	4	6	7	10	3.6		
543	PCE8647		3R	21,565,329	21,566,272	944	CG4861	+1810	CG4861	+1810	5	8	5	8	3.6		
544	PCE8648		3R	19,685,652	19,687,030	1,379	Pli	+19424	Pli	+19424	5	11	4	8	3.6		
545	PCE8649		3L	10,744,922	10,745,526	605	ninA	-90519	CG12523	-13194	4	6	7	10	3.6		
546	PCE8650		3R	13,897,919	13,898,867	949	**htl**	-19198	CG14317	-7990	5	8	5	8	3.6		
547	PCE8651		2R	13,094,956	13,097,026	2,071	CG10910	+10974	CG5773	+10452	5	14	2	7	3.6		
548	PCE8653		3R	675,079	675,817	739	CG14659	-18095	**opa**	-2748	5	6	7	8	3.6		
549	PCE8655		3R	20,555,177	20,555,870	694	**tok**	+18743	**tok**	+18743	4	7	6	10	3.6	**tld**	-10935
550	PCE8656		X	2,168,910	2,169,699	790	EG:BACH7M4.4	-1239	CG32797	+12778	4	8	5	10	3.6	**gt**	16825
551	PCE8657		3L	7,428,357	7,429,051	695	Tsp66A	+1288	CG8543	+713	4	7	6	10	3.6		
552	PCE8658		2L	2,385,887	2,386,499	613	CG3557	-7528	CG9887	-6373	4	6	7	10	3.6		
553	PCE8659		2L	4,580,752	4,581,992	1,241	CG15636	-10080	CG15635	-53692	5	10	4	8	3.6		
554	PCE8660		3L	13,231,546	13,232,578	1,033	caps	+45311	caps	+45311	6	7	6	7	3.5		
555	PCE8661		2L	17,019,639	17,020,887	1,249	CG6304	-28501	CG15136	+5178	5	10	4	8	3.5		
556	PCE8662		2L	20,255,372	20,256,226	855	CG17570	-21332	CG12617	-8221	5	7	6	8	3.5		
557	PCE8663		2L	15,603,380	15,604,236	857	Or35a	-1738	BG:BACR44L22.6	-2531	5	7	6	8	3.5		
558	PCE8665		3R	3,995,668	3,996,708	1,041	**grn**	+10471	**grn**	+10471	6	7	6	7	3.5		
559	PCE8666		3L	10,205,744	10,206,406	663	Or67c	-3509	can	+18234	5	5	8	8	3.5		
560	PCE8668		2R	14,906,372	14,907,718	1,347	Toll-7	+15755	Obp56i	-24110	6	9	4	7	3.5		
561	PCE8669		3L	7,924,102	7,924,570	469	**exex**	+16382	RNaseX25	-5553	4	4	9	9	3.5		
562	PCE8670		3L	5,977,156	5,978,265	1,110	CG10478	-6897	CG32406	+21744	5	9	5	8	3.5		
563	PCE8671		2L	10,888,242	10,889,219	978	CG14071	-7055	CG14070	+4885	5	8	5	8	3.5		
564	PCE8672		3R	18,010,835	18,011,500	666	CG31163	+60292	CG31163	+60292	5	5	8	8	3.5		
565	PCE8673		X	19,537,088	19,537,895	808	CG12701	-23200	skpD	-5793	4	8	5	10	3.5		
566	PCE8674		2L	706,502	707,044	543	**ds**	+9589	**ds**	+9589	4	5	7	9	3.5		
567	PCE8675		3L	2,971,680	2,973,339	1,660	CG2107	+10021	CG2113	-2469	7	9	4	5	3.5		

	CRM	Overlaps known element	Chrom arm	pCRM start	pCRM end	pCRM len	5' gene	pCRM relative position	3' gene	pCRM relative position	Aligned sites	Aligned + preserved sites	Aligned site dens	Aligned + preserved site dens	z-score	Additional Gap/pair-rule gene within 20kb	pCRM relative position
568	PCE8676		3R	7,169,670	7,170,381	712	CG31386	-48672	KP78b	+3800	4	7	6	10	3.5		
569	PCE8677		3L	4,901,455	4,902,168	714	Rh50	+13129	CG32233	-25223	4	7	6	10	3.5		
570	PCE8678		X	3,804,896	3,806,094	1,199	CG6414	-92932	CG32790	-12437	6	8	5	7	3.5		
571	PCE8679	slp1 early	2L	3,816,761	3,817,475	715	CG3407	-10785	**slp1**	-773	4	7	6	10	3.5	**slp2**	-12649
572	PCE8680		3L	12,536,054	12,536,768	715	CG10632	-5985	**ara**	-1452	4	7	6	10	3.5		
573	PCE8682		X	8,498,395	8,499,112	718	Lim1	+45662	Lim1	+45662	4	7	6	10	3.5		
574	PCE8683		3R	21,742,370	21,743,501	1,132	CG6111	-1341	CG14547	+13211	5	9	4	8	3.5		
575	PCE8684		3L	7,422,400	7,424,500	2,101	CG8546	+6843	Tsp66A	-1498	8	9	4	4	3.5		
576	PCE8685		2L	17,208,032	17,208,749	718	beat-IIIc	+30066	beat-IIIc	+30066	2	10	3	14	3.5		
577	PCE8686		2L	16,901,929	16,902,560	632	CG4841	+13168	beat-IIIb	-60178	4	6	6	9	3.5		
578	PCE8688		2L	14,661,352	14,662,733	1,382	osp	+8733	osp	+8733	4	12	3	9	3.5		
579	PCE8689		2L	9,367,850	9,369,333	1,484	CG13111	-2024	CG4450	+7986	5	11	3	7	3.5		
580	PCE8690		2R	11,099,400	11,100,076	677	SP2353	+3716	SP2353	+3716	3	8	4	12	3.5		
581	PCE8691		X	8,532,373	8,533,595	1,223	Lim1	+11179	Lim1	+11179	4	11	3	9	3.4		
582	PCE8692		2R	10,858,897	10,859,846	950	CG30321	+1849	CG30084	+18792	6	6	6	6	3.4		
583	PCE8693		3R	19,312,384	19,313,067	684	CG17382	+8271	Irk2	+21625	5	5	7	7	3.4		
584	PCE8694		3R	19,436,740	19,437,891	1,152	CG31145	+48144	CG31145	+48144	5	9	4	8	3.4		
585	PCE8695		3R	24,749,544	24,750,228	685	CG11828	+11337	CG14521	+35214	5	5	7	7	3.4		
586	PCE8696		2L	15,176,099	15,176,990	892	BG:DS03192.2	-19694	BG:DS07295.1	+47013	5	7	6	8	3.4		
587	PCE8697		2L	4,101,077	4,102,158	1,082	ed	+77130	ed	+77130	4	10	4	9	3.4		
588	PCE8698		2L	11,476,016	11,477,334	1,319	**sala**	+957	CG6488	+10656	5	10	4	8	3.4		
589	PCE8699		2R	14,074,331	14,075,114	784	CG15105	+19577	5-HT1B	+17583	5	6	6	8	3.4		
590	PCE8700		2L	3,629,528	3,630,422	895	**for**	+2067	**for**	+2067	5	7	6	8	3.4		
591	PCE8701		X	7,383,732	7,384,465	734	ct	+11130	ct	+11130	4	7	5	10	3.4		
592	PCE8702		3R	3,274,891	3,275,786	896	CG14598	+9385	alpha-Est10	+39026	5	7	6	8	3.4		
593	PCE8703		2L	1,599,447	1,600,773	1,327	CG31935	+12018	CG31935	+12018	5	10	4	8	3.4		
594	PCE8705		X	20,203,680	20,204,324	645	Tak1	-5956	CG32504	+1493	4	6	6	9	3.4		
595	PCE8706		2L	5,426,216	5,426,907	692	H15	+29660	CG31647	+4585	5	5	7	7	3.4		
596	PCE8707		3L	11,825,298	11,825,991	694	CG14129	-4588	CG5906	+2862	5	5	7	7	3.4		
597	PCE8708		2R	7,330,030	7,330,677	648	Cam	+758	Cam	+758	4	6	6	9	3.4		
598	PCE8709		3R	15,895,707	15,896,448	742	CG4390	-10203	Rh3	+11741	4	7	5	9	3.4		
599	PCE8710		3R	15,807,348	15,808,255	908	CG31213	+8097	Hs6st	+26006	5	7	6	8	3.4		
600	PCE8711		3L	2,796,072	2,797,251	1,180	CG2083	-15313	CG14952	+31273	5	9	4	8	3.4		
601	PCE8712		2L	8,740,307	8,741,050	744	raw	-7443	CG12438	-3580	4	7	5	9	3.4		
602	PCE8713		3L	16,350,279	16,351,939	1,661	CG13035	-1343	fax	+5385	6	10	4	6	3.4		
603	PCE8714		2R	6,137,844	6,139,028	1,185	CG17326	-81535	CG13235	-6749	5	9	4	8	3.4		
604	PCE8715		3R	10,251,806	10,252,457	652	CG14843	+5508	CG31330	+6463	4	6	6	9	3.4		
605	PCE8716		3R	6,020,697	6,021,349	653	Syn	+1095	Syn	+1095	4	6	6	9	3.4		
606	PCE8717		2L	19,575,634	19,576,288	655	CG10366	+13293	Lar	-13204	4	6	6	9	3.4		
607	PCE8718		X	7,210,892	7,211,594	703	CG11368	+18308	CG32719	+41626	5	5	7	7	3.4		
608	PCE8719		X	12,023,854	12,025,667	1,814	Ten-a	+63167	Ten-a	+63167	7	9	4	5	3.4		
609	PCE8720		2R	17,064,846	17,065,896	1,051	CG13499	+17086	CG13499	+17086	5	8	5	8	3.4		
610	PCE8721		2L	18,345,794	18,346,655	862	Acp36DE	-8685	Fas3	+48472	4	8	5	9	3.4		
611	PCE8722		3L	13,573,722	13,574,380	659	bru-3	+49937	bru-3	+49937	4	6	6	9	3.4		
612	PCE8723		2L	18,597,305	18,598,229	925	**MESR3**	+1966	**MESR3**	+1966	5	7	5	8	3.4		
613	PCE8724		2R	3,332,619	3,333,375	757	CG30371	-2564	CG30358	-47218	4	7	5	9	3.3		
614	PCE8725		3L	10,486,389	10,487,146	758	CG32062	+12856	CG32062	+12856	4	7	5	9	3.3		
615	PCE8726		3R	17,753,035	17,754,421	1,387	CG31171	-3529	Eip93F	-37890	5	10	4	7	3.3		
616	PCE8727		3R	9,152,882	9,153,545	664	**CG32473**	+17756	CG8795	+6359	4	6	6	9	3.3		
617	PCE8728		2R	18,216,678	18,218,161	1,484	CG30188	+96	CG13548	+2072	4	12	3	8	3.3		
618	PCE8729		3R	2,401,337	2,402,049	713	CG33202	+17716	CG2336	-4527	5	5	7	7	3.3		
619	PCE8730		2L	4,149,970	4,151,033	1,064	CG2955	+8123	Or24a	+7637	5	8	5	8	3.3		
620	PCE8731		X	13,677,033	13,678,097	1,065	CG32606	-31270	CG11068	-7812	5	8	5	8	3.3		
621	PCE8732		3R	26,643,616	26,644,432	817	CG15544	+679	CG15544	+679	5	6	6	7	3.3		
622	PCE8733		2L	13,562,988	13,563,860	873	kuz	+30697	kuz	+30697	4	8	5	9	3.3		
623	PCE8734		3R	9,766,472	9,767,187	716	**ems**	+38873	CG9929	+1956	5	5	7	7	3.3		
624	PCE8735		2L	7,528,873	7,529,536	664	CG13789	-54	CG13790	+31513	2	9	3	14	3.3		
625	PCE8736		3R	18,613,312	18,614,025	714	CG7029	+12451	CG7031	-6643	3	8	4	11	3.3		
626	PCE8737		3R	15,770,642	15,771,644	1,003	**mira**	-9783	CG4783	-2564	4	9	4	9	3.3		
627	PCE8738		X	17,841,850	17,842,791	942	wupA	+2896	wupA	+2896	5	7	5	7	3.3		
628	PCE8739		X	19,501,097	19,502,252	1,156	CG12702	-7814	CG12701	+11304	6	7	5	6	3.3		
629	PCE8741		3R	24,756,547	24,757,269	723	CG14521	+28173	CG14521	+28173	5	5	7	7	3.3		
630	PCE8743		3L	20,859,949	20,860,629	681	fng	+6156	fng	+6156	4	6	6	9	3.3		

	CRM	Overlaps known element	Chrom arm	pCRM start	pCRM end	pCRM len	5' gene	pCRM relative position	3' gene	pCRM relative position	Aligned sites	Aligned + preserved sites	Aligned site dens	Aligned + preserved site dens	z-score	Additional Gap/pair-rule gene within 20kb	pCRM relative position
631	PCE8744		3L	7,254,218	7,254,999	782	unc-13-4A	-9702	CG8607	-24599	4	7	5	9	3.3		
632	PCE8745		3L	15,974,355	15,974,946	592	CG5841	-13664	Notum	-3320	4	5	7	8	3.3		
633	PCE8746		3R	7,878,145	7,878,985	841	CG14739	-3192	CG14740	+9700	5	6	6	7	3.3		
634	PCE8747		3L	18,389,353	18,390,136	784	skl	-537	CG32196	+19059	4	7	5	9	3.3		
635	PCE8748		3L	7,155,396	7,156,129	734	CG32387	-11965	CG14826	-38183	5	5	7	7	3.3		
636	PCE8749		3R	12,547,814	12,548,778	965	**Ubx**	+11588	**Ubx**	+11588	5	7	5	7	3.3		
637	PCE8750		2L	21,722,836	21,723,621	786	**tsh**	+61121	CG11629	+7761	4	7	5	9	3.3		
638	PCE8751		X	8,582,666	8,584,615	1,950	CG32710	+24446	CG12075	-18496	5	12	3	6	3.2		
639	PCE8752		2L	5,340,183	5,340,779	597	nompC	+1376	nompC	+1376	4	5	7	8	3.2		
640	PCE8753		2R	15,156,019	15,156,707	689	CG16898	-87320	**18w**	-18515	4	6	6	9	3.2		
641	PCE8755		3L	18,378,400	18,379,089	690	rpr	-31118	skl	+9727	4	6	6	9	3.2		
642	PCE8756		X	7,114,744	7,115,343	600	CG1677	+31393	CG15033	-3296	4	5	7	8	3.2		
643	PCE8757		2R	9,860,958	9,861,476	519	**kn**	+1722	CG12856	-528	4	4	8	8	3.2		
644	PCE8759		3L	18,732,240	18,733,155	916	ftz-f1	+14455	ftz-f1	+14455	4	8	4	9	3.2		
645	PCE8760		X	12,098,612	12,099,309	698	Ten-a	+137925	CG2577	-4561	4	6	6	9	3.2		
646	PCE8761		3L	561,185	561,886	702	CG17090	+34486	CG12169	-79	4	6	6	9	3.2		
647	PCE8763		2L	15,921,132	15,921,743	612	BG:DS07486.4	+1489	BG:DS07486.4	+1489	4	5	7	8	3.2		
648	PCE8764		X	4,534,933	4,535,934	1,002	CG6978	+10123	CG2861	+8380	5	7	5	7	3.2		
649	PCE8765		2L	12,649,899	12,650,970	1,072	**pdm2**	+3201	**pdm2**	+3201	4	9	4	8	3.2		
650	PCE8767		3L	3,569,765	3,571,304	1,540	Eip63E	+43815	Eip63E	+43815	7	7	5	5	3.2		
651	PCE8768		3L	14,054,118	14,055,058	941	Sox21a	+896	Sox21a	+896	4	8	4	9	3.2		
652	PCE8769		3R	21,313,461	21,314,032	572	CG5112	+6885	CG5107	-6267	3	6	5	10	3.2		
653	PCE8771		2L	3,678,899	3,679,911	1,013	CG2808	+2630	CG10017	-2978	5	7	5	7	3.2		
654	PCE8772		2L	14,180,071	14,181,083	1,013	smi35A	-46	wb	-64590	5	7	5	7	3.2		
655	PCE8773		2R	3,849,469	3,850,633	1,165	BcDNA:GH05582	+39723	CG8746	-2746	5	8	4	7	3.1		
656	PCE8774		3R	24,446,054	24,446,822	769	CG10011	+3329	CG10011	+3329	5	5	7	7	3.1		
657	PCE8775		2L	4,273,064	4,273,778	715	CG3675	-1205	tutl	-1941	4	6	6	8	3.1		
658	PCE8776		2L	600,954	601,726	773	**Gsc**	-4602	CG13689	-3050	5	5	6	6	3.1		
659	PCE8777		2L	18,912,810	18,914,163	1,354	CG18397	+7868	CG18397	+7868	5	9	4	7	3.1		
660	PCE8778		2L	12,920,116	12,920,836	721	ACXE	+5628	CG16800	-34863	4	6	6	8	3.1		
661	PCE8779		2L	11,711,164	11,712,191	1,028	CG4988	+27438	CG12602	-15631	5	7	5	7	3.1		
662	PCE8781		3R	8,443,786	8,444,682	897	CG18553	-57091	CG31355	-2672	5	6	6	7	3.1		
663	PCE8782		3R	12,722,692	12,723,803	1,112	CG10349	+17228	CG31270	+25719	6	6	5	5	3.1		
664	PCE8783		3L	13,505,475	13,506,016	542	bru-3	+118301	bru-3	+118301	4	4	7	7	3.1		
665	PCE8784		2R	10,985,716	10,986,555	840	sli	-31137	CG8291	+5500	4	7	5	8	3.1		
666	PCE8786		2R	11,608,905	11,610,022	1,118	CG4750	+10202	Syn2	-5962	6	6	5	5	3.1		
667	PCE8787		3R	16,708,448	16,709,735	1,288	TotZ	+4974	CG10830	+13324	6	7	5	5	3.1		
668	PCE8788		X	11,274,796	11,276,520	1,725	CG32666	+39688	CG32666	+39688	6	9	3	5	3.1		
669	PCE8789		3R	10,412,310	10,413,906	1,597	CG7987	-10722	stumps	-4296	5	10	3	6	3.1	**CG8066**	-17760
670	PCE8791		X	5,889,580	5,890,212	633	Ca-alpha1T	-3816	CG32750	+46581	4	5	6	8	3.1		
671	PCE8792		2R	9,858,755	9,859,662	908	**kn**	+3925	CG12856	-2342	5	6	6	7	3.1		
672	PCE8793		3R	3,422,212	3,423,115	904	CG32465	-15206	CG14597	+6367	3	9	3	10	3.1		
673	PCE8794		3R	4,957,708	4,958,341	634	pum	+24747	pum	+24747	4	5	6	8	3.1		
674	PCE8795		3R	18,844,072	18,844,706	635	lmd	-981	CG13833	+7179	4	5	6	8	3.1		
675	PCE8796		X	18,734,114	18,734,846	733	CG32541	+72798	CG32541	+72798	2	9	3	12	3.1		
676	PCE8797		2L	13,547,490	13,548,287	798	kuz	+15199	kuz	+15199	5	5	6	6	3.1		
677	PCE8798		3R	27,451,745	27,452,886	1,142	CG1800	+18681	CG1804	-21119	4	9	4	8	3.0		
678	PCE8799		2L	12,892,807	12,894,341	1,535	kek1	-81150	ACXC	-7058	6	8	4	5	3.0		
679	PCE8800		3L	18,357,706	18,358,774	1,069	rpr	-10424	skl	+30042	5	7	5	7	3.0		
680	PCE8801		2L	8,327,828	8,328,472	645	CG14275	+6548	CG14274	-3960	4	5	6	8	3.0		
681	PCE8802		2R	15,555,002	15,555,696	695	CG30143	+19750	Obp57c	-11571	3	7	4	10	3.0		
682	PCE8803		3L	2,814,343	2,815,671	1,329	CG2083	-33584	CG14952	+12853	4	10	3	8	3.0		
683	PCE8804		3R	14,816,598	14,819,095	2,498	**gukh**	+6835	**gukh**	+6835	6	11	2	4	3.0		
684	PCE8805		X	7,135,635	7,136,875	1,241	CG1677	+10502	CG15035	+6676	5	8	4	6	3.0		
685	PCE8806	CE8019	3L	9,529,914	9,530,990	1,077	CG32048	+10088	CG32048	+10088	5	7	5	6	3.0		
686	PCE8807		3L	7,787,714	7,788,868	1,155	Pdp1	+1457	Pdp1	+1457	4	9	3	8	3.0		
687	PCE8808		2R	17,039,753	17,040,755	1,003	CG30405	-588	CG13499	+42227	4	8	4	8	3.0		
688	PCE8809		3R	5,304,512	5,305,592	1,081	CG16779	-2991	CG8147	-14948	5	7	5	6	3.0		
689	PCE8810		2R	7,839,090	7,839,843	754	CG12374	+12266	sca	-5414	4	6	5	8	3.0		
690	PCE8811		2L	7,461,446	7,462,525	1,080	CG6055	+2666	CG6055	+2666	3	10	3	9	3.0		
691	PCE8812		3L	12,933,673	12,934,759	1,087	CG10749	+3653	CG11262	-17922	5	7	5	6	3.0		
692	PCE8813		3R	6,104,608	6,105,261	654	CG11870	+11114	CG11870	+11114	4	5	6	8	3.0		
693	PCE8814		X	4,942,026	4,942,901	876	CG15464	+14280	rg	-18943	4	7	5	8	3.0		

	CRM	Overlaps known element	Chrom arm	pCRM start	pCRM end	pCRM len	5' gene	pCRM relative position	3' gene	pCRM relative position	Aligned sites	Aligned + preserved sites	Aligned site dens	Aligned + preserved site dens	z-score	Additional Gap/pair-rule gene within 20kb	pCRM relative position
694	PCE8815		2L	4,129,758	4,130,519	762	Sr-CI	+15469	CG2955	-11328	4	6	5	8	3.0		
695	PCE8816		X	7,308,243	7,309,337	1,095	CG32720	-30977	CG11369	-5637	5	7	5	6	3.0		
696	PCE8817		3R	9,398,335	9,399,433	1,099	CG31337	-27210	CG14370	-14494	5	7	5	6	3.0		
697	PCE8818		2R	16,838,007	16,838,666	660	lox2	-2801	CG18735	+4051	4	5	6	8	3.0		
698	PCE8819		2L	5,226,813	5,228,290	1,478	**tkv**	+1639	**tkv**	+1639	5	9	3	6	3.0		
699	PCE8820		3L	19,382,749	19,383,316	568	CG32206	-20978	CG33062	+11946	4	4	7	7	3.0		
700	PCE8821		3L	14,266,464	14,267,571	1,108	fz	+43210	fz	+43210	5	7	5	6	3.0		
701	PCE8822		X	8,122,301	8,123,407	1,107	nAcRalpha-7E	-53677	CG1387	-1319	3	10	3	9	3.0		
702	PCE8824		3L	14,854,028	14,854,802	775	CG13467	+1036	CG13466	-15777	4	6	5	8	3.0		
703	PCE8825		2R	10,684,893	10,685,730	838	CG30465	+14902	fus	+43063	5	5	6	6	3.0		
704	PCE8826		3L	22,759,170	22,759,946	777	CG11226	+4825	CG32462	+10844	4	6	5	8	3.0		
705	PCE8827		3L	12,828,922	12,830,219	1,298	CG10943	-8657	CG14120	-12379	5	8	4	6	3.0		
706	PCE8828		3R	15,192,208	15,193,724	1,517	**Dl**	-40256	CG3581	+6764	5	9	3	6	2.9		
707	PCE8829		3L	2,199,533	2,200,661	1,129	CG15878	-6401	CG13809	-9613	5	7	4	6	2.9		
708	PCE8830		2L	13,992,126	13,992,703	578	rk	-8722	bgm	-3196	4	4	7	7	2.9		
709	PCE8831		3R	16,251,146	16,252,195	1,050	CG10881	+17143	CG17208	+11468	4	8	4	8	2.9		
710	PCE8832		3L	4,358,243	4,358,919	677	CG18314	+1150	CG18314	+1150	4	5	6	7	2.9		
711	PCE8833		3R	9,406,850	9,407,638	789	CG31337	-35725	CG14370	-6289	4	6	5	8	2.9		
712	PCE8834		3L	6,008,609	6,009,288	680	CG6467	+10145	CG6483	-793	4	5	6	7	2.9		
713	PCE8835		3R	14,089,456	14,090,136	681	CG31241	+8189	CG18599	-3389	4	5	6	7	2.9		
714	PCE8836		2L	18,855,255	18,855,935	681	**tup**	+3404	**tup**	+3404	4	5	6	7	2.9		
715	PCE8837		3R	6,768,536	6,769,119	584	CG6621	-49266	CG4683	-6939	4	4	7	7	2.9		
716	PCE8838		2R	19,345,433	19,346,672	1,240	CG3394	+6348	betaTub60D	-2777	6	6	5	5	2.9		
717	PCE8839		3L	18,396,715	18,397,508	794	skl	-7899	CG32196	+11687	4	6	5	8	2.9		
718	PCE8840		X	3,401,018	3,402,260	1,243	AlstR	+68044	AlstR	+68044	4	9	3	7	2.9		
719	PCE8841		3R	4,219,718	4,220,404	687	CG31264	+3319	CG31264	+3319	4	5	6	7	2.9		
720	PCE8842		3R	24,993,421	24,994,109	689	CG11898	+12541	CG14509	+15973	4	5	6	7	2.9		
721	PCE8843		2L	9,705,516	9,706,106	591	CG4364	-2018	Nckx30C	+32587	4	4	7	7	2.9		
722	PCE8844		3L	22,083,500	22,084,305	806	CG15374	+796	olf413	-16737	4	6	5	7	2.9		
723	PCE8845		3L	18,893,491	18,894,184	694	CG32204	+731	CG32204	+731	4	5	6	7	2.9		
724	PCE8846		3R	12,776,906	12,777,842	937	**Abd-B**	+6900	**Abd-B**	+6900	4	7	4	7	2.9		
725	PCE8847		3L	16,584,358	16,585,622	1,265	Abl	+11831	Abl	+11831	4	9	3	7	2.9		
726	PCE8850		2L	2,013,055	2,013,869	815	CG15361	+2861	CG4238	-11838	4	6	5	7	2.8		
727	PCE8851		2L	17,064,215	17,065,029	815	CG15136	-38150	CG12620	-781	4	6	5	7	2.8		
728	PCE8852		3R	25,333,346	25,334,048	703	CG2014	+2414	**Dr**	-37426	4	5	6	7	2.8		
729	PCE8853		3R	26,747,640	26,748,342	703	**Ptx1**	+9222	CG15549	+3253	4	5	6	7	2.8		
730	PCE8854		3L	12,831,188	12,831,893	706	CG10943	-10923	CG14120	-10705	4	5	6	7	2.8		
731	PCE8855		3R	6,452,909	6,453,618	710	**hth**	+10786	**hth**	+10786	4	5	6	7	2.8		
732	PCE8856		2L	14,876,621	14,877,515	895	Mst35Bb	-14822	CG15277	+10336	5	5	6	6	2.8		
733	PCE8857		X	9,142,970	9,144,624	1,655	CG12654	-19750	CG12650	-6051	5	9	3	5	2.8		
734	PCE8858		2R	3,413,989	3,415,032	1,044	CG14755	+5704	CG11635	-3840	5	6	5	6	2.8		
735	PCE8859		3R	6,450,885	6,451,851	967	**hth**	+10894	**hth**	+10894	4	7	4	7	2.8		
736	PCE8860		X	13,602,891	13,603,605	715	CG32611	+12566	CG32611	+12566	4	5	6	7	2.8		
737	PCE8861		3L	13,613,780	13,614,495	716	bru-3	+9822	bru-3	+9822	4	5	6	7	2.8		
738	PCE8862		3L	14,283,029	14,283,745	717	CG13482	+4713	fz	+60491	4	5	6	7	2.8		
739	PCE8863		X	6,787,027	6,788,250	1,224	**CG14427**	+6044	nullo	-1546	5	7	4	6	2.8		
740	PCE8864		3L	19,392,191	19,392,803	613	CG33062	+2459	CG33062	+2459	4	4	7	7	2.8		
741	PCE8865		3R	23,630,245	23,630,963	719	CG12873	-10734	CG5566	+18243	4	5	6	7	2.8		
742	PCE8866		2L	4,009,211	4,009,929	719	CG10039	+8940	ed	-14018	4	5	6	7	2.8		
743	PCE8867		3L	14,119,875	14,120,593	719	Sox21b	-39671	**D**	+6907	4	5	6	7	2.8		
744	PCE8868		3R	26,453,042	26,454,720	1,679	CG2267	-8926	CG31013	+6201	5	9	3	5	2.8		
745	PCE8870		3L	6,075,327	6,076,048	722	Ets65A	+4805	Ets65A	+4805	4	5	6	7	2.8		
746	PCE8871		X	15,130,520	15,131,250	731	cngl	+230	cngl	+230	4	5	5	7	2.8		
747	PCE8872		2L	5,138,812	5,139,545	734	Msp-300	+3096	Msp-300	+3096	4	5	5	7	2.7		
748	PCE8873		X	3,801,447	3,802,375	929	CG6414	-89483	CG32790	-16156	5	5	5	5	2.7		
749	PCE8875		3R	15,955,901	15,956,639	739	Gr92a	+9103	CG5023	-38535	4	5	5	7	2.7		
750	PCE8876		3L	1,883,948	1,884,812	865	CG1887	-5664	CG13925	-17231	4	6	5	7	2.7		
751	PCE8877		3R	19,231,318	19,232,499	1,182	CG4374	-2332	CG31225	-65846	4	8	3	7	2.7		
752	PCE8878		3R	27,123,447	27,124,728	1,282	CG11340	-3277	CG11339	-41430	5	7	4	5	2.7		
753	PCE8879		X	4,501,964	4,503,260	1,297	CG12683	-9728	CG15472	+7427	5	7	4	5	2.7		
754	PCE8880		X	16,347,988	16,348,737	750	CG9782	+3869	CG9782	+3869	4	5	5	7	2.7		
755	PCE8881		3L	15,147,956	15,148,982	1,027	CG7011	-35453	CG6888	-7740	4	7	4	7	2.7		
756	PCE8882		3R	14,837,689	14,838,329	641	**gukh**	+27926	**gukh**	+27926	4	4	6	6	2.7		

	CRM	Overlaps known element	Chrom arm	pCRM start	pCRM end	pCRM len	5' gene	pCRM relative position	3' gene	pCRM relative position	Aligned sites	Aligned + preserved sites	Aligned site dens	Aligned + preserved site dens	z-score	Additional Gap/pair-rule gene within 20kb	pCRM relative position
757	PCE8883		X	12,038,914	12,039,556	643	Ten-a	+78227	Ten-a	+78227	4	4	6	6	2.7		
758	PCE8884		3R	9,428,725	9,429,369	645	CG14369	-10370	CG9759	+5618	4	4	6	6	2.7		
759	PCE8885		2L	16,198,026	16,198,725	700	BG:DS02795.3	+8484	BG:DS07473.1	-13525	3	6	4	9	2.7		
760	PCE8886		3L	5,957,304	5,958,995	1,692	CG32406	+783	CG32406	+783	4	10	2	6	2.7		
761	PCE8887		2L	17,133,997	17,134,893	897	CG31784	-30038	beat-IIIa	-1751	4	6	4	7	2.7		
762	PCE8888		3L	5,937,286	5,937,942	657	**CG10479**	-1973	CG32406	-18579	4	4	6	6	2.6		
763	PCE8889		3L	10,971,954	10,972,729	776	klu	-5598	Fad2	-8341	4	5	5	6	2.6		
764	PCE8890		X	18,310,580	18,311,489	910	Bx	+13285	CG15040	+32043	4	6	4	7	2.6		
765	PCE8891		X	20,593,919	20,594,525	607	CG11229	+9824	CG11227	+11972	3	5	5	8	2.6		
766	PCE8892		3L	3,949,014	3,949,675	662	CG12605	-3614	scrt	-14869	4	4	6	6	2.6		
767	PCE8893		3R	6,945,867	6,947,122	1,256	CG6629	-4421	Ugt86Dd	+7058	4	8	3	6	2.6		
768	PCE8894		X	2,653,986	2,655,142	1,157	EG:BACR43E12.4	+3980	EG:100G7.6	-21398	3	9	3	8	2.6		
769	PCE8895		2L	1,848,412	1,849,322	911	CG31665	+3749	CG31665	+3749	2	9	2	10	2.6		
770	PCE8896		3R	26,491,392	26,492,056	665	CG15541	+27138	CG1342	-13675	4	4	6	6	2.6		
771	PCE8897		2L	3,732,905	3,734,168	1,264	Shaw	+22150	CG10019	-4092	4	8	3	6	2.6		
772	PCE8898		X	2,719,029	2,719,695	667	**rst**	+10908	**rst**	+10908	4	4	6	6	2.6		
773	PCE8900		2R	20,176,889	20,177,557	669	CG30430	+3888	**Tkr**	+5554	4	4	6	6	2.6		
774	PCE8901		2L	14,469,880	14,470,807	928	BG:DS08340.1	-36912	**noc**	-2206	4	6	4	6	2.6		
775	PCE8902		3R	26,917,836	26,918,508	673	CG15553	-1674	CG11318	+5768	4	4	6	6	2.6		
776	PCE8903		2L	7,324,059	7,324,852	794	**wg**	+22739	Wnt6	-18276	4	5	5	6	2.6		
777	PCE8904		3R	25,226,334	25,227,007	674	Cnx99A	-91602	CG11516	-14782	4	4	6	6	2.6		
778	PCE8905		3L	13,244,630	13,245,918	1,289	caps	+58395	Acp70A	-13284	4	8	3	6	2.6		
779	PCE8906		X	19,669,743	19,670,680	938	Nep3	+11996	CG17003	+27475	4	6	4	6	2.6		
780	PCE8907		X	15,344,721	15,345,398	678	sog	+12833	CG32586	-7508	4	4	6	6	2.6		
781	PCE8908		3R	4,019,218	4,020,017	800	**grn**	-12039	CG7800	-23697	4	5	5	6	2.6		
782	PCE8909		X	8,016,087	8,016,823	737	CG1636	+3692	nAcRalpha-7E	+51801	3	6	4	8	2.6		
783	PCE8910		3L	14,199,837	14,200,945	1,109	CG7906	+14234	fz	-22281	4	7	4	6	2.6		
784	PCE8911		3L	3,946,239	3,947,364	1,126	CG12605	-839	scrt	-17180	4	7	4	6	2.5		
785	PCE8914		3R	5,682,722	5,683,950	1,229	CG12806	+9908	CG12806	+9908	3	9	2	7	2.5	**CG12802**	-18690
786	PCE8915		3R	12,378,599	12,379,564	966	CG14888	+2823	**CG14889**	-2330	4	6	4	6	2.5		
787	PCE8916		2L	3,833,662	3,834,807	1,146	**slp2**	+4252	CG3964	-20442	4	7	3	6	2.5	**slp1**	15414
788	PCE8917		2L	11,324,065	11,324,894	830	CG14925	-49591	CG14926	+7240	4	5	5	6	2.5		
789	PCE8918		2L	7,551,823	7,552,522	700	CG13789	-23004	CG13790	+8527	2	7	3	10	2.5		
790	PCE8919		2R	4,089,350	4,090,057	708	CG8170	+1731	CG8170	+1731	4	4	6	6	2.5		
791	PCE8920		2R	14,915,041	14,915,750	710	Toll-7	+24424	Obp56i	-16078	4	4	6	6	2.5		
792	PCE8921		3L	7,251,704	7,252,543	840	unc-13-4A	-7188	CG8607	-27055	4	5	5	6	2.5		
793	PCE8922		2L	12,376,190	12,377,031	842	bru-2	+39170	bru-2	+39170	4	5	5	6	2.5		
794	PCE8923		2L	13,663,800	13,664,511	712	CG31814	+34951	CG31814	+34951	4	4	6	6	2.5		
795	PCE8924		3L	5,443,034	5,443,749	716	CG4835	+3591	CG10630	+14489	4	4	6	6	2.5		
796	PCE8925		3R	18,927,474	18,928,569	1,096	CG13830	+524	CG13830	+524	5	5	5	5	2.4	**cenB1A**	-7306
797	PCE8926		2R	3,366,090	3,366,950	861	CG30371	-36035	CG30358	-13643	4	5	5	6	2.4		
798	PCE8927		2R	5,555,339	5,556,356	1,018	lola	+38626	lola	+38626	4	6	4	6	2.4		
799	PCE8929		X	15,081,220	15,082,529	1,310	CG6227	+5088	Pp1-13C	+9546	3	9	2	7	2.4		
800	PCE8931		3L	8,207,182	8,208,236	1,055	CG7213	-7995	CG13673	-1233	4	6	4	6	2.4		
801	PCE8932		2L	11,734,391	11,735,449	1,059	CG12602	+6569	CG14931	-42097	4	6	4	6	2.4		
802	PCE8934		3L	21,149,023	21,150,411	1,389	Eip78C	+4829	Eip78C	+4829	5	6	4	4	2.3		
803	PCE8935		3L	13,013,902	13,015,182	1,281	CG11279	+9332	CG14115	+3759	4	7	3	5	2.3		
804	PCE8936		2R	17,504,143	17,505,050	908	CG10972	+33115	CG10384	-4970	4	5	4	6	2.3		
805	PCE8937		3R	2,066,819	2,067,904	1,086	BcDNA:LD21503	+6389	CG1153	-6809	4	6	4	6	2.3		
806	PCE8938		3L	5,930,568	5,931,337	770	**CG10479**	+3976	**CG10479**	+3976	4	4	5	5	2.3		
807	PCE8939		3R	13,252,568	13,253,666	1,099	CG5873	+1060	CG5873	+1060	4	6	4	5	2.3		
808	PCE8941		3R	4,927,969	4,929,073	1,105	pum	+54015	pum	+54015	4	6	4	5	2.3		
809	PCE8942		X	14,855,281	14,856,212	932	Cyp4s3	-7728	CG32589	-556	4	5	4	5	2.3		
810	PCE8943		3R	26,131,482	26,132,420	939	hdc	+38461	hdc	+38461	4	5	4	5	2.3		
811	PCE8944		3R	26,798,410	26,799,352	943	5-HT7	+33525	CG31008	+5742	4	5	4	5	2.3		
812	PCE8945		3L	2,834,299	2,835,914	1,616	CG14952	-5775	CG9973	+16425	4	8	2	5	2.3		
813	PCE8946		2L	14,750,365	14,751,153	789	BG:DS06874.3	+6374	CG4650	+1874	2	7	3	9	2.3		
814	PCE8947		X	11,955,066	11,956,855	1,790	CG12720	+28636	Ten-a	-3832	5	7	3	4	2.2		
815	PCE8948		2L	14,098,226	14,099,579	1,354	CG31769	-5335	CG15292	+9738	4	7	3	5	2.2		
816	PCE8949		2R	7,161,647	7,162,781	1,135	jeb	+20804	jeb	+20804	4	6	4	5	2.2		
817	PCE8950		2L	16,386,675	16,387,635	961	**BG:DS02780.1**	+38914	**BG:DS02780.1**	+38914	4	5	4	5	2.2		
818	PCE8951		2R	18,231,835	18,232,712	878	CG13539	+3860	CG3162	+22395	3	6	3	7	2.2		
819	PCE8952		X	8,134,297	8,135,991	1,695	CG1387	+9571	CG15345	+1216	2	11	1	6	2.2		

	CRM	Overlaps known element	Chrom arm	pCRM start	pCRM end	pCRM len	5' gene	pCRM relative position	3' gene	pCRM relative position	Aligned sites	Aligned + preserved sites	Aligned site dens	Aligned + preserved site dens	z-score	Additional Gap/pair-rule gene within 20kb	pCRM relative position
820	PCE8953		2R	15,520,245	15,521,068	824	CG33041	+32199	CG33041	+32199	4	4	5	5	2.2		
821	PCE8954		2R	14,868,501	14,869,689	1,189	Obp56g	-20768	Obp56h	-9600	4	6	3	5	2.2		
822	PCE8955		2L	1,543,570	1,544,651	1,082	Or22b	+19214	CG14351	-12173	1	10	1	9	2.2		
823	PCE8957		3L	4,581,676	4,582,520	845	Src64B	+21617	Src64B	+21617	4	4	5	5	2.1		
824	PCE8959		2L	7,537,895	7,539,321	1,427	CG13789	-9076	CG13790	+21728	1	11	1	8	2.0		
825	PCE8960		3L	19,616,696	19,617,795	1,100	CG8780	-3076	CG8765	+19521	4	5	4	5	2.0		
826	PCE8961		3L	1,683,801	1,684,917	1,117	CG13931	-1289	CG13932	+5992	4	5	4	4	2.0		
827	PCE8962		2L	4,756,465	4,757,387	923	CG15630	+29224	CG15630	+29224	4	4	4	4	2.0		
828	PCE8963		2R	10,196,306	10,197,443	1,138	**CG11798**	+3666	**CG11798**	+3666	4	5	4	4	2.0		
829	PCE8964		2R	12,685,374	12,687,335	1,962	CG10936	+17485	rhi	+9902	3	9	2	5	1.9		
830	PCE8965		2L	6,991,437	6,992,512	1,076	SP1070	-14193	CG13776	-13214	1	9	1	8	1.9		
831	PCE8966		X	9,200,819	9,201,869	1,051	CG15316	+33303	CG15316	+33303	4	4	4	4	1.8		
832	PCE8967		3R	15,827,785	15,829,246	1,462	Hs6st	+5015	Hs6st	+5015	3	7	2	5	1.8		
833	PCE8968		X	6,862,124	6,862,833	710	fz4	-11456	CG32729	-67271	2	5	3	7	1.8		
834	PCE8969		X	11,939,954	11,941,308	1,355	CG12720	+13524	Ten-a	-19379	4	5	3	4	1.7		
835	PCE8970		X	12,120,594	12,121,734	1,141	CG15732	-12179	CG32651	+17331	4	4	4	4	1.7		
836	PCE8971		3R	18,049,626	18,051,202	1,577	CG31163	+43431	CG31163	+43431	4	5	3	3	1.6		
837	PCE8972		X	8,523,578	8,525,143	1,566	Lim1	+19631	Lim1	+19631	2	8	1	5	1.6		
838	PCE8973		2R	11,097,437	11,098,713	1,277	SP2353	+1753	SP2353	+1753	2	7	2	5	1.6		
839	PCE8974		2L	9,166,153	9,167,435	1,283	tai	+7121	tai	+7121	2	7	2	5	1.5		
840	PCE8975		X	19,716,382	19,717,478	1,097	DD2R	-6529	CG9569	+7448	2	6	2	5	1.5		
841	PCE8976		X	4,772,129	4,773,172	1,044	CG12680	+33782	**ovo**	-15738	1	6	1	6	1.1		
842	PCE8978		3R	4,974,678	4,975,780	1,103	pum	+7308	pum	+7308	1	5	1	5	0.8		
