# Supplementary material for: Computational identification of developmental enhancers: conservation and function of transcription factor binding-site clusters in Drosophila melanogaster and Drosophila pseudoobscura
Source: Genome Biol. 2004 Aug 20;5(9):R61. doi: 10.1186/gb-2004-5-9-r61 (PMC522868; doi:10.1186/gb-2004-5-9-r61)
Supplement: Additional data file 8 — All new pCRMs from genome-wide eCIS-ANALYST located more than 20 kb from annotated transcript [file gb-2004-5-9-r61-s8.pdf]

	CRM	Overlaps known element	Chrom arm	pCRM start	pCRM end	pCRM len	5' gene	pCRM relative position	3' gene	pCRM relative position	Aligned sites	Aligned + preserved sites	Aligned site dens	Aligned + preserved site dens	z-score	Additional Gap/pair-rule gene within 20kb	pCRM relative position
1	PCE8058		2L	15,190,185	15,192,070	1,886	BG:DS03192.2	-33780	BG:DS07295.1	+31933	17	20	9	11	9.1		
2	PCE8062		3R	17,769,675	17,771,377	1,703	CG31171	-20169	Eip93F	-20934	13	23	8	14	8.8		
3	PCE8079		3L	19,144,705	19,145,326	622	**fz2**	-25200	mRpL21	+58610	9	9	14	14	7.5		
4	PCE8082	CE8026	3R	11,423,413	11,426,281	2,869	CG18516	-44901	CG5302	-32341	15	20	5	7	7.3		
5	PCE8090		X	4,230,105	4,230,679	575	bi	+76634	CG12685	-25550	7	10	12	17	7.1		
6	PCE8098		X	1,293,402	1,294,046	645	EG:196F3.2	+22907	EG:56G7.1	+20898	8	9	12	14	6.8		
7	PCE8101		3L	19,148,678	19,149,172	495	**fz2**	-29173	mRpL21	+54764	7	7	14	14	6.7		
8	PCE8103		3L	13,694,645	13,696,718	2,074	bru-3	-70328	CG8757	+94402	11	19	5	9	6.6		
9	PCE8117		X	3,759,878	3,761,211	1,334	CG6414	-47914	CG32790	-57320	11	13	8	10	6.5		
10	PCE8129		2R	14,972,099	14,973,036	938	CG13872	-27466	CG30447	-37992	9	11	10	12	6.3		
11	PCE8136		3R	22,574,127	22,575,106	980	CG14247	+21199	Tl	-39023	9	11	9	11	6.2		
12	PCE8143		2L	4,608,553	4,609,408	856	CG15636	-37881	CG15635	-26276	9	9	11	11	6.1		
13	PCE8157		3L	6,803,270	6,805,319	2,050	vvl	+53582	Prat2	+68174	10	17	5	8	5.9		
14	PCE8181		X	7,223,353	7,224,268	916	CG11368	+30769	CG32719	+28952	8	10	9	11	5.7		
15	PCE8211		3L	17,081,997	17,082,675	679	CG32169	-54916	CG32175	-62914	6	9	9	13	5.4		
16	PCE8213		2L	15,126,955	15,127,670	716	stc	+35389	BG:DS03192.2	+28735	7	8	10	11	5.4		
17	PCE8227		X	9,459,015	9,459,728	714	**btd**	+25844	Sp1	-32214	6	9	8	13	5.2		
18	PCE8236		3L	14,425,844	14,427,189	1,346	**CG9598**	-59632	CG9587	+34939	7	14	5	10	5.2		
19	PCE8238		3R	6,816,094	6,817,260	1,167	CG4683	+40036	CG14698	-42974	6	14	5	12	5.2		
20	PCE8241		3L	13,718,324	13,719,301	978	bru-3	-94007	CG8757	+71819	8	9	8	9	5.1		
21	PCE8250		2L	15,504,649	15,505,259	611	lace	+27456	BG:DS04862.2	-23296	6	7	10	11	5.1		
22	PCE8256		3R	23,981,950	23,982,629	680	CG12425	+20039	CG4787	-67557	6	8	9	12	5.1		
23	PCE8262		3L	1,983,617	1,984,483	867	CG1139	+31327	CG32308	+34340	5	12	6	14	5.1		
24	PCE8265		3L	17,067,775	17,068,886	1,112	CG32169	-40694	CG32175	-76703	8	10	7	9	5.0		
25	PCE8266		2L	7,934,371	7,935,089	719	CG14532	-42105	CG7233	+33332	5	10	7	14	5.0		
26	PCE8288		3L	6,806,987	6,808,084	1,098	vvl	+57299	Prat2	+65409	7	11	6	10	4.9		
27	PCE8289		3L	9,880,833	9,881,539	707	CG8104	+24076	CG14163	-20383	6	8	8	11	4.9		
28	PCE8291		3R	7,146,552	7,147,455	904	CG31386	-25554	KP78b	+26726	5	12	6	13	4.9		
29	PCE8299		X	11,021,178	11,023,802	2,625	CG15198	+30831	CG12624	+27945	9	16	3	6	4.9		
30	PCE8303		X	8,100,707	8,101,292	586	nAcRalpha-7E	-32083	CG1387	-23434	6	6	10	10	4.9		
31	PCE8313		X	2,779,499	2,781,121	1,623	**rst**	-48896	CG4116	+43212	6	16	4	10	4.8		
32	PCE8316		3L	18,303,559	18,304,889	1,331	**grim**	-50266	**rpr**	+42393	8	11	6	8	4.8		
33	PCE8366		3L	15,357,679	15,358,733	1,055	**Toll-6**	+72101	CG7804	+64256	6	11	6	10	4.6		
34	PCE8372		2R	15,108,654	15,109,286	633	CG16898	-39955	**18w**	-65936	6	6	9	9	4.6		
35	PCE8374		3L	10,559,477	10,560,308	832	CG32062	+85944	CG14151	+25705	7	7	8	8	4.6		
36	PCE8375		3L	3,662,629	3,663,652	1,024	CG10862	-27229	CG32264	+63648	7	9	7	9	4.6		
37	PCE8384		3L	15,387,239	15,389,216	1,978	**Toll-6**	+101661	CG7804	+33773	7	15	4	8	4.5		
38	PCE8387		3L	17,094,856	17,095,574	719	CG32169	-67775	CG32175	-50015	6	7	8	10	4.5		
39	PCE8400		3L	17,047,361	17,048,418	1,058	CG32169	-20280	CG32175	-97171	7	9	7	9	4.5		
40	PCE8414		2L	16,923,989	16,924,656	668	CG4841	+35228	beat-IIIb	-38082	6	6	9	9	4.4		
41	PCE8457		2R	12,994,419	12,995,795	1,377	elk	+56748	PpY-55A	+24500	8	9	6	7	4.2		
42	PCE8482		2L	15,201,110	15,202,188	1,079	BG:DS03192.2	-44705	BG:DS07295.1	+21815	5	11	5	10	4.1		
43	PCE8496		2L	11,379,043	11,379,593	551	salr	+24857	**salm**	+54881	5	5	9	9	4.1		
44	PCE8506		3L	22,055,340	22,056,006	667	msopa	+55255	CG15374	-26698	4	8	6	12	4.0		
45	PCE8508		3L	18,317,741	18,318,637	897	**grim**	-64448	**rpr**	+28645	5	9	6	10	4.0		
46	PCE8526		X	17,242,931	17,243,574	644	CG8557	-22657	CG12432	+22003	5	6	8	9	4.0		
47	PCE8551		2L	19,194,846	19,195,508	663	drl	+26418	CG31797	+44708	5	6	8	9	3.9		
48	PCE8562		2L	11,385,288	11,386,373	1,086	salr	+31102	**salm**	+48101	7	7	6	6	3.9		
49	PCE8571		3R	15,106,844	15,107,813	970	CG14280	+23840	**Dl**	+44139	5	9	5	9	3.8		
50	PCE8608		3L	22,035,396	22,036,187	792	msopa	+35311	CG15374	-46517	5	7	6	9	3.7		
51	PCE8609		2L	9,039,358	9,040,057	700	CG9582	-21723	CG31708	+23336	5	6	7	9	3.7		
52	PCE8614		X	7,214,348	7,215,053	706	CG11368	+21764	CG32719	+38167	5	6	7	8	3.7		
53	PCE8618		2R	1,613,226	1,614,627	1,402	1.28	+49557	CG15233	+47253	6	10	4	7	3.7		
54	PCE8630		3R	888,267	888,982	716	CG2022	-73796	**corto**	+23423	5	6	7	8	3.7		
55	PCE8646		3R	19,252,743	19,253,575	833	CG4374	-23757	CG31225	-44770	5	7	6	8	3.6		
56	PCE8652		3L	18,314,668	18,316,059	1,392	**grim**	-61375	**rpr**	+31223	7	8	5	6	3.6		
57	PCE8654		2L	17,827,486	17,829,189	1,704	CadN2	-36799	CG5674	-112231	6	11	4	6	3.6		
58	PCE8664		2L	12,556,729	12,557,586	858	**bun**	-27288	CG15489	-25071	5	7	6	8	3.5		
59	PCE8667		2L	7,930,722	7,931,898	1,177	CG14532	-38456	CG7233	+36523	4	11	3	9	3.5		
60	PCE8681		3L	10,558,497	10,559,123	627	CG32062	+84964	CG14151	+26890	4	6	6	10	3.5		
61	PCE8687		X	15,920,993	15,921,765	773	disco-r	-66780	**disco**	+25988	5	6	6	8	3.5		
62	PCE8704		3L	10,328,230	10,328,919	690	CG6559	-57988	CG12362	-41425	5	5	7	7	3.4		
63	PCE8740		X	17,090,028	17,091,547	1,520	B-H2	+44489	B-H1	-36912	6	9	4	6	3.3		

	CRM	Overlaps known element	Chrom arm	pCRM start	pCRM end	pCRM len	5' gene	pCRM relative position	3' gene	pCRM relative position	Aligned sites	Aligned + preserved sites	Aligned site dens	Aligned + preserved site dens	z-score	Additional Gap/pair-rule gene within 20kb	pCRM relative position
64	PCE8742		X	6,903,757	6,904,845	1,089	fz4	-53089	CG32729	-25259	5	8	5	7	3.3		
65	PCE8754		3L	5,363,522	5,364,210	689	CG10633	-21393	CG4814	-28104	4	6	6	9	3.2		
66	PCE8758		3R	15,179,152	15,179,845	694	**Dl**	-27200	CG3581	+20643	4	6	6	9	3.2		
67	PCE8762		3R	10,810,188	10,810,946	759	CG3837	+65769	CG14861	-29563	5	5	7	7	3.2		
68	PCE8766		2R	6,621,834	6,622,541	708	**en**	-28911	tou	+24807	4	6	6	8	3.2		
69	PCE8770		2R	15,450,910	15,451,441	532	CG11192	-32175	CG33041	-36605	4	4	8	8	3.2		
70	PCE8780		3L	12,609,305	12,609,843	539	CG32111	+23823	mirr	-41451	4	4	7	7	3.1		
71	PCE8785		3R	5,781,580	5,782,775	1,196	CG32469	+26927	CG12419	-24745	5	8	4	7	3.1		
72	PCE8790		3R	25,158,018	25,158,650	633	Cnx99A	-23286	CG11516	-83139	4	5	6	8	3.1		
73	PCE8823		2R	15,144,708	15,145,905	1,198	CG16898	-76009	**18w**	-29317	4	9	3	8	3.0		
74	PCE8848		3L	10,297,528	10,298,224	697	CG6559	-27286	CG12362	-72120	4	5	6	7	2.9		
75	PCE8849		X	7,231,805	7,232,750	946	CG11368	+39221	CG32719	+20470	4	7	4	7	2.8		
76	PCE8869		3R	25,219,783	25,220,397	615	Cnx99A	-85051	CG11516	-21392	4	4	7	7	2.8		
77	PCE8874		3R	6,811,167	6,812,024	858	CG4683	+35109	CG14698	-48210	4	6	5	7	2.7		
78	PCE8899		X	13,750,875	13,751,955	1,081	Ste12DOR	+20007	CG32605	+40100	4	7	4	6	2.6		
79	PCE8912		3L	6,364,086	6,364,835	750	CG32398	-34896	CG14910	-56359	3	6	4	8	2.5		
80	PCE8913		3R	13,011,195	13,012,329	1,135	CG4090	-25510	CG31262	+32840	4	7	4	6	2.5		
81	PCE8928		2L	4,591,626	4,592,645	1,020	CG15636	-20954	CG15635	-43039	4	6	4	6	2.4		
82	PCE8930		X	6,154,146	6,155,480	1,335	PpV	-40929	CG3367	+53724	5	6	4	4	2.4		
83	PCE8933		3L	19,169,035	19,170,095	1,061	**fz2**	-49530	mRpL21	+33841	4	6	4	6	2.4		
84	PCE8940		2R	10,133,926	10,135,029	1,104	hbs	+58803	**CG11798**	-57611	4	6	4	5	2.3		
85	PCE8956		3L	3,670,899	3,671,897	999	CG10862	-35499	CG32264	+55403	4	5	4	5	2.2		
86	PCE8958		3R	10,765,993	10,766,966	974	CG3837	+21574	CG14861	-73543	3	6	3	6	2.1		
87	PCE8977	CE8008	X	9,457,113	9,458,627	1,515	**btd**	+23942	Sp1	-33315	2	5	1	3	0.9		
