# Supplementary material for: Computational identification of developmental enhancers: conservation and function of transcription factor binding-site clusters in Drosophila melanogaster and Drosophila pseudoobscura
Source: Genome Biol. 2004 Aug 20;5(9):R61. doi: 10.1186/gb-2004-5-9-r61 (PMC522868; doi:10.1186/gb-2004-5-9-r61)
Supplement: Additional data file 9 — Genes with anterior-posterior patterns and the source of the information [file gb-2004-5-9-r61-s9.pdf]

Gene	Evidence
Antp	Both
argos	Both
bcd	Both
bowl	Both
cad	Both
cas	Both
cnc	Both
comm	Both
D	Both
disco	Both
dpn	Both
emc	Both
en	Both
fkh	Both
gt	Both
hb	Both
htl	Both
ken	Both
knrl	Both
Kr	Both
l(1)sc	Both
noc	Both
Nrt	Both
nub	Both
odd	Both
opa	Both
Optix	Both
ovo	Both
pdm2	Both
Pka-C3	Both
pros	Both
Ptx1	Both
run	Both
slp1	Both
slp2	Both
sob	Both
tkv	Both
trx	Both
tsh	Both
wg	Both
18w	FlyBase
anon-84Bd	FlyBase
btd	FlyBase
byn	FlyBase
cic	FlyBase
dap	FlyBase
Dfd	FlyBase
Dl	FlyBase
dnt	FlyBase
ds	FlyBase
Ecol\lacZ	FlyBase
ems	FlyBase

Gene	Evidence
eve	FlyBase
fog	FlyBase
ftz	FlyBase
fz2	FlyBase
grn	FlyBase
gsb	FlyBase
hh	FlyBase
hkb	FlyBase
hth	FlyBase
iab-4	FlyBase
ImpL2	FlyBase
kni	FlyBase
nkd	FlyBase
nos	FlyBase
oc	FlyBase
ph-d	FlyBase
ph-p	FlyBase
pnr	FlyBase
pnt	FlyBase
psq	FlyBase
pum	FlyBase
Rac2	FlyBase
retn	FlyBase
salm	FlyBase
Scer\GAL4	FlyBase
Six4	FlyBase
sna	FlyBase
Sry-δ	FlyBase
stau	FlyBase
Sxl	FlyBase
Syx1A	FlyBase
tll	FlyBase
Tom	FlyBase
trn	FlyBase
tsg	FlyBase
tsl	FlyBase
vnd	FlyBase
Wnt2	FlyBase
Wnt5	FlyBase
5-HT2	Both
26-29kD-proteinase	BDGP insitu
Adam	BDGP insitu
Alhambra	BDGP insitu
Ama	BDGP insitu
Ance	BDGP insitu
apt	BDGP insitu
ara	BDGP insitu
asp	BDGP insitu
BEAF-32	BDGP insitu
Best2	BDGP insitu
BG DS02780.1	BDGP insitu
BG DS05899.3	BDGP insitu

Gene	Evidence
brat	BDGP insitu
Bsg25A	BDGP insitu
Btk29A	BDGP insitu
bun	BDGP insitu
bwa	BDGP insitu
Cad74A	BDGP insitu
CalpA	BDGP insitu
cenB1A	BDGP insitu
cenG1A	BDGP insitu
CG10176	BDGP insitu
CG10283	BDGP insitu
CG10479	BDGP insitu
CG10728	BDGP insitu
CG10924	BDGP insitu
CG10967	BDGP insitu
CG11188	BDGP insitu
CG11208	BDGP insitu
CG11696	BDGP insitu
CG11798	BDGP insitu
CG12581	BDGP insitu
CG12708	BDGP insitu
CG12802	BDGP insitu
CG13290	BDGP insitu
CG13651	BDGP insitu
CG13894	BDGP insitu
CG14427	BDGP insitu
CG14430	BDGP insitu
CG1447	BDGP insitu
CG14657	BDGP insitu
CG14889	BDGP insitu
CG14937	BDGP insitu
CG1561	BDGP insitu
CG1621	BDGP insitu
CG17390	BDGP insitu
CG17724	BDGP insitu
CG17786	BDGP insitu
CG1815	BDGP insitu
CG2791	BDGP insitu
CG30015	BDGP insitu
CG30023	BDGP insitu
CG30115	BDGP insitu
CG3097	BDGP insitu
CG31246	BDGP insitu
CG31305	BDGP insitu
CG31320	BDGP insitu
CG3136	BDGP insitu
CG31431	BDGP insitu
CG31607	BDGP insitu
CG31670	BDGP insitu
CG31721	BDGP insitu
CG31871	BDGP insitu
CG32026	BDGP insitu

Gene	Evidence
CG32306	BDGP insitu
CG32425	BDGP insitu
CG32434	BDGP insitu
CG32473	BDGP insitu
CG3252	BDGP insitu
CG32982	BDGP insitu
CG33099	BDGP insitu
CG33207	BDGP insitu
CG3424	BDGP insitu
CG3509	BDGP insitu
CG3838	BDGP insitu
CG4040	BDGP insitu
CG4221	BDGP insitu
CG4702	BDGP insitu
CG4710	BDGP insitu
CG5249	BDGP insitu
CG5532	BDGP insitu
CG6654	BDGP insitu
CG6736	BDGP insitu
CG6755	BDGP insitu
CG6885	BDGP insitu
CG7047	BDGP insitu
CG7271	BDGP insitu
CG7428	BDGP insitu
CG7663	BDGP insitu
CG7915	BDGP insitu
CG7986	BDGP insitu
CG8001	BDGP insitu
CG8066	BDGP insitu
CG8092	BDGP insitu
CG8486	BDGP insitu
CG8788	BDGP insitu
CG8965	BDGP insitu
CG9005	BDGP insitu
CG9215	BDGP insitu
CG9238	BDGP insitu
CG9514	BDGP insitu
CG9598	BDGP insitu
CG9615	BDGP insitu
CG9924	BDGP insitu
corto	BDGP insitu
CrebA	BDGP insitu
crq	BDGP insitu
CycE	BDGP insitu
Cyp18a1	BDGP insitu
Cyp306a1	BDGP insitu
Cyt-b5	BDGP insitu
dbo	BDGP insitu
desat1	BDGP insitu
Dg	BDGP insitu
dm	BDGP insitu
Doc1	BDGP insitu

Gene	Evidence
Doc2	BDGP insitu
Doc3	BDGP insitu
Dr	BDGP insitu
drm	BDGP insitu
E2f	BDGP insitu
Egfr	BDGP insitu
Eip71CD	BDGP insitu
Esp	BDGP insitu
exex	BDGP insitu
eya	BDGP insitu
fj	BDGP insitu
fok	BDGP insitu
Gasp	BDGP insitu
Glu-RI	BDGP insitu
grim	BDGP insitu
gukh	BDGP insitu
Hel89B	BDGP insitu
HLHm5	BDGP insitu
ImpE2	BDGP insitu
jbug	BDGP insitu
jing	BDGP insitu
jumu	BDGP insitu
kel	BDGP insitu
kn	BDGP insitu
l(3)10615	BDGP insitu
lab	BDGP insitu
mae	BDGP insitu
MESR3	BDGP insitu
mew	BDGP insitu
mfas	BDGP insitu
Mipp1	BDGP insitu
mira	BDGP insitu
Mkp3	BDGP insitu
mRpS34	BDGP insitu
mspo	BDGP insitu
Ndae1	BDGP insitu
NetA	BDGP insitu
NetB	BDGP insitu
Pepck	BDGP insitu
Pfrx	BDGP insitu
phyl	BDGP insitu
ppa	BDGP insitu
prd	BDGP insitu
Pvf3	BDGP insitu
RhoGEF3	BDGP insitu
rib	BDGP insitu
rst	BDGP insitu
ry	BDGP insitu
sala	BDGP insitu
Sec61beta	BDGP insitu
sim	BDGP insitu
so	BDGP insitu

Gene	Evidence
SoxN	BDGP insitu
srp	BDGP insitu
Sry-beta	BDGP insitu
stwl	BDGP insitu
Su(H)	BDGP insitu
Sulf1	BDGP insitu
tap	BDGP insitu
Thiolase	BDGP insitu
tin	BDGP insitu
Tkr	BDGP insitu
tld	BDGP insitu
tok	BDGP insitu
Toll-6	BDGP insitu
toy	BDGP insitu
Traf1	BDGP insitu
Tre1	BDGP insitu
Ugt37b1	BDGP insitu
Wnt8	BDGP insitu
yellow-e3	BDGP insitu
zfh1	BDGP insitu
