# Supplementary material for: Computational identification of developmental enhancers: conservation and function of transcription factor binding-site clusters in Drosophila melanogaster and Drosophila pseudoobscura
Source: Genome Biol. 2004 Aug 20;5(9):R61. doi: 10.1186/gb-2004-5-9-r61 (PMC522868; doi:10.1186/gb-2004-5-9-r61)
Supplement: Additional data file 10 — All new pCRMs from genome-wide eCIS-ANALYST located within 20 kb of gene with anterior-posterior pattern [file gb-2004-5-9-r61-s10.pdf]

	CRM	Overlaps known element	Chrom arm	pCRM start	pCRM end	pCRM len	5' gene	pCRM relative position	3' gene	pCRM relative position	Aligned sites	Aligned + preserved sites	Aligned site dens	Aligned + preserved site dens	z-score	Additional Gap/pair-rule gene within 20kb	pCRM relative position
1	PCE8050	hairy stripes 3/4,6,7	3L	8,622,879	8,626,839	3,961	CG6486	+14646	**h**	-7829	36	62	9	16	20.1		
2	PCE8051	kni upstream	3L	20,614,714	20,617,020	2,307	**kni**	-813	CG13253	+20716	25	31	11	13	13.2		
3	PCE8052	pdm1 blastoderm	2L	12,604,311	12,606,913	2,603	CG15488	+1653	**nub**	-304	20	33	8	13	11.6		
4	PCE8053	eve stripes 3/7	2R	5,035,493	5,037,290	1,798	CG12134	+3712	**eve**	-2433	21	24	12	13	11.5	**Adam**	+5901
5	PCE8054	hairy stripes 1,5	3L	8,628,846	8,631,011	2,166	CG6486	+20613	**h**	-3657	17	29	8	13	10.5		
6	PCE8055	runt stripe 3	X	20,356,848	20,360,054	3,207	CG1338	-9192	**run**	-6801	17	34	5	11	10.3		
7	PCE8057	hb HZ1.4	3R	4,526,225	4,527,991	1,767	**hb**	-2670	CG8112	+1273	17	21	10	12	9.5		
8	PCE8059	eve stripes 4/6	2R	5,044,597	5,046,030	1,434	**eve**	+4874	TER94	-3763	15	18	10	13	9.0	**Adam**	+15005
9	PCE8060	gt posterior domain	X	2,186,709	2,189,069	2,361	**gt**	-974	tko	+11679	18	21	8	9	8.9		
10	PCE8063	CE8021	3L	18,339,914	18,341,941	2,028	**grim**	-86621	**rpr**	+5341	16	20	8	10	8.5		
11	PCE8065		3R	4,026,032	4,027,816	1,785	**grn**	-18853	CG7800	-15898	15	19	8	11	8.4		
12	PCE8066		X	20,348,460	20,352,624	4,165	CG1338	-804	**run**	-14231	16	28	4	7	8.3		
13	PCE8067	ftz upstream (partial)	3R	2,682,314	2,684,591	2,278	**Scr**	-7972	**ftz**	-5455	15	22	7	10	8.3		
14	PCE8071	sqz neurogenic	3R	14,999,463	15,001,552	2,090	sqz	+9504	CG14282	-1186	12	24	6	11	8.0	**nos**	+16485
15	PCE8076	eve stripe 2	2R	5,038,454	5,039,041	588	CG12134	+6673	**eve**	-682	8	10	14	17	7.6	**Adam**	+8862
16	PCE8080	odd stripes 3/6	2L	3,601,045	3,602,748	1,704	**odd**	-1728	Dot	-9112	12	19	7	11	7.5		
17	PCE8083		3L	14,121,556	14,123,127	1,572	Sox21b	-41352	**D**	+4373	12	17	8	11	7.3		
18	PCE8086		3L	20,612,647	20,614,073	1,427	**kni**	+1254	CG13253	+23663	11	17	8	12	7.2		
19	PCE8088		3L	16,418,107	16,418,469	363	CG33158	+49435	**argos**	+14111	6	6	17	17	7.2		
20	PCE8089		3R	12,368,159	12,368,687	529	CG11769	+28970	CG31448	-670	7	9	13	17	7.2	**CG14889**	-13735
21	PCE8093		3L	15,688,222	15,691,204	2,983	**comm**	-10920	CG13445	-67172	13	22	4	7	7.0		
22	PCE8100	eve early APR	2R	5,042,174	5,042,884	711	**eve**	+2451	TER94	-6909	8	10	11	14	6.7	**Adam**	+12582
23	PCE8102	tll posterior	3R	26,663,942	26,665,204	1,263	CG15544	+21005	**tll**	-2251	11	13	9	10	6.6		
24	PCE8104	ems neurogenic	3R	9,723,602	9,724,936	1,335	E5	-23682	**ems**	-2663	12	12	9	9	6.6		
25	PCE8109		3R	7,941,601	7,942,426	826	CG31361	+17775	**CG4702**	+11512	9	10	11	12	6.5		
26	PCE8110		2L	8,804,166	8,805,336	1,171	CG9468	-30684	**SoxN**	-12519	10	13	9	11	6.5		
27	PCE8115	eve stripe 1	2R	5,046,559	5,047,297	739	**eve**	+6836	TER94	-2496	8	10	11	14	6.5	**Adam**	+16967
28	PCE8118		3R	14,822,848	14,823,484	637	**gukh**	+13085	**gukh**	+13085	8	8	13	13	6.4		
29	PCE8119		3R	12,671,525	12,672,987	1,463	**abd-A**	-15737	CG10349	-32477	11	14	8	10	6.4		
30	PCE8130		3R	12,383,752	12,385,269	1,518	**CG14889**	+1858	**CG14889**	+1858	11	14	7	9	6.3		
31	PCE8137		3R	12,053,627	12,055,472	1,846	**tara**	+2239	**tara**	+2239	10	17	5	9	6.1		
32	PCE8139		2R	6,573,169	6,574,383	1,215	inv	+32752	CG30034	+12378	10	12	8	10	6.1	**en**	+19407
33	PCE8140		2R	15,167,055	15,168,270	1,216	CG16898	-98356	**18w**	-6952	10	12	8	10	6.1		
34	PCE8144		3L	3,503,831	3,504,156	326	Eip63E	+7518	Eip63E	+7518	4	6	12	18	6.1	**ImpE2**	-10525
35	PCE8145		3R	4,536,237	4,536,936	700	CG8112	+1795	CG8112	+1795	8	8	11	11	6.0	**hb**	-12682
36	PCE8150		3R	6,379,567	6,380,474	908	**hth**	+50936	**hth**	+50936	8	11	9	12	6.0		
37	PCE8165		X	8,390,109	8,392,075	1,967	**oc**	-513	CG12772	-23984	10	16	5	8	5.8		
38	PCE8166		3R	12,570,467	12,571,123	657	**Ubx**	-10101	CG31275	+5951	7	8	11	12	5.7		
39	PCE8167	Ubx S1	3R	12,589,099	12,589,755	657	**CG31275 (Ubx adj.)**	-11970	Glut3	-24295	7	8	11	12	5.7		
40	PCE8169	ftz stripes 1/5	3R	2,693,336	2,694,915	1,580	**ftz**	+3290	**Antp**	+63624	11	12	7	8	5.7		
41	PCE8170		3R	2,670,658	2,672,242	1,585	**Scr**	+2100	**Scr**	+2100	9	15	6	9	5.7	**ftz**	-19388
42	PCE8177		2R	5,634,520	5,635,604	1,085	**psq**	+4661	**psq**	+4661	8	12	7	11	5.7		
43	PCE8183		2L	7,305,525	7,305,940	416	**wg**	+4205	**wg**	+4205	5	6	12	14	5.6		
44	PCE8187		2L	8,286,022	8,287,399	1,378	**Btk29A**	+5904	**Btk29A**	+5904	9	13	7	9	5.6		
45	PCE8190		3L	6,589,453	6,590,721	1,269	**Glu-RI**	+5891	**Glu-RI**	+5891	9	12	7	9	5.6		
46	PCE8193	Kr CD2	2R	20,268,656	20,269,940	1,285	CG9380	-36249	**Kr**	-244	7	15	5	12	5.5		
47	PCE8195		3L	5,126,445	5,126,805	361	**CG32423**	+17297	**CG32423**	+17297	4	6	11	17	5.5		
48	PCE8198		2L	3,767,311	3,769,396	2,086	**bowl**	+2110	**bowl**	+2110	9	17	4	8	5.5		
49	PCE8210		3L	7,925,371	7,926,049	679	**exex**	+17651	RNaseX25	-4074	6	9	9	13	5.4		
50	PCE8214		2L	12,601,146	12,602,225	1,080	ref2	-895	CG15488	-433	8	11	7	10	5.4	**nub**	-6071
51	PCE8218		2L	10,545,226	10,547,197	1,972	**CG31721**	+7937	**CG31721**	+7937	10	14	5	7	5.3		
52	PCE8226		2L	12,541,433	12,542,145	713	**bun**	-11992	CG15489	-40512	6	9	8	13	5.2		
53	PCE8235		X	2,190,216	2,191,697	1,482	**gt**	-4481	tko	+9051	9	12	6	8	5.2		
54	PCE8237		2L	12,670,755	12,671,417	663	**pdm2**	+3280	**pdm2**	+3280	6	8	9	12	5.2		
55	PCE8258		3L	15,491,385	15,492,925	1,541	**CrebA**	+7093	**CrebA**	+7093	7	15	5	10	5.1		
56	PCE8270		3L	16,421,730	16,422,846	1,117	**argos**	+9734	**argos**	+9734	8	10	7	9	5.0		
57	PCE8275		3L	18,329,419	18,330,261	843	**grim**	-76126	**rpr**	+17021	6	10	7	12	5.0		
58	PCE8277		3R	6,448,750	6,449,993	1,244	**hth**	+8759	**hth**	+8759	6	14	5	11	5.0		
59	PCE8297		2R	20,280,374	20,281,018	645	**Kr**	+10190	CG30429	-9080	6	7	9	11	4.9		
60	PCE8306		3L	12,278,550	12,279,346	797	CG4328	-28041	**CG32105**	-7436	6	9	8	11	4.9		
61	PCE8307		3L	5,580,997	5,581,649	653	CG12756	-13449	**CG5249**	-8641	6	7	9	11	4.9		
62	PCE8309		2L	3,825,809	3,827,419	1,611	**slp1**	+7561	**slp2**	-1991	8	13	5	8	4.9		
63	PCE8314		2L	3,842,537	3,843,621	1,085	**slp2**	+13127	CG3964	-11628	6	12	6	11	4.8		

	CRM	Overlaps known element	Chrom arm	pCRM start	pCRM end	pCRM len	5' gene	pCRM relative position	3' gene	pCRM relative position	Aligned sites	Aligned + preserved sites	Aligned site dens	Aligned + preserved site dens	z-score	Additional Gap/pair-rule gene within 20kb	pCRM relative position
64	PCE8328		2L	16,418,533	16,419,580	1,048	**BG:DS02780.1**	+8016	Idgf1	-3783	7	10	7	10	4.8		
65	PCE8331		3L	5,582,709	5,583,340	632	CG12756	-15161	**CG5249**	-6950	5	8	8	13	4.8		
66	PCE8332		3R	2,725,376	2,726,195	820	**Antp**	+32344	**Antp**	+32344	6	9	7	11	4.8		
67	PCE8338		3R	3,987,824	3,989,532	1,709	**grn**	+17647	**grn**	+17647	8	13	5	8	4.7		
68	PCE8348		3L	18,966,181	18,967,380	1,200	**nkd**	+26830	**nkd**	+26830	7	11	6	9	4.7		
69	PCE8355		3R	6,421,647	6,422,583	937	**hth**	+8827	**hth**	+8827	6	10	6	11	4.7		
70	PCE8356		3L	22,244,275	22,244,894	620	**Ten-m**	+80890	CG32450	-2161	6	6	10	10	4.7		
71	PCE8358		3R	26,740,914	26,742,495	1,582	**Ptx1**	+2496	**Ptx1**	+2496	8	12	5	8	4.7		
72	PCE8361	Ubx BRE	3R	12,526,665	12,527,949	1,285	**Ubx**	+32417	**Ubx**	+32417	6	13	5	10	4.6		
73	PCE8367		2R	4,771,288	4,771,881	594	CG10459	+3018	**dap**	-1074	5	7	8	12	4.6		
74	PCE8369		3L	14,540,753	14,541,382	630	**HGTX**	+7066	**HGTX**	+7066	6	6	10	10	4.6		
75	PCE8370		3L	2,395,158	2,396,393	1,236	CG13800	+12412	**CG32306**	-13538	5	14	4	11	4.6		
76	PCE8391		3L	5,254,002	5,254,895	894	**CG32423**	-16750	lama	+55892	6	9	7	10	4.5		
77	PCE8394	Kr 730	2R	20,266,323	20,267,047	725	CG9380	-33916	**Kr**	-3137	6	7	8	10	4.5		
78	PCE8398		3R	2,770,846	2,771,901	1,056	**Antp**	+12307	**Antp**	+12307	7	9	7	9	4.5		
79	PCE8401		2L	12,660,502	12,661,614	1,113	CG15485	-2463	**pdm2**	+5861	6	11	5	10	4.5		
80	PCE8408		X	8,379,690	8,381,014	1,325	**oc**	+8582	**oc**	+8582	5	14	4	11	4.4		
81	PCE8415		3R	13,867,601	13,868,164	564	CG7794	+18158	**htl**	+6934	5	6	9	11	4.4		
82	PCE8417		2L	587,804	588,638	835	**Gsc**	+7714	**Gsc**	+7714	6	8	7	10	4.4		
83	PCE8418		3R	18,950,000	18,950,634	635	CG31457	-5638	**hh**	+7739	5	7	8	11	4.4	**cenB1A**	12397
84	PCE8425		2R	18,693,096	18,694,318	1,223	**retn**	+16917	CG5411	-6825	7	10	6	8	4.4		
85	PCE8439		X	4,770,587	4,771,859	1,273	CG12680	+32240	**ovo**	-17051	7	10	5	8	4.3		
86	PCE8444		3L	18,330,763	18,332,045	1,283	**grim**	-77470	**rpr**	+15237	7	10	5	8	4.3		
87	PCE8450		3L	5,141,131	5,141,793	663	**CG32423**	+2971	CG10677	-438	5	7	8	11	4.3		
88	PCE8458		3L	19,101,833	19,102,666	834	**fz2**	+6194	**fz2**	+6194	5	9	6	11	4.2		
89	PCE8464		3L	17,314,105	17,314,815	711	**tap**	+5577	**Cad74A**	+13577	6	6	8	8	4.2		
90	PCE8483		2L	8,265,854	8,267,283	1,430	**Btk29A**	+2646	**Btk29A**	+2646	4	15	3	10	4.1		
91	PCE8493		3R	6,403,852	6,405,604	1,753	**hth**	+25806	**hth**	+25806	7	12	4	7	4.1		
92	PCE8494		3R	7,931,641	7,932,680	1,040	CG31361	+7815	**CG31361**	+7815	6	9	6	9	4.1		
93	PCE8495		2L	5,214,677	5,215,845	1,169	CG6514	+3847	**tkv**	+14084	6	10	5	9	4.1		
94	PCE8501		2L	5,247,719	5,248,767	1,049	**tkv**	+10898	Cyp4ac1	-7804	6	9	6	9	4.1		
95	PCE8511		3R	6,469,170	6,470,599	1,430	**hth**	-4766	CG6465	+32311	7	10	5	7	4.0		
96	PCE8512	pdm2 neurogenic	2L	12,663,453	12,664,721	1,269	**pdm2**	+2754	**pdm2**	+2754	5	12	4	9	4.0		
97	PCE8513		3L	14,550,945	14,551,746	802	**HGTX**	-2497	Cyp314a1	-16963	5	8	6	10	4.0		
98	PCE8515		2L	16,390,610	16,392,235	1,626	**BG:DS02780.1**	+34314	**BG:DS02780.1**	+34314	7	11	4	7	4.0		
99	PCE8519		3L	8,975,309	8,975,873	565	**Doc2**	+2077	**Doc2**	+2077	5	5	9	9	4.0	**Doc3**	11402
100	PCE8520		2L	12,080,772	12,081,448	677	**prd**	-5445	CG5325	-1193	4	8	6	12	4.0		
101	PCE8521		2L	7,252,370	7,253,008	639	CG31909	+2569	Wnt4	+16391	5	6	8	9	4.0	**Ndae1**	-19639
102	PCE8528		X	14,366,706	14,367,311	606	**NetA**	+17535	**NetA**	+17535	4	7	7	12	4.0		
103	PCE8531		3R	6,363,866	6,364,968	1,103	CG31394	-8970	**hth**	+66442	6	9	5	8	4.0		
104	PCE8533		3R	24,402,963	24,403,946	984	**fkh**	-2792	Noa36	+10421	6	8	6	8	3.9		
105	PCE8536		3R	12,764,472	12,765,970	1,499	**Abd-B**	+4036	**Abd-B**	+4036	7	10	5	7	3.9		
106	PCE8547		X	3,111,977	3,112,560	584	dnc	+19670	**dm**	-19060	5	5	9	9	3.9		
107	PCE8548		3R	4,509,837	4,510,965	1,129	CG11755	+5816	**hb**	+9372	6	9	5	8	3.9		
108	PCE8553		2R	16,289,218	16,289,840	623	**CG18375**	+4664	**CG18375**	+4664	4	7	6	11	3.9		
109	PCE8561	CE8023	3R	2,594,997	2,596,453	1,457	**Ama**	+5869	**Dfd**	-21105	6	11	4	8	3.9	**bcd**	-9797
110	PCE8564		X	9,441,828	9,443,049	1,222	**btd**	+8657	Sp1	-48893	5	11	4	9	3.8		
111	PCE8570		2L	8,797,367	8,797,855	489	CG9468	-23885	**SoxN**	-20000	4	5	8	10	3.8		
112	PCE8573		3R	6,374,489	6,376,060	1,572	**hth**	+55350	**hth**	+55350	5	13	3	8	3.8		
113	PCE8577		3R	12,568,810	12,569,623	814	**Ubx**	-8444	CG31275	+7451	6	6	7	7	3.8		
114	PCE8583		2L	11,452,927	11,453,606	680	**salm**	-18453	**sala**	-21453	5	6	7	9	3.8		
115	PCE8584		3R	6,351,140	6,351,819	680	**hth**	+80270	CG31394	+3077	5	6	7	9	3.8		
116	PCE8597		2L	12,612,518	12,613,584	1,067	**nub**	+5301	**nub**	+5301	6	8	6	7	3.8		
117	PCE8601		3R	6,385,173	6,385,786	614	**hth**	+45624	**hth**	+45624	5	5	8	8	3.7		
118	PCE8602		3L	5,182,947	5,184,150	1,204	**CG32423**	+24805	CG4669	-9636	4	12	3	10	3.7		
119	PCE8611		2L	6,219,157	6,220,296	1,140	**Ugt37b1**	+1893	CG9486	-27794	3	13	3	11	3.7		
120	PCE8615		3L	20,627,921	20,628,582	662	**kni**	-14020	CG13253	+9154	4	7	6	11	3.7		
121	PCE8624		3R	13,644,559	13,645,270	712	**CG31246**	+7279	**CG31246**	+7279	5	6	7	8	3.7		
122	PCE8626		X	4,811,169	4,812,137	969	**ovo**	+6323	CG32767	+9550	4	10	4	10	3.7		
123	PCE8634		3L	5,949,714	5,950,761	1,048	**CG10479**	-14401	CG32406	-5760	5	9	5	9	3.6		
124	PCE8640		3R	8,901,043	8,901,919	877	**sim**	+2901	**sim**	+2901	6	6	7	7	3.6		
125	PCE8645		3R	6,414,925	6,415,526	602	**hth**	+15884	**hth**	+15884	4	6	7	10	3.6		
126	PCE8650		3R	13,897,919	13,898,867	949	**htl**	-19198	CG14317	-7990	5	8	5	8	3.6		

	CRM	Overlaps known element	Chrom arm	pCRM start	pCRM end	pCRM len	5' gene	pCRM relative position	3' gene	pCRM relative position	Aligned sites	Aligned + preserved sites	Aligned site dens	Aligned + preserved site dens	z-score	Additional Gap/pair-rule gene within 20kb	pCRM relative position
127	PCE8653		3R	675,079	675,817	739	CG14659	-18095	**opa**	-2748	5	6	7	8	3.6		
128	PCE8655		3R	20,555,177	20,555,870	694	**tok**	+18743	**tok**	+18743	4	7	6	10	3.6	**tld**	-10935
129	PCE8656		X	2,168,910	2,169,699	790	EG:BACH7M4.4	-1239	CG32797	+12778	4	8	5	10	3.6	**gt**	16825
130	PCE8665		3R	3,995,668	3,996,708	1,041	**grn**	+10471	**grn**	+10471	6	7	6	7	3.5		
131	PCE8669		3L	7,924,102	7,924,570	469	**exex**	+16382	RNaseX25	-5553	4	4	9	9	3.5		
132	PCE8674		2L	706,502	707,044	543	**ds**	+9589	**ds**	+9589	4	5	7	9	3.5		
133	PCE8679	slp1 early	2L	3,816,761	3,817,475	715	CG3407	-10785	**slp1**	-773	4	7	6	10	3.5	**slp2**	-12649
134	PCE8680		3L	12,536,054	12,536,768	715	CG10632	-5985	**ara**	-1452	4	7	6	10	3.5		
135	PCE8698		2L	11,476,016	11,477,334	1,319	**sala**	+957	CG6488	+10656	5	10	4	8	3.4		
136	PCE8700		2L	3,629,528	3,630,422	895	**for**	+2067	**for**	+2067	5	7	6	8	3.4		
137	PCE8723		2L	18,597,305	18,598,229	925	**MESR3**	+1966	**MESR3**	+1966	5	7	5	8	3.4		
138	PCE8727		3R	9,152,882	9,153,545	664	**CG32473**	+17756	CG8795	+6359	4	6	6	9	3.3		
139	PCE8737		3R	15,770,642	15,771,644	1,003	**mira**	-9783	CG4783	-2564	4	9	4	9	3.3		
140	PCE8749		3R	12,547,814	12,548,778	965	**Ubx**	+11588	**Ubx**	+11588	5	7	5	7	3.3		
141	PCE8753		2R	15,156,019	15,156,707	689	CG16898	-87320	**18w**	-18515	4	6	6	9	3.2		
142	PCE8757		2R	9,860,958	9,861,476	519	**kn**	+1722	CG12856	-528	4	4	8	8	3.2		
143	PCE8765		2L	12,649,899	12,650,970	1,072	**pdm2**	+3201	**pdm2**	+3201	4	9	4	8	3.2		
144	PCE8776		2L	600,954	601,726	773	**Gsc**	-4602	CG13689	-3050	5	5	6	6	3.1		
145	PCE8789		3R	10,412,310	10,413,906	1,597	CG7987	-10722	stumps	-4296	5	10	3	6	3.1	**CG8066**	-17760
146	PCE8792		2R	9,858,755	9,859,662	908	**kn**	+3925	CG12856	-2342	5	6	6	7	3.1		
147	PCE8804		3R	14,816,598	14,819,095	2,498	**gukh**	+6835	**gukh**	+6835	6	11	2	4	3.0		
148	PCE8819		2L	5,226,813	5,228,290	1,478	**tkv**	+1639	**tkv**	+1639	5	9	3	6	3.0		
149	PCE8836		2L	18,855,255	18,855,935	681	**tup**	+3404	**tup**	+3404	4	5	6	7	2.9		
150	PCE8846		3R	12,776,906	12,777,842	937	**Abd-B**	+6900	**Abd-B**	+6900	4	7	4	7	2.9		
151	PCE8853		3R	26,747,640	26,748,342	703	**Ptx1**	+9222	CG15549	+3253	4	5	6	7	2.8		
152	PCE8855		3R	6,452,909	6,453,618	710	**hth**	+10786	**hth**	+10786	4	5	6	7	2.8		
153	PCE8859		3R	6,450,885	6,451,851	967	**hth**	+10894	**hth**	+10894	4	7	4	7	2.8		
154	PCE8863		X	6,787,027	6,788,250	1,224	**CG14427**	+6044	nullo	-1546	5	7	4	6	2.8		
155	PCE8867		3L	14,119,875	14,120,593	719	Sox21b	-39671	**D**	+6907	4	5	6	7	2.8		
156	PCE8882		3R	14,837,689	14,838,329	641	**gukh**	+27926	**gukh**	+27926	4	4	6	6	2.7		
157	PCE8888		3L	5,937,286	5,937,942	657	**CG10479**	-1973	CG32406	-18579	4	4	6	6	2.6		
158	PCE8898		X	2,719,029	2,719,695	667	**rst**	+10908	**rst**	+10908	4	4	6	6	2.6		
159	PCE8900		2R	20,176,889	20,177,557	669	CG30430	+3888	**Tkr**	+5554	4	4	6	6	2.6		
160	PCE8901		2L	14,469,880	14,470,807	928	BG:DS08340.1	-36912	**noc**	-2206	4	6	4	6	2.6		
161	PCE8908		3R	4,019,218	4,020,017	800	**grn**	-12039	CG7800	-23697	4	5	5	6	2.6		
162	PCE8914		3R	5,682,722	5,683,950	1,229	CG12806	+9908	CG12806	+9908	3	9	2	7	2.5	**CG12802**	-18690
163	PCE8915		3R	12,378,599	12,379,564	966	CG14888	+2823	**CG14889**	-2330	4	6	4	6	2.5		
164	PCE8916		2L	3,833,662	3,834,807	1,146	**slp2**	+4252	CG3964	-20442	4	7	3	6	2.5	**slp1**	15414
165	PCE8925		3R	18,927,474	18,928,569	1,096	CG13830	+524	CG13830	+524	5	5	5	5	2.4	**cenB1A**	-7306
166	PCE8938		3L	5,930,568	5,931,337	770	**CG10479**	+3976	**CG10479**	+3976	4	4	5	5	2.3		
167	PCE8950		2L	16,386,675	16,387,635	961	**BG:DS02780.1**	+38914	**BG:DS02780.1**	+38914	4	5	4	5	2.2		
168	PCE8963		2R	10,196,306	10,197,443	1,138	**CG11798**	+3666	**CG11798**	+3666	4	5	4	4	2.0		
169	PCE8976		X	4,772,129	4,773,172	1,044	CG12680	+33782	**ovo**	-15738	1	6	1	6	1.1		
