# Supplementary material for: Computational identification of developmental enhancers: conservation and function of transcription factor binding-site clusters in Drosophila melanogaster and Drosophila pseudoobscura
Source: Genome Biol. 2004 Aug 20;5(9):R61. doi: 10.1186/gb-2004-5-9-r61 (PMC522868; doi:10.1186/gb-2004-5-9-r61)
Supplement: Additional data file 11 — All new pCRMs from genome-wide eCIS-ANALYST located between 20 kb and 50 kb from gene with anterior-posterior pattern [file gb-2004-5-9-r61-s11.pdf]

	CRM	Overlaps	Chrom arm	pCRM start	pCRM end	pCRM len	5' gene	pCRM relative position	3' gene	pCRM relative position	Aligned sites	Aligned + preserved sites	Aligned site dens	Aligned + preserved site dens	z-score	Additional Gap/pair-rule gene within 20kb	pCRM relative position
1	PCE8079		3L	19,144,705	19,145,326	622	**fz2**	-25200	mRpL21	+58610	9	9	14	14	7.5		
2	PCE8101		3L	19,148,678	19,149,172	495	**fz2**	-29173	mRpL21	+54764	7	7	14	14	6.7		
3	PCE8111		3L	8,612,337	8,613,016	680	CG6486	+4104	**h**	-21652	8	9	12	13	6.5		
4	PCE8152		2L	16,363,535	16,364,908	1,374	BG:DS09218.5	+19533	**BG:DS02780.1**	+61641	8	16	6	12	6.0		
5	PCE8164		2L	8,839,488	8,841,115	1,628	**SoxN**	+21633	CG32986	-13922	10	14	6	9	5.8		
6	PCE8227		X	9,459,015	9,459,728	714	**btd**	+25844	Sp1	-32214	6	9	8	13	5.2		
7	PCE8236		3L	14,425,844	14,427,189	1,346	**CG9598**	-59632	CG9587	+34939	7	14	5	10	5.2		
8	PCE8240		3R	12,696,513	12,697,212	700	**abd-A**	-40725	CG10349	-8252	7	7	10	10	5.1		
9	PCE8255	ubx PBX	3R	12,598,962	12,599,746	785	**CG31275 (Ubx adj.)**	-21833	Glut3	-14304	7	8	9	10	5.1		
10	PCE8286		3R	25,392,093	25,392,797	705	**Dr**	+20619	CG7567	+10391	6	8	9	11	4.9		
11	PCE8292		2L	14,441,774	14,442,414	641	BG:DS08340.1	-8806	**noc**	-30599	6	7	9	11	4.9		
12	PCE8313		X	2,779,499	2,781,121	1,623	**rst**	-48896	CG4116	+43212	6	16	4	10	4.8		
13	PCE8316		3L	18,303,559	18,304,889	1,331	**grim**	-50266	**rpr**	+42393	8	11	6	8	4.8		
14	PCE8346		2L	12,569,943	12,571,337	1,395	**bun**	-40502	CG15489	-11320	6	14	4	10	4.7		
15	PCE8359		3R	3,961,949	3,962,994	1,046	CG7891	+9599	**grn**	+44185	8	8	8	8	4.7		
16	PCE8366		3L	15,357,679	15,358,733	1,055	**Toll-6**	+72101	CG7804	+64256	6	11	6	10	4.6		
17	PCE8372		2R	15,108,654	15,109,286	633	CG16898	-39955	**18w**	-65936	6	6	9	9	4.6		
18	PCE8377		X	14,416,809	14,417,729	921	CG5321	+6191	**NetB**	+62559	5	11	5	12	4.6		
19	PCE8378		3L	5,114,119	5,116,458	2,340	Srp54k	+2242	**CG32423**	+27644	8	15	3	6	4.6		
20	PCE8384		3L	15,387,239	15,389,216	1,978	**Toll-6**	+101661	CG7804	+33773	7	15	4	8	4.5		
21	PCE8432		2L	21,728,314	21,729,432	1,119	**tsh**	+66599	CG11629	+1950	5	12	4	11	4.3		
22	PCE8447		3R	17,401,939	17,403,156	1,218	InR	-4724	**E2f**	+46552	6	11	5	9	4.3		
23	PCE8472		2L	12,563,964	12,564,680	717	**bun**	-34523	CG15489	-17977	4	9	6	13	4.2		
24	PCE8496		2L	11,379,043	11,379,593	551	salr	+24857	**salm**	+54881	5	5	9	9	4.1		
25	PCE8508		3L	18,317,741	18,318,637	897	**grim**	-64448	**rpr**	+28645	5	9	6	10	4.0		
26	PCE8535		3R	2,859,299	2,860,288	990	**Antp**	-33021	Sodh-1	-17769	6	8	6	8	3.9		
27	PCE8562		2L	11,385,288	11,386,373	1,086	salr	+31102	**salm**	+48101	7	7	6	6	3.9		
28	PCE8571		3R	15,106,844	15,107,813	970	CG14280	+23840	**Dl**	+44139	5	9	5	9	3.8		
29	PCE8581		2L	16,355,350	16,356,517	1,168	BG:DS09218.5	+11348	**BG:DS02780.1**	+70032	6	9	5	8	3.8		
30	PCE8588		X	15,857,208	15,857,812	605	disco-r	-2995	**disco**	+89941	3	8	5	13	3.8		
31	PCE8592		2L	3,852,586	3,853,784	1,199	**slp2**	+23176	CG3964	-1465	6	9	5	8	3.8		
32	PCE8598		2L	7,326,567	7,327,139	573	**wg**	+25247	Wnt6	-15989	4	6	7	10	3.8		
33	PCE8630		3R	888,267	888,982	716	CG2022	-73796	**corto**	+23423	5	6	7	8	3.7		
34	PCE8652		3L	18,314,668	18,316,059	1,392	**grim**	-61375	**rpr**	+31223	7	8	5	6	3.6		
35	PCE8664		2L	12,556,729	12,557,586	858	**bun**	-27288	CG15489	-25071	5	7	6	8	3.5		
36	PCE8687		X	15,920,993	15,921,765	773	disco-r	-66780	**disco**	+25988	5	6	6	8	3.5		
37	PCE8734		3R	9,766,472	9,767,187	716	**ems**	+38873	CG9929	+1956	5	5	7	7	3.3		
38	PCE8750		2L	21,722,836	21,723,621	786	**tsh**	+61121	CG11629	+7761	4	7	5	9	3.3		
39	PCE8758		3R	15,179,152	15,179,845	694	**Dl**	-27200	CG3581	+20643	4	6	6	9	3.2		
40	PCE8766		2R	6,621,834	6,622,541	708	**en**	-28911	tou	+24807	4	6	6	8	3.2		
41	PCE8823		2R	15,144,708	15,145,905	1,198	CG16898	-76009	**18w**	-29317	4	9	3	8	3.0		
42	PCE8828		3R	15,192,208	15,193,724	1,517	**Dl**	-40256	CG3581	+6764	5	9	3	6	2.9		
43	PCE8852		3R	25,333,346	25,334,048	703	CG2014	+2414	**Dr**	-37426	4	5	6	7	2.8		
44	PCE8903		2L	7,324,059	7,324,852	794	**wg**	+22739	Wnt6	-18276	4	5	5	6	2.6		
45	PCE8933		3L	19,169,035	19,170,095	1,061	**fz2**	-49530	mRpL21	+33841	4	6	4	6	2.4		
46	PCE8940		2R	10,133,926	10,135,029	1,104	hbs	+58803	**CG11798**	-57611	4	6	4	5	2.3		
47	PCE8977	CE8008	X	9,457,113	9,458,627	1,515	**btd**	+23942	Sp1	-33315	2	5	1	3	0.9		
